# Cardiovascular outcomes 50 years after antenatal exposure to betamethasone: Follow-up of a randomised double-blind, placebo-controlled trial

**DOI:** 10.1371/journal.pmed.1004378

**Published:** 2024-04-01

**Authors:** Anthony G. B. Walters, Greg D. Gamble, Caroline A. Crowther, Stuart R. Dalziel, Carl L. Eagleton, Christopher J. D. McKinlay, Barry J. Milne, Jane E. Harding

**Affiliations:** 1 Liggins Institute, University of Auckland, Auckland, New Zealand; 2 Department of Paediatrics, Child and Youth Health, University of Auckland, Auckland, New Zealand; 3 Department of Surgery, University of Auckland, Auckland, New Zealand; 4 Centre of Methods and Policy Application in Social Sciences, University of Auckland, Auckland, New Zealand; Cambridge University, UNITED KINGDOM

## Abstract

**Background:**

Antenatal corticosteroids for women at risk of preterm birth reduce neonatal morbidity and mortality, but there is limited evidence regarding their effects on long-term health. This study assessed cardiovascular outcomes at 50 years after antenatal exposure to corticosteroids.

**Methods and findings:**

We assessed the adult offspring of women who participated in the first randomised, double-blind, placebo-controlled trial of antenatal betamethasone for the prevention of neonatal respiratory distress syndrome (RDS) (1969 to 1974). The first 717 mothers received 2 intramuscular injections of 12 mg betamethasone or placebo 24 h apart and the subsequent 398 received 2 injections of 24 mg betamethasone or equivalent volume of placebo. Follow-up included a health questionnaire and consent to access administrative data sources. The co-primary outcomes were the prevalence of cardiovascular risk factors (any of hypertension, hyperlipidaemia, diabetes mellitus, gestational diabetes mellitus, or prediabetes) and age at first major adverse cardiovascular event (MACE) (cardiovascular death, myocardial infarction, coronary revascularisation, stroke, admission for peripheral vascular disease, and admission for heart failure). Analyses were adjusted for gestational age at entry, sex, and clustering. Of 1,218 infants born to 1,115 mothers, we followed up 424 (46% of survivors; 212 [50%] female) at mean (standard deviation) age 49.3 (1.0) years. There were no differences between those exposed to betamethasone or placebo for cardiovascular risk factors (159/229 [69.4%] versus 131/195 [67.2%]; adjusted relative risk 1.02, 95% confidence interval [CI] [0.89, 1.18;]; *p* = 0.735) or age at first MACE (adjusted hazard ratio 0.58, 95% CI [0.23, 1.49]; *p* = 0.261). There were also no differences in the components of these composite outcomes or in any of the other secondary outcomes. Key limitations were follow-up rate and lack of in-person assessments.

**Conclusions:**

There is no evidence that antenatal corticosteroids increase the prevalence of cardiovascular risk factors or incidence of cardiovascular events up to 50 years of age. Established benefits of antenatal corticosteroids are not outweighed by an increase in adult cardiovascular disease.

## Abstract 2

Anthony G B Walters and colleagues assess cardiovascular outcomes fifty years after antenatal exposure to betamethasone, in long-term follow-up of a randomised double-blind, placebo-controlled trial.

## Author summary

**Why was this study done?:**

- Antenatal corticosteroids (ANC) are given to pregnant persons at risk of preterm birth to reduce the risk of death and breathing difficulty for their offspring.
- Animal studies have shown later adverse effects of ANC on offspring, particularly on their cardiovascular health in adulthood.
- There is little robust evidence about long-term effects in humans.
- This study aimed to provide evidence about the long-term effects of ANC on offspring cardiovascular health in adulthood.

**What did the researchers do and find?:**

- We assessed participants at 50 years of age who had been exposed to ANC or placebo during the first randomised clinical trial of this treatment.
- We compared cardiovascular disease and cardiovascular risk factors between those exposed to ANC and placebo in 424 participants (46% of those still alive), using a questionnaire and routinely collected administrative data.
- We found no differences in cardiovascular outcomes between those exposed to ANC and those who were not.

**What do these findings mean?:**

- There is no evidence that ANC has adverse effects on the cardiovascular health outcomes of offspring up to 50 years of age.
- The main limitation was the follow-up rate of less than 50% and the use of health questionnaires and routinely collected data rather than in-person assessments.

## Introduction

Antenatal corticosteroids are recommended for women at risk of preterm birth between 24 and 34 weeks’ gestation [1,2] and have proven benefit for reducing neonatal morbidity and mortality and potentially reducing neurodevelopmental delay in childhood [3].

However, there are concerns that in utero exposure to corticosteroids could have long-term adverse effects [4]. The developmental origins of health and disease (DoHaD) hypothesis proposes that events in early life may influence long-term health [5], and one key proposed mechanism is exposure to excess corticosteroids during critical periods of development resulting in persisting changes in hypothalamic–pituitary–adrenal axis function [6,7]. In animal studies across a range of species, antenatal exposure to corticosteroids has resulted in altered cardiovascular function including elevated blood pressure (BP) and sympathetic nerve activity, changes in hepatic metabolism, glucose homeostasis, and insulin resistance [7–14]. Human observational data have variably reported either higher or unchanged BP, although most studies assessed outcomes in adolescence or early adulthood [15–17]. A small number of observational studies of those born at very low birthweight have reported no association between antenatal corticosteroid exposure and glycated haemoglobin, plasma low-density lipoprotein (LDL) cholesterol, plasma triglycerides, or body mass index (BMI) [18,19], but may be confounded by the indication for treatment or health care disparities.

A 2020 Cochrane systematic review identified only 2 randomised trials of antenatal corticosteroids that have assessed offspring outcomes in early adulthood and noted the need for further follow-up studies to investigate effects in adulthood [3]. One study assessed 81 survivors at 20 years and found lower systolic BP in corticosteroid exposed participants but no effect on diastolic BP, anthropometry, or medical history [20]. The second assessed 534 30-year-old offspring of participants in the Auckland Steroid Trial (AST), the first and one of the largest randomised controlled trials of antenatal corticosteroids for women at risk of preterm birth, undertaken December 1969 to February 1974 [21,22]. Cardiovascular risk factors were not different between groups, including BP and lipid profile, but plasma insulin at 30 min after a 75 gram oral glucose tolerance test was higher in the betamethasone-exposed group, potentially suggesting mild insulin resistance [23]. However, the prevalence of cardiovascular risk factors and events is low at this age, and follow-up at older ages is required to assess clinically relevant outcomes.

In addition, although antenatal corticosteroids reduce neonatal respiratory distress syndrome (RDS), animal studies have reported that they interrupt lung development, leading to concerns that this could increase risk of emphysema in later life [8]. Previous follow-up of the AST indicated no differences in lung function tests between treatment groups at 30 years of age, but the prevalence of chronic obstructive airways disease is also low at this age [24].

We therefore assessed the offspring of participants in the AST at 50 years of age to investigate the long-term effects of antenatal exposure to corticosteroids on cardiovascular, metabolic, and respiratory outcomes.

## Methods

### Ethics statement

Ethics approval was obtained from the Northern A Health and Disability Ethics Committee on behalf of all New Zealand regional ethics committees. Written informed consent was obtained from each participant. A waiver of consent for accessing administrative health records was approved for potential participants who had died before the current follow-up study.

### The Auckland Steroid Trial

The AST has been described elsewhere [21,22]. A double-blind, randomised, placebo-controlled trial of antenatal corticosteroids for preterm birth was performed at National Women’s Hospital, Auckland, New Zealand between December 1969 and February 1974. All women expected to deliver between 24 and less than 37 weeks’ gestation were eligible unless immediate delivery was indicated, or corticosteroids were contraindicated. Women provided oral informed consent for participation. Women were randomised to receive either betamethasone 12 mg intramuscularly (6 mg of short-acting betamethasone phosphate and 6 mg long-acting betamethasone acetate), repeated at 24 h, or an identical-appearing control (standard treatment dose). For planned preterm birth, the first injection was given 72 h before induction or cesarean delivery. The control treatment contained 6 mg cortisone acetate which has glucocorticoid potency one-seventieth of the active treatment and an identical appearance. As the initial findings indicated a reduction but not elimination of RDS in the treatment group, the dose of the betamethasone and control treatments were both doubled (twice standard treatment dose) in October 1972, after 717 women (769 infants) had been enrolled, in an attempt to further reduce rates of RDS [21]. For women in preterm labour, tocolytic therapy was administered to attempt to delay birth for 48 to 72 h from the first study injection, unless there were signs of fetal distress or amnionitis. A total of 1,115 women were enrolled and gave birth to 1,218 infants.

#### Randomisation and masking

A random-number table was used to generate the randomisation sequence. This was performed by the chief pharmacist who held the randomisation key. The pharmacy provided numbered, identical drug ampoules that contained either corticosteroids or the control treatment. Women received an intramuscular injection of the allocated treatment, followed by a further intramuscular injection of the same type after 24 h if delivery had not occurred. Participants were blinded to allocation from randomisation up to 30-year follow-up, after which 183 participants (83 in the betamethasone group and 100 in the placebo group) who wished to know their treatment allocation were informed by letter. Investigators and all study staff for the 50-year follow-up remained blinded to treatment allocation.

### 50-year follow-up

#### Participants

All surviving offspring of women who took part in the AST were eligible to participate. Potential participants were traced using details recorded in prior follow-up studies, records from the AST (date of birth, mother’s name, birthweight, gestation, and location at birth) and National Women’s Hospital records. Additional contact information was sought using the National Health Index (NHI, a unique identifier assigned to every individual interacting with the New Zealand health system) database and associated demographic details, electoral rolls, and if required, internet and social media searches.

#### Procedures

Participants were asked to complete a questionnaire, either online or in hard copy, which was based on the New Zealand health survey 2019/2020 [25] and the International Primary care Airways Guidelines (IPAG) questionnaire [26]. They were also asked to consent for the study team to access administrative datasets managed by the New Zealand Ministry of Health [National Minimum Dataset (NMDS; hospital admissions since 1988), National Non-admitted Patients Collection (NNPaC; emergency department and outpatient visits since 2006), Mortality dataset (MORT; national mortality records since 1970), Pharmaceutical dataset (PHARM; national records of community dispensing of pharmaceuticals funded by the New Zealand government since 1992)] and TestSafe (laboratory tests data for Auckland and Northland regions which include 36% of New Zealand’s population and New Zealand’s largest city where all participants were born, since 2010).

This study is reported as per the Strengthening the Reporting of Observational Studies in Epidemiology (STROBE) guideline (S1 Checklist).

#### Outcomes

Outcomes used data from the questionnaire and administrative datasets. Where these were inconsistent, or only a single data source was available, all available data were reviewed by a panel of 5 clinicians (unaware of treatment allocation) to adjudicate the outcome. The co-primary outcomes were a composite of cardiovascular risk factors (any of diabetes mellitus [questionnaire, NMDS, PHARM, NNPaC, and TestSafe data], prediabetes [questionnaire and TestSafe data], gestational diabetes mellitus [questionnaire, NMDS, PHARM, and TestSafe data], hypertension [questionnaire, NMDS, and PHARM data], or hyperlipidaemia [questionnaire, PHARM, and TestSafe data]) and age at first MACE in those alive at 28 days after birth (hospital admission in the NMDS for myocardial infarction or coronary revascularisation, peripheral vascular disease [acute limb ischemia or revascularisation], stroke, heart failure) or cardiovascular death (mortality data).

Secondary outcomes include components of the primary composite outcomes; a hierarchical, 6 step, unmatched, win ratio [27] (time to death after randomisation, time to first MACE, diagnosis of diabetes mellitus, number of admissions to hospital with respiratory illness as primary reason for admission, self-reported general health and time in hospital per 10 years alive after 1988); death from any cause; ischemic heart disease; stroke; peripheral vascular disease; overweight or obesity and BMI; composite of obstructive airways disease (at least 1 of: self-reported diagnosis of asthma, self-reported diagnosis of chronic obstructive airways disease [COPD], hospital admission with asthma, hospital admission with COPD, prescription of pharmaceuticals for asthma or COPD, International Primary care Airways Group questionnaire score >19.5 [26]). Tertiary, exploratory outcomes included atrial fibrillation, heart failure diagnosis in those alive at follow-up, diagnosis of type 2 diabetes mellitus and type 1 diabetes mellitus in those alive at follow-up. Outcome definitions and data sources are available in S2 Appendix.

### Statistical analysis

The statistical analysis plan was prepared before completion of data collection or any analyses (S1 Statistical Analysis Plan). All analyses were performed using SAS version 9.4 (SAS Institute, Cary, North Carolina, United States of America) and an intention-to-treat approach with participants analysed according to the initial treatment group to which their mother was allocated. Denominators are number of participants who consented to follow up with data available for the outcome, unless otherwise specified. There was no imputation for missing data, and participants for whom data were missing for a specific outcome were excluded from that analysis.

The sample size was limited by the number of surviving participants. For the co-primary composite outcome of cardiovascular risk factors, a total sample size of 420 would allow for detection of a 15% increase in the proportion with the outcome (relative risk (RR) = 1.33) with 90% power (β = 0.10, α = 0.05), assuming a baseline prevalence of 45% in the placebo group (estimated from the New Zealand Health Survey 2020/2021 and published data on diabetes mellitus and prediabetes at age 45 to 55 years) [28,29]. For the co-primary outcome of age at first MACE, a total sample size of 420 would allow for detection of a hazard ratio (HR) of at least 0.33 with 90% power (β = 0.10, α = 0.05), assuming a baseline prevalence of 8% in the placebo group (estimated from the New Zealand Health Survey 2020/2021 for age 45 to 55 years and adjusted for increased risk after preterm birth [28,30]).

Binary outcomes were compared between randomised groups using generalised linear mixed (GLM) modelling (binomial distribution, log-link function) to estimate RR and 95% confidence intervals (CIs) both unadjusted and adjusted for gestational age at randomisation and fetal sex. Adjustment for clustering of births from the same mother was performed using a random effects adjustment that approximated generalised estimation equations. Continuous outcomes were compared by GLM modelling (normal distribution, identity-link function) with and without the same adjustments and reported as mean difference (MD) or adjusted mean difference (aMD) with 95% CIs. Significance testing was based on Type 3 fixed effects obtained from the GLM model for the binary and continuous outcomes and using joint testing for the Cox proportional hazards model. Each of the primary outcomes was tested at the 5% significance level. No adjustment to the significance level was made for secondary or tertiary outcomes as the study was a safety analysis and prioritised identifying important adverse effects at the risk of increasing likelihood of Type 1 error with multiple comparisons.

For time-to-event analyses, a Cox proportional hazards model was used, reported as an HR with 95% CIs both unadjusted and adjusted for gestational age at randomisation and fetal sex, after verifying the assumption of proportionality following visualisation of Kaplan–Meier curves. Censoring occurred at death or on completion of follow-up.

For the hierarchical win-ratio outcome, the 6 steps in the hierarchy were assessed sequentially, starting with time to death after randomisation, followed by time to MACE, diagnosis of diabetes mellitus, number of admissions to hospital for respiratory illness, self-reported general health, and time spent in hospital per 10 years alive. For each step, participants in the treatment and control group were compared in all possible pairs to determine “wins,” “losses,” and “ties.” Pairwise comparisons with ties or missing data at any step were assessed at the next sequential step in the hierarchy. The analysis then determined the ratio of the probability of wins and losses for the treatment. The resulting odds ratio was reported with 95% CIs [27].

Prespecified subgroup analyses included: the planned study dose (standard versus twice standard dose), multiple pregnancy (singleton versus multiple), infant sex, tocolytic (ethanol versus salbutamol versus none/unknown), and the reason for risk of preterm birth. Interaction testing for subgroup analyses used the same model as the primary analysis, with the interaction between the subgroup and treatment group as an additional covariate. Subgroup analyses did not adjust for clustering to avoid overspecification of the model and failure of convergence. A post hoc subgroup analysis, requested in peer review, assessed the interaction between treatment group and gestational age at birth as both a categorical and continuous variable. Prespecified sensitivity analyses excluded participants who were randomised more than once in the same pregnancy (per protocol analysis), included additional covariates in the statistical models that were likely to be related to the outcomes of interest (BMI, sex, current socioeconomic status, gestation at birth, and birthweight z-score), excluded the second child for women who participated in more than 1 pregnancy, excluded participants for whom the reason for risk of preterm birth was listed as diabetes mellitus and participants for whom there was a known family history of diabetes mellitus (diabetes outcomes only), and excluded stillbirths and neonates who died before 28 days of age (hierarchical win ratio outcome only). We calculated birthweight z-scores using normative data from Thompson and colleagues [31].

## Results

**Fig 1:**
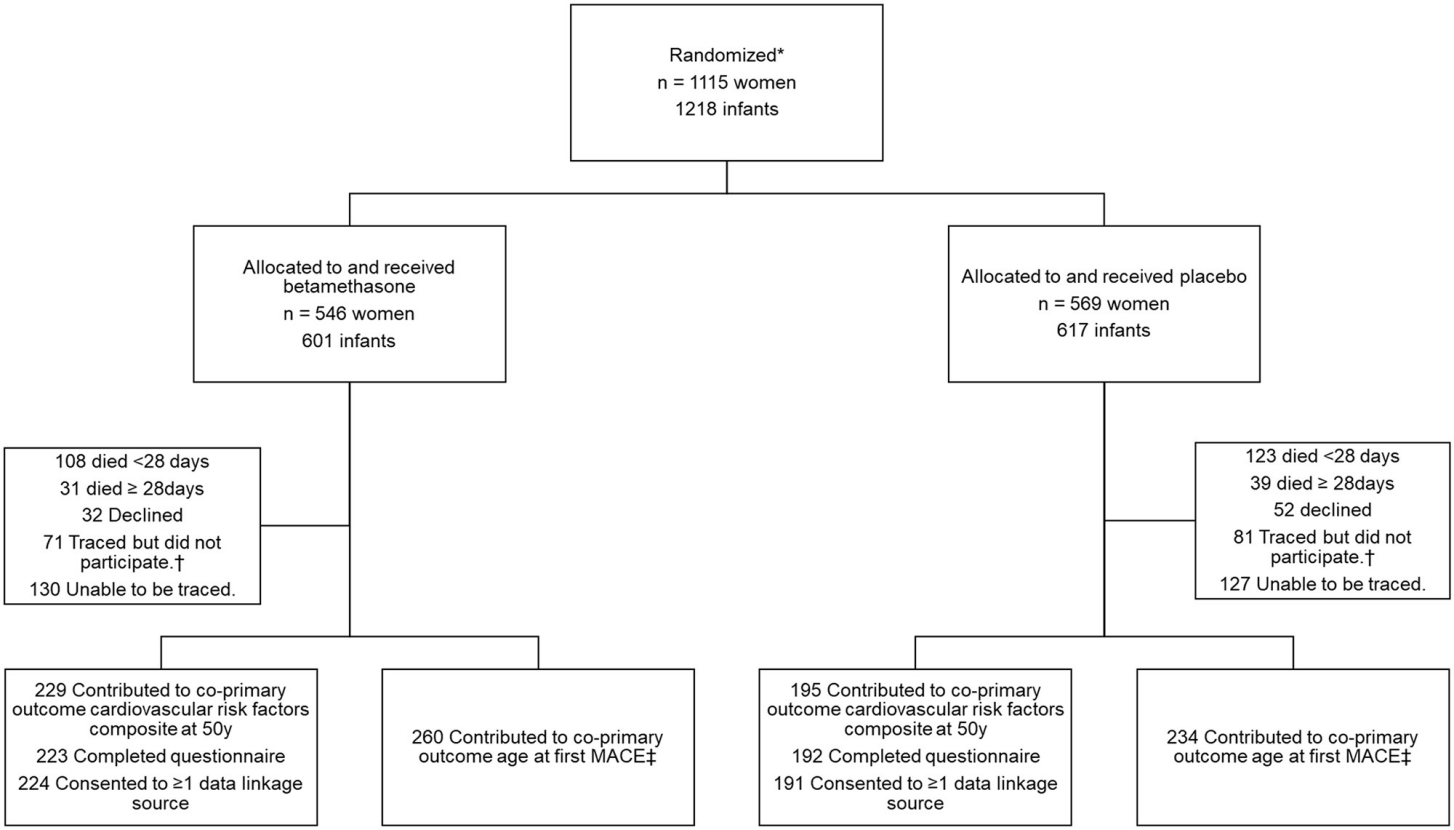
Trial flow diagram for the Auckland Steroid Trial 50-year follow-up. *Number of participants assessed for eligibility not available in original trial 1969–1974 records. †Included participants successfully traced but a response was not obtained. MACE, major adverse cardiovascular event. ‡Includes participants assessed at 50-year follow-up and participants deceased after 28 days of age for whom administrative data was available. Denominator is participants alive at 28 days (493 in betamethasone and 494 in placebo group).

In the AST, 1,218 infants were born to 1,115 women and 987 survived to 28 days (Fig 1). Between January 2020 and May 2022, 424 adult survivors were recruited to this follow-up study (46% of those presumed alive), of whom 405 (96%) both completed a questionnaire and consented to data linkage, 9 (2%) only completed a questionnaire and 10 (2%) only consented to data linkage.

Those eligible who did and did not participate had similar baseline characteristics, though participants were more likely to be female (50.0% versus 40.8%) and were less likely to be born at term (29.0% versus 40.2%) (Table 1). Those who died prior to 50-year follow-up had a lower gestational age at trial entry and at birth and were more likely to have had RDS and a low Apgar score. Baseline characteristics of participants whose mothers were randomised to betamethasone were similar to those randomised to placebo, except the betamethasone group had a lower proportion of females (45.9% versus 54.9%) (Table 2) [32].

**Table 1:** Baseline characteristics of those eligible who did and did not participate.

	Participated *N* = 424	Did not participate *N* = 493	Deceased *N* = 301
Randomised to antenatal corticosteroid	229 (54.0%)	233 (47.3%)	139 (46.2%)
Female	212 (50.0%)	201 (40.8%)	128 (42.5%)
Gestational age at entry, weeks, median (5th, 95th centile)	33.1 (27.6, 36.0)	33.4 (27.5, 37.0)	29.6 (24.0, 36.0)
Gestational age at delivery, weeks, median (5th, 95th centile)	35.0 (29.3, 40.6)	36.0 (30.7, 41.0)	31.3 (24.0, 40.0)
Multiple pregnancy			
Singleton	370 (87.3%)	437 (88.6%)	261 (86.7%)
Multiple	54 (12.7%)	56 (11.4%)	40 (13.3%)
Unplanned premature labour	354 (83.5%)	435 (88.2%)	247 (82.1%)
Planned delivery			
Hypertension-oedema-proteinuria syndromes	39 (9.2%)	27 (5.5%)	29 (9.6%)
Rhesus isoimmunisation	18 (4.3%)	14 (2.8%)	14 (4.7%)
Placenta previa	8 (1.9%)	11 (2.2%)	6 (2.0%)
Diabetes mellitus	5 (1.2%)	6 (1.2%)	5 (1.7%)
Mode of delivery			
Normal vaginal delivery	193 (45.5%)	225 (45.6%)	168 (55.8%)
Instrumental delivery	184 (43.4%)	210 (42.6%)	112 (37.2%)
Cesarean section	47 (11.1%)	58 (11.8%)	21 (7.0%)
Tocolytic used			
None	138 (32.6%)	144 (29.2%)	85 (28.2%)
Ethanol	67 (15.8%)	67 (13.6%)	50 (16.6%)
Salbutamol	207 (48.8%)	272 (55.2%)	160 (53.2%)
Both	12 (2.8%)	10 (2.0%)	6 (2.0%)
Treatment dose of betamethasone or placebo			
Standard dose (2 doses of 12 mg)	268 (63.2%)	298 (60.5%)	203 (67.4%)
Twice standard dose (2 doses of 24 mg)	156 (36.8%)	195 (39.6%)	98 (32.6%)
Term delivery	123 (29.0%)	198 (40.2%)	34 (11.3%)
Birthweight, grams, mean (SD)	2,317 (747)	2,483 (717)	1,659 (833)
Birthweight Z score, mean (SD)	−0.36 (0.98)	−0.42 (0.96)	−0.31 (1.28)
5-min Apgar score <7	53 (12.6%)	59 (12.1%)	92 (46.0%)
RDS	37 (8.7%)	30 (6.1%)	75 (24.9%)

mg, milligrams; RDS, respiratory distress syndrome; SD, standard deviation.

**Table 2:** Baseline and adult characteristics of participants whose mothers were randomised to betamethasone and placebo.

	Betamethasone *N* = 229	Placebo *N* = 195
**Maternal characteristics**		
Gestational age at entry, weeks, median (5th, 95th centile)	32.9 (27.6, 36.0)	33.4 (27.4, 36.0)
Gestational age at delivery, weeks, median (5th, 95th centile)	34.9 (29.0, 40.6)	35.0 (30.0, 40.9)
Multiple pregnancy		
Singleton	195 (85.2%)	175 (89.7%)
Multiple	34 (14.9%)	20 (10.3%)
Unplanned premature labour	193 (84.3%)	161 (82.6%)
Planned delivery		
Hypertension-oedema-proteinuria syndromes	20 (8.7%)	19 (9.7%)
Rhesus isoimmunisation	10 (4.4%)	8 (4.1%)
Placenta previa	4 (1.8%)	4 (2.1%)
Diabetes mellitus	2 (0.9%)	3 (1.5%)
Mode of delivery		
Normal vaginal delivery	102 (44.5%)	91 (46.7%)
Instrumental delivery	104 (45.4%)	80 (41.0%)
Cesarean section	23 (10.0%)	24 (12.3%)
Tocolytic used		
None	73 (31.9%)	65 (33.3%)
Ethanol	38 (16.6%)	29 (14.9%)
Salbutamol	111 (48.5%)	96 (49.2%)
Both	7 (3.1%)	5 (2.6%)
Treatment dose of betamethasone or placebo		
Standard dose (2 doses of 12 mg)	145 (63.3%)	123 (63.1%)
Twice standard dose (2 doses of 24 mg)	84 (36.7%)	72 (36.9%)
Multiple randomisation in same pregnancy†	6 (2.6%)	4 (2.1%)
Multiple randomisation in different pregnancies	6 (2.6%)	4 (2.1%)
**Neonatal characteristics**		
Female	105 (45.9%)	107 (54.9%)
Term delivery	63 (27.5%)	60 (30.8%)
Birthweight, grams, mean, (SD)	2,313 (776)	2,322 (714)
Birthweight Z score, mean (SD)	−0.32 (0.93)	−0.42 (1.03)
5-min Apgar score <7	31 (13.5%)	22 (11.5%)
RDS	15 (6.6%)	22 (11.3%)
**Adult characteristics**		
Age at entry to follow-up study, years, mean (SD)	49.3 (1.0)	49.3 (1.0)
Age at most recent administrative data, years, mean (SD)	50.4 (1.0)	50.4 (1.0)
Ethnicity*		
Māori	43 (18.8%)	54 (27.7%)
European	164 (71.6%)	132 (67.7%)
Pacific	15 (6.6%)	5 (2.6%)
Asian	0 (0%)	1 (0.5%)
Other	1 (0.4%)	0 (0%)
Unknown	6 (2.6%)	3 (1.5%)
Smoking status		
Non-smoker	124 (55.9%)	98 (51.9%)
Previously smoked	64 (28.8%)	69 (36.5%)
Currently smokes	34 (15.3%)	22 (11.6%)
Unblinding at 30-year follow-up	68 (29.7%)	68 (34.9%)

*Self-reported ethnicity. If multiple ethnicities were recorded, ethnicity was prioritised as Māori, Pacific, Asian, Other, and European [32].

^†^Three participants had received betamethasone on first randomisation followed by placebo when randomised later in the pregnancy, 3 received placebo followed by betamethasone, 3 received betamethasone on both occasions, and 1 received placebo on both occasions.

mg, milligrams; RDS, respiratory distress syndrome; SD, standard deviation.

The co-primary composite outcome of cardiovascular risk factors occurred in 159/229 (69.4%) of the betamethasone group and 131/195 (67.2%) of the placebo group (GLM aRR 1.02, 95% CI [0.89, 1.18]; *p* = 0.735). The co-primary outcome of age at first MACE was (median, 5th, 95th centile) 41.8 years (20.5, 48.5) in the betamethasone group and 44.0 years (24.2, 50.3) in the placebo group (Cox aHR 0.58, 95% CI [0.23, 1.49]; *p* = 0.261), although the proportion of participants with any event was low (1.4% betamethasone group; 2.4% placebo group) (Table 3).

**Table 3:** Primary and secondary outcomes.

Outcome	Betamethasone	Placebo	Unadjusted RR, HR, win ratio, or MD (95% CI)	Adjusted* RR, HR, or MD (95% CI)	*P* value (adjusted*)
Cardiometabolic risk factor composite, *n/N*	159/229 (69.4%)	131/195 (67.2%)	RR 1.03 (0.91, 1.18)	RR 1.02 (0.89, 1.18)	0.735
Age at first MACE, years, median (5th, 95th centile)	41.8 (20.5, 48.5)	44.0 (24.2, 50.3)	HR 0.58 (0.23, 1.47)	HR 0.58 (0.23, 1.49)	0.261
Diabetes mellitus, prediabetes or gestational diabetes mellitus, *n/N*	48/229 (21.0%)	41/195 (21.0%)	RR 1.00 (0.69, 1.45)	RR 1.00 (0.67, 1.49)	0.981
Diabetes mellitus, *n/N*	15/229 (6.6%)	21/195 (10.8%)	RR 0.61 (0.32, 1.15)	RR 0.59 (0.29, 1.19)	0.129
Prediabetes, *n/N*	30/228 (13.2%)	18/194 (9.3%)	RR 1.42 (0.82, 2.47)	RR 1.42 (0.78, 2.59)	0.234
Gestational diabetes mellitus, *n/N*	7/105 (6.7%)	3/106 (2.8%)	RR 2.36 (0.62, 8.93)	RR 2.71 (0.45, 16.4)	0.214
Hypertension, *n/N*	72/228 (31.6%)	64/195 (32.8%)	RR 0.96 (0.73, 1.27)	RR 0.96 (0.72, 1.28)	0.758
Hyperlipidaemia, *n/N*	131/228 (57.5%)	112/194 (57.7%)	RR 1.00 (0.84, 1.17)	RR 0.98 (0.82, 1.17)	0.7880
At least 1 admission for MACE including cardiovascular death, *n/N*	7/493 (1.4%)	12/494 (2.4%)	RR 0.54 (0.22, 1.34)	RR 0.55 (0.22, 1.39)	0.201
Cardiovascular death, *n/N*	2/493 (0.4%)	2/494 (0.4%)	RR 1.00 (0.14, 7.10)	RR 0.97 (0.13, 7.16)	0.977
Age at cardiovascular death, years, median (5th, 95th centile)	33.1 (20.5, 45.8)	46.9 (46.0, 47.7)	HR 1.00 (0.14, 7.07)	HR 0.96 (0.13, 6.84)	0.965
At least 1 admission for myocardial infarction or coronary revascularisation, *n/N*	5/493 (1.0%)	4/494 (0.8%)	RR 1.25 (0.34, 4.64)	RR 1.29 (0.34, 4.93)	0.705
Age at first admission for myocardial infarction or coronary revascularisation, years, median (5th, 95th centile)	44.6 (41.4, 45.2)	40.9 (26.4, 47.1)	HR 1.25 (0.34, 4.66)	HR 1.29 (0.34, 4.83)	0.706
At least 1 admission for myocardial infarction, *n/N*	4/493 (0.8%)	2/494 (0.4%)	RR 2.00 (0.37, 10.91)	RR 2.09 (0.37, 11.78)	0.400
Age at first admission for myocardial infarction, years, median (5th, 95th centile)	43.7 (41.4, 45.2)	41.9 (37.6, 46.2)	HR 2.01 (0.37, 10.96)	HR 2.09 (0.38, 11.47)	0.397
At least 1 admission for coronary revascularisation, *n/N*	1/493 (0.2%)	4/494 (0.8%)	RR 0.25 (0.03, 2.24)	RR 0.28 (0.03, 2.63)	0.260
Age at first admission for coronary revascularisation, years, median (5th, 95th centile)	44.6 (44.6, 44.6)	40.9 (26.4, 47.1)	HR 0.25 (0.03, 2.23)	HR 0.28 (0.03, 2.51)	0.254
At least 1 admission for peripheral vascular disease, *n/N*	0/493 (0%)	0/494 (0%)	NE	NE	NE
Age at first admission for peripheral vascular disease, years, median (5th, 95th centile)	NA	NA	NE	NE	NE
At least 1 admission for stroke, *n/N*	1/493 (0.2%)	2/494 (0.4%)	RR 0.50 (0.05, 5.52)	RR 0.52 (0.04, 5.99)	0.592
Age at first admission for stroke, years, median (5th, 95th centile)	47.0 (47.0, 47.0)	48.9 (47.6, 50.3)	HR 0.50 (0.05, 5.52)	HR 0.52 (0.05, 5.73)	0.590
At least 1 admission for heart failure, *n/N*	3/493 (0.6%)	7/494 (1.4%)	RR 0.43 (0.11, 1.65)	RR 0.43 (0.11, 1.69)	0.221
Age at first heart failure admission, years, median (5th, 95th centile)	41.8 (35.7, 48.5)	40.3 (24.2, 47.1)	HR 0.43 (0.11, 1.65)	HR 0.43 (0.11, 1.65)	0.216
Death from any cause, *n/N*	139/601 (23.1%)	165/617 (26.7%)	RR 0.86 (0.71, 1.05)	RR 0.89 (0.74, 1.08)	0.236
Win ratio hierarchical outcome	Treatment wins = 110,274	Placebo wins = 93,728	Win ratio† 1.18 (0.98, 1.42)	NA	0.088
Time to death after randomisation, days, median (5th, 95th centile)	10.5 (0.4, 12,850)	4.0 (0.3, 16,902)	HR 0.84 (0.67, 1.05)	HR 0.82 (0.65, 1.03)	0.084
Time to MACE after randomisation (excluding cardiovascular death), days, median, (5th, 95th centile)	15,351 (13,132, 17,718)	16,064 (8,874, 18,444)	HR 0.54 (0.20, 1.46)	HR 0.55 (0.20, 1.50)	0.245
Number of admissions to hospital with respiratory illness as primary reason for admission since 1988, mean (SD)	0.15 (0.67)	0.34 (1.91)	MD −0.19 (−0.44, 0.07)	MD −0.17 (−0.46, 0.11)	0.213
Self-reported general health, *n/N*					0.579
Excellent	31 (13.9%)	35 (18.3%)	NA	NA	
Very good	74 (33.2%)	67 (35.1%)			
Good	81 (36.3%)	65 (34.0%)			
Fair	29 (13.0%)	20 (10.5%)			
Poor	8 (3.6%)	4 (2.1%)			
Self-reported general health fair/poor, *n/N*	37/223 (16.6%)	24/191 (12.6%)	RR 1.32 (0.82, 2.13)	RR 1.37 (0.82, 2.29)	0.216
Time in hospital after 1988, days per 10 years alive, median (5th, 95th centile)	2.0 (0.0, 23.0)	2.6 (0.0, 31.6)	MD −6.5 (−15.2, 2.1)	MD −6.2 (−15.7, 3.2)	0.181
Ischemic heart disease, *n/N*	5/229 (2.2%)	9/195 (4.6%)	RR 0.47 (0.16, 1.39)	RR 0.46 (0.14, 1.49)	0.181
Stroke, *n/N*	1/229 (0.4%)	3/195 (1.5%)	RR 0.28 (0.03, 2.72)	RR 0.30 (0.03, 3.51)	0.316
Peripheral vascular disease, *n/N*	0/229 (0%)	0/195 (0%)	NE	NE	NE
Weight, kg, mean (SD)	88.8 (1.4)	84.9 (1.6)	MD 4.0 (−0.2, 8.1)	MD 3.0 (−1.3, 7.3)	0.160
Height, cm, mean (SD)	172.8 (0.7)	170.2 (0.8)	MD 2.5 (0.5, 4.6)	MD 1.3 (−0.4, 3.1)	0.121
BMI, kg/m^2^, mean (SD)	29.7 (6.4)	29.0 (6.2)	MD 0.7 (−0.6, 2.0)	MD 0.8 (−0.7, 2.3)	0.259
Overweight or obesity, *n/N*	152/198 (76.8%)	114/160 (71.3%)	RR 1.08 (0.95, 1.22)	RR 1.05 (0.92, 1.21)	0.432
Overweight, *n/N*	73/198 (36.9%)	59/160 (36.9%)	RR 1.00 (0.76, 1.31)	RR 0.94 (0.70, 1.27)	0.678
Obesity, *n/N*	79/198 (39.9%)	55/160 (34.4%)	RR 1.16 (0.88, 1.53)	RR 1.17 (0.87, 1.58)	0.265
Obesity class 1 (BMI 30- <35 kg/m^2^), *n/N*	45/160 (22.7%)	30/160 (18.8%)	RR 1.21 (0.8, 1.83)	RR 1.22 (0.78, 1.9)	0.350
Obesity class 2 (BMI 35- <40 kg/m^2^), *n/N*	19/198 (9.6%)	18/160 (11.3%)	RR 0.85 (0.46, 1.57)	RR 0.88 (0.46, 1.7)	0.678
Obesity class 3 (BMI ≥40 kg/m^2^), *n/N*	15/198 (7.6%)	7/160 (4.4%)	RR 1.73 (0.72, 4.16)	RR 1.86 (0.70, 4.96)	0.191
Composite of obstructive airways disease, *n/N*	77/229 (41.5%)	66/195 (38.5%)	RR 0.99 (0.76, 1.30)	RR 1.01 (0.76, 1.35)	0.930
Asthma, *n/N*	76/229 (33.2%)	65/195 (33.3%)	RR 1.00 (0.76, 1.31)	RR 1.01 (0.75, 1.34)	0.963
COPD, *n/N*	4/229 (1.8%)	4/195 (2.1%)	RR 0.85 (0.21, 3.37)	RR 1.10 (0.25, 4.75)	0.896
IPAG COPD questionnaire score >19.5, *n/N*	3/179 (1.7%)	2/147 (1.4%)	RR 1.23 (0.21, 7.32)	RR 1.22 (0.16, 9.20)	0.828
At least 1 admission for asthma or COPD, *n/N*	20/223 (9.0%)	16/188 (8.5%)	RR 1.05 (0.56, 1.98)	RR 1.12 (0.56, 2.23)	0.736
Prescriptions of pharmaceuticals for asthma or COPD, *n/N*	69/219 (31.5%)	47/182 (25.8%)	RR 1.22 (0.89, 1.67)	RR 1.29 (0.92, 1.82)	0.128

^*†*^Hierarchical win-odds ratio: 6-step, unmatched, win ratio hierarchy including time to death after randomisation; time to first MACE; diagnosis of diabetes mellitus; number of admissions to hospital with respiratory illness as primary reason for admission; self-reported general health; time in hospital per 10 years alive after 1988.

*Analyses adjusted for sex, gestational age at trial entry and for clustering.

BMI, body mass index; CI, confidence interval; cm, centimetres; COPD, chronic obstructive pulmonary disease; HR, hazard ratio; IPAG, International primary care airways group; MACE, major adverse cardiovascular event; MD, mean difference; NA, not applicable; NE, not estimable; RR, relative risk.

All of the components of the composite of cardiovascular risk factors outcome were similar between groups (Table 3). Age at first event for the components of the MACE outcome did not differ between treatment groups (Table 3).

The 6-step, unmatched win ratio was 1.18 (95% CI [0.98, 1.42]; *p* = 0.088), with a total of 110,274 wins (representing a better outcome) in the betamethasone group and 93,728 wins in the placebo group (Table 3). Each of the outcomes making up the 6 steps of the win ratio did not differ between groups (Table 3). The number of wins and losses at each step are available in Fig A in S2 Appendix.

Treatment groups did not differ in self-reported height; GLM aMD 1.3 centimetres (cm) (95% CI [−0.4, 3.1]; *p* = 0.121), weight (aMD 3.0 kilograms (kg), 95% CI [−1.3, 7.3]; *p* = 0.160), and BMI (aMD 0.8 kg/m^2^, 95% CI [−0.7, 2.3]; *p* = 0.259), nor the proportion of overweight or obesity (aRR 1.05, 95% CI [0.92, 1.21]; *p* = 0.432) (Table 3).

All-cause mortality from randomisation to 50-year follow-up was 139/601 (23.1%) in the betamethasone group and 165/617 (26.7%) in the placebo group (GLM aRR 0.89, 95% CI [0.74, 1.08]; *p* = 0.236). Causes of death more than 28 days after birth were similar between groups (Table 4).

**Table 4:** Causes of death after 28 days of age.

Cause of death	Betamethasone	Placebo*	Total
Cardiovascular	2 (6%)	2 (5%)	4 (6%)
Cerebral palsy	1 (3%)	1 (2%)	2 (3%)
Complications of diabetes mellitus	0 (0%)	2 (5%)	2 (3%)
Congenital abnormalities	2 (6%)	3 (7%)	5 (7%)
Infection	5 (16%)	8 (19%)	13 (18%)
Malignancy	4 (13%)	7 (17%)	11 (15%)
Sudden unexpected death in infancy	3 (10%)	1 (2%)	5 (7%)
Suicide	3 (10%)	2 (5%)	5 (7%)
Trauma/accidental death	5 (16%)	6 (14%)	11 (15%)
Other	2 (6%)	3 (7%)	5 (7%)
Unknown	4 (13%)	7 (17%)	11 (15%)
Total	31 (100%)	42 (100%)	73 (100%)

*Table includes 3 participants in the placebo group who died between completion of questionnaire and analysis of linked administrative data.

The composite outcome for ischemic heart disease and stroke did not differ between groups (Table 3). No participants met the definition for peripheral vascular disease.

The proportion with the composite of obstructive airways disease did not differ between the betamethasone group (77/229, 41.5%) and the placebo group (66/195, 38.5%) (GLM aRR 1.01, 95% CI [0.76, 1.35]; *p* = 0.930). The components of this composite also did not differ between groups (Table 3).

The prespecified subgroup analyses did not reveal any interactions between treatment effect and the dose of antenatal corticosteroid administered, multiple pregnancy, infant sex, tocolytic used or reason for risk of preterm birth, although the number of participants and outcomes in subgroups was very low in some cases and findings should be interpreted with caution (Table C in S2 Appendix). The win ratio did not differ markedly in sensitivity analyses excluding stillbirths or excluding deaths before 28 days (Table D in S2 Appendix). Other sensitivity analyses, including adjustment for gestation length, also did not markedly alter the findings for the primary and secondary outcomes (Table E in S2 Appendix). There was also no significant interaction between treatment effect and gestational age at delivery (post hoc analysis requested during peer review, Table F in S2 Appendix).

## Discussion

We studied cardiovascular outcomes at 50 years in 424 offspring born to mothers who participated in the first, and one of the largest, randomised controlled trials of antenatal corticosteroids prior to preterm birth. We found no difference between treatment groups for the co-primary outcomes of composite of cardiovascular risk factors or age at first MACE, or for their components. The composite outcome of cardiovascular risk factors also included prediabetes and gestational diabetes mellitus, which are associated with cardiovascular disease [33,34]. We also found no difference in a win ratio, intended to provide a more global assessment of the impact of antenatal betamethasone, incorporating both early and late mortality, along with cardiovascular events, diabetes diagnosis, respiratory admissions, self-reported general health, and time in hospital. To our knowledge, these findings represent the longest follow-up of children with antenatal exposure to betamethasone and demonstrate that at 50 years, there is no evidence of an increase in cardiovascular risk factors or adverse cardiovascular events. Thus, the substantial neonatal benefits of antenatal corticosteroids for preterm birth do not appear to be outweighed by negative later consequences [1]. Moreover, antenatal corticosteroids administered prior to preterm birth reduce early life healthcare care costs [35], and our findings suggest that they are very likely to remain cost-effective through into adulthood.

Our results are consistent with the findings in the earlier, 30-year, follow-up of offspring of participants in this clinical trial, which showed no differences between groups in rates of hypertension, systolic or diastolic BP, diabetes mellitus, or fasting lipid concentrations [23]. It is also consistent with another study following up 81 participants of a randomised controlled trial that found participants exposed to antenatal betamethasone had a lower systolic BP at 20 years than those exposed to placebo, although laboratory testing for dyslipidaemia and diabetes mellitus was not undertaken [20]. A recent systematic review of cardiovascular function after antenatal corticosteroid exposure, based predominantly on observational data, also concluded that there was no association between antenatal corticosteroid exposure and offspring BP but the evidence was too limited to draw firm conclusions about other aspects of cardiovascular function [17]. Concerns regarding premature cardiovascular disease after antenatal exposure to corticosteroids have followed the evidence of increased cardiovascular risk factors in animal models and human evidence for a potential role of the hypothalamic–pituitary–adrenal axis in cardiovascular disease pathophysiology [8–11,36]. Previous studies investigating the cardiovascular effects of antenatal corticosteroids, both follow-up of clinical trials and observational studies, have focused on secondary endpoints such as risk factors rather than adverse cardiovascular events which are clinically of greater importance [8,17,37,38]. Our findings of no difference in the prevalence of risk factors or of cardiovascular events suggest that any effect on later cardiometabolic health of exposure to a single course of antenatal corticosteroids is unlikely to be clinically important, at least up to 50 years.

There have been concerns that in utero exposure to antenatal corticosteroids may interrupt lung branching and alveolarization, potentially increasing the risk of later obstructive airways disease [8]. Consistent with this, increasing airway obstruction has been reported between 8 and 18 years in extremely preterm or low birthweight survivors, 73% of whom were exposed to antenatal corticosteroids [39]. We did not identify any difference between groups for the composite outcome of obstructive airways disease, although most participants with this outcome had asthma rather than COPD, and there were few extremely preterm infants. This finding is consistent with the lack of a difference in lung function, as assessed by spirometry, between betamethasone and placebo groups at 30 years in an earlier follow-up of participants in the AST, and between 6- and 8-year-old children exposed to single or repeat courses of antenatal corticosteroids in a more recent trial [24,40]. Although COPD is still uncommon at age 50, our findings suggest that premature COPD seems unlikely to be of clinical concern after antenatal corticosteroids.

Due to difficulties in predicting preterm birth, not all infants receiving antenatal corticosteroids will be born preterm, and recent studies have raised concerns that those born at term, who do not ultimately benefit from corticosteroid exposure may be at a small increased risk of adverse long-term outcomes compared to term-born controls who were not exposed to antenatal corticosteroids [41]. However, these cohort studies are inevitably confounded by the indications for use of antenatal corticosteroids and healthcare disparities. In the AST, where the control group had similar indications for the use of antenatal corticosteroids as those in the betamethasone group, just under one third of participants were born at term, similar to the proportion seen in current clinical practice [42]. We also found no significant interaction between treatment effect and gestational age at birth. Thus, the present study provide reassurance that use of antenatal corticosteroids for threatened or indicated preterm birth provides net clinical benefit in the short- and long-term, even though the prediction of timing of early birth remains imperfect.

Strengths of this study include that it assesses outcomes from a randomised trial, greatly reducing the risk of bias due to confounding. Observational studies on the long-term effects of antenatal corticosteroids are particularly difficult to interpret because of the confounding effects of healthcare disparities and the indication for treatment with antenatal corticosteroid.

We combined self-reported questionnaire data with information from administrative datasets to improve identification of outcomes by reducing the impact of recall bias, as self-reported data alone is known to underestimate prevalence of noncommunicable diseases such as hypertension, hyperlipidaemia, and diabetes mellitus [43,44]. However, the use of administrative data has important limitations, including the risk of outcome misclassification through miscoding at data collection, lack of specificity in outcome definitions (for example, through classifying diagnoses according to treatments), and the influence of healthcare access on likelihood of appearing in an administrative dataset. Nevertheless, there is no reason to believe that these biases differed between the randomised groups, although they could contribute bias towards a null finding.

There were a number of limitations of this study. First, the follow-up rate was relatively low (46% of those presumed to be alive, primarily driven by difficulty in tracing participants decades after the last study contact), which increases the risk of attrition bias. However, most baseline characteristics were similar between those assessed and those lost to follow-up, and there were few baseline differences between randomised groups. Further, additional adjustments and sensitivity analyses to explore these differences did not alter the findings. Second, we did not undertake in person assessment of BP and blood testing, for logistic reasons due to geographical dispersal of the cohort over 50 years, so the outcomes of hypertension, hyperlipidaemia, and diabetes may be underestimated. However, there is no reason to think this underestimate differed between randomised groups. Third, the overall event rate for the MACE was lower than anticipated, so our study had limited power to detect small differences between groups. However, given the substantial neonatal benefits of exposure to antenatal corticosteroids, small differences between groups in the primary outcomes at 50 years are unlikely to be clinically important.

Finally, there was a higher proportion of women in the placebo group than the betamethasone group, consistent with the known increased mortality of male preterm neonates, especially in the absence of antenatal corticosteroid treatment. This difference would be likely to favour the placebo group given the lower risk of cardiovascular disease in women. Adjustment for sex did not alter results and there was no evidence in subgroup analyses that sex influences the effect of betamethasone on primary or secondary outcomes. Thus, the imbalance in sex is unlikely to have affected our findings.

We found no evidence that the prevalence of cardiovascular risk factors or age at MACE is altered up to 50 years after antenatal exposure to betamethasone. Clinicians can have confidence that the established benefits of antenatal corticosteroids for preterm birth in reducing neonatal morbidity and mortality and probable benefits for childhood neurodevelopment are not outweighed by an increase in adult cardiovascular disease.

## Supporting information

S1 STROBE checklist STROBE Statement—Checklist of items that should be included in reports of *cohort studies*. (DOC)

S1 Statistical Analysis Plan The AnteNatal Corticosteroids Health Outcomes Review (ANCHOR) Study: Auckland Steroid Trial follow-up. (PDF)

S1 Appendix Supplement to statistical analysis plan: Outcome definitions. (DOCX)

S2 Appendix Table A. Tertiary outcomes. Fig A. Wins and losses for betamethasone at each step of the 6-step unmatched win ratio. Table B. Subgroup analyses for primary outcomes. Table C. Subgroup analyses for secondary outcomes. Table D. Sensitivity analysis for win ratio hierarchical outcome. Table E. Sensitivity analysis for primary and secondary outcomes. Table F. Post hoc subgroup analysis for primary outcomes and components by gestational age at delivery.
(DOCX)

## Abbreviations

aMD: adjusted mean difference
AST: Auckland Steroid Trial
BMI: body mass index
BP: blood pressure
CI: confidence interval
DoHaD: developmental origins of health and disease
GLM: generalised linear mixed
HR: hazard ratio
LDL: low-density lipoprotein
MACE: major adverse cardiovascular event
MD: mean difference
NHI: National Health Index
RDS: respiratory distress syndrome
