# Supplementary material for: Cardiovascular outcomes 50 years after antenatal exposure to betamethasone: Follow-up of a randomised double-blind, placebo-controlled trial
Source: PLoS Med. 2024 Apr 1;21(4):e1004378. doi: 10.1371/journal.pmed.1004378 (PMC11018286; doi:10.1371/journal.pmed.1004378)
Supplement: S1 Statistical Analysis Plan — (PDF) [file pmed.1004378.s002.pdf]

The AnteNatal Corticosteroids Health Outcomes Review (ANCHOR) Study: Auckland Steroid Trial follow up

Statistical Analysis Plan

Version 1.8, 26 August 2022

SAP Author: Anthony Walters

Approved by	Signature	Date
Dist Prof Dame Jane Harding (Principal Investigator)	[signature]	31/8/22
Greg Gamble (Statistician)	[signature]	31 Aug 22

Contents

1. Scope 2

2. Study information 2

3. Background: The Auckland Steroid Trial (1969-1974) 2

4. Methods for the ANCHOR Study: follow up of the Auckland Steroid Trial F1 3

5. Study outcomes 3

6. Outcome reporting 6

7. Steering Group 6

8. General issues for statistical analysis 6

9. Power calculation 7

10. Analysis based on Self-reported Outcomes 7

11. Exploratory analyses 8

12. Descriptive analysis 8

13. Statistical Analysis 9

14. Appendix 10

12.1 Flow diagram 10

12.2 Draft Tables 12

15. References 23

1. Scope

This Statistical Analysis Plan (SAP) describes the outcomes and analyses for the long term follow up of the Auckland Steroid Trial (AST). In this plan the mothers who participated in the AST are referred to as the F0 generation, their children the F1 generation and their grandchildren the F2 generation. This SAP concerns the fifty-year follow-up of children (F1 generation) of the participants of the Auckland Steroid Trial. This follow-up study was conducted as part of the AnteNatal Corticosteroids Health Outcomes Review (ANCHOR) Study. Outcomes of the mothers who participated in the Auckland Steroid Trial (F0 generation) and their grandchildren (F2 generation) are outside of the scope of this analysis plan.

2. Study information

2.1. Aim

- To determine if antenatal corticosteroids, given to women at risk of preterm birth, have long term impacts on the health of offspring (F1).

2.2. Study hypothesis

Antenatal corticosteroids reduce neonatal mortality and morbidity without long-term adverse impacts on health and social outcomes in offspring (F1).

3. Background: The Auckland Steroid Trial (1969-1974)

A single centre, two-arm, parallel, randomised double blind placebo-controlled trial comparing treatment with antenatal corticosteroid to placebo for improving rates of respiratory distress syndrome.

3.1. Inclusion Criteria

- Women with threatened or planned preterm delivery
- Gestational age at recruitment 24 to <37 weeks
- Women with multiple pregnancies, fetal anomalies, preterm prelabour rupture of membranes, hypertensive disorders of pregnancy and diabetes mellitus were all eligible for inclusion

3.2. Exclusion criteria

- Imminent delivery
- Contraindication to corticosteroids

3.3. Randomisation, treatment allocation and masking

A random-number table was used to generate the randomisation sequence. This was performed by the chief pharmacist who held the randomisation key. The pharmacy provided numbered, identical drug ampoules that contained either corticosteroids or the control treatment. Women received an intramuscular injection of the allocated treatment, followed by a further intramuscular injection of the same type after 24 hours if delivery had not occurred. The antenatal corticosteroid treatment contained 6 mg of short-acting betamethasone phosphate and 6 mg long-acting betamethasone acetate. The control treatment contained 6 mg cortisone acetate which has a glucocorticoid potency of one seventieth of the active treatment. In October 1972, after 717 women had been enrolled, the dose of the betamethasone and control treatments were both doubled.

3.4. Co-interventions

For women in preterm labour, tocolytic therapy was administered in an attempt to delay birth for 48 to 72 hours from the first study injection unless there were signs of fetal distress or

amnionitis. The tocolytic of choice was initially intravenous infusion of ethanol, and changed to intravenous infusion of salbutamol in June 1971 after recruitment of the first 236 women. For women with preterm prelabour rupture of the membranes (PPROM) broad spectrum intravenous antibiotics were administered, typically ampicillin.

3.5. 30-year follow up

The Auckland Steroid trial F1 generation were followed up at approximately 30 years of age. Follow up of 534 participants was performed and included questionnaires; clinical assessments of cardiovascular risk factors such as height, weight, blood pressure; blood tests including morning plasma cortisol, plasma lipids, oral glucose tolerance tests with concurrent insulin measurements; DEXA (dual energy x-ray absorptiometry) bone density measurements; spirometry for lung function testing and detailed psychosocial assessments.

4. Methods for the ANCHOR Study: follow up of the Auckland Steroid Trial F1

4.1. Study design

This study consists of long term follow up of the Auckland Steroid Trial at 50 years comparing outcomes in offspring (F1) for those exposed to antenatal corticosteroid to those exposed to placebo.

Participants were blinded to allocation from randomisation up to 30-year follow up, at which point those participants who wished to have their treatment allocation disclosed were informed by letter. Investigators and all study staff for this 50-year follow up remained unaware of treatment allocation.

Follow up consists of a questionnaire based on the New Zealand Health Survey and consent to accessing routinely collected data from government agencies including the Ministry of Health (including Testsafe), Ministry of Education, New Zealand Qualifications Authority, Accident Compensation Corporation, Ministry of Justice and Statistics New Zealand.

4.2. Inclusion Criteria

Participants were eligible for inclusion if they had been born to a participant of the Auckland Steroid Trial and written informed consent was obtained to participate in the study. Participation in the 6-year follow up study or the 30-year follow up study was not a requirement for participation.

4.3. Exclusion criteria

Declined participation or unable to obtain informed consent from participant or legal guardian.

5. Study outcomes

Outcome definitions and the hierarchical approach to different data sources have been detailed in a supplementary document (S1). This supplemental document may be amended separate to this analysis plan and will be finalised after blinded review of the data with the aim of optimising the validity of outcome definitions. All outcomes for this analysis plan will use one of three denominators as indicated by the following superscript designation:

[a] Denominator will be all participants whose mothers were randomised in the original trial.

[b] Denominator will be all participants alive at 28 days of age.

[c] Denominator will be all participants who consented to this follow-up study with data available for the outcome.

[S] indicates outcomes for which a separate analysis will be undertaken using only self-reported data (see section 10).

5.1. Primary outcomes

1. Composite of cardiovascular risk factors [cS]. Any of:
    - Diabetes mellitus or prediabetes
    - Hypertension
    - Dyslipidaemia
2. Age at first major adverse cardiovascular event [bS]. Earliest of:
    - Age at first admission for myocardial infarction or coronary revascularisation
    - Age at first admission for peripheral vascular disease (acute limb ischaemia or revascularisation)
    - Age at first admission for stroke
    - Age at first heart failure admission
    - Age at cardiovascular death

5.2. Secondary outcomes

Components of the Primary outcomes

- Diabetes mellitus or prediabetes[cS]
- Diabetes mellitus[cS]
- Prediabetes[cS]
- Hypertension[cS]
- Dyslipidaemia[cS]
- Age at cardiovascular death[b]
- Proportion with cardiovascular death[b]
- Age at first admission for myocardial infarction or coronary revascularisation[bS]
- Age at first admission for myocardial infarction [bS]
- Age at first admission for coronary revascularisation [bS]
- Age at first admission for peripheral vascular disease (arterial thrombosis/embolism or need for revascularisation)[bS]
- Age at first admission for arterial thrombosis/embolism [b]
- Age at first admission for peripheral revascularisation procedure[b]
- Age at first admission for stroke[bS]
- Age at first heart failure admission[bS]
- Proportion with at least one admission for myocardial infarction or coronary revascularisation[bS]
- Proportion with at least one admission for myocardial infarction[bS]
- Proportion with at least one admission for coronary revascularisation[bS]
- Proportion with at least one admission for peripheral vascular disease (arterial thrombosis/embolism or need for revascularisation)[b]
- Proportion with at least one admission for arterial thrombosis/embolism[b]
- Proportion with at least one admission for peripheral revascularisation procedure[b]
- Proportion with at least one admission for stroke[bs]
- Proportion with at least one admission for heart failure[b]

Win Ratio Secondary Outcomes

- Hierarchical win ratio outcome[a]: 6 step, unmatched, win ratio hierarchy including time to death after randomisation; time to first major adverse cardiovascular event; diagnosis of diabetes mellitus; number of admissions to hospital with respiratory illness as primary reason for admission; self-reported general health; time in hospital per 10 years alive after 1988
- Components of the win ratio outcome
    - Time to death after randomisation[a]
    - Time to MACE (excluding cardiovascular death)[b]
    - Diagnosis of diabetes mellitus (excluding prediabetes)[c]
    - Number of admissions to hospital with respiratory illness as primary reason for admission (admissions per patient year)[b]
    - Self-reported general health[c]
    - Time in Hospital per 10 years alive after 1988[b]

Additional Secondary Outcomes

- Ischaemic heart disease[cS]
- Stroke[cS]
- Peripheral vascular disease[c]
- Overweight or obesity[cS]
- BMI (continuous)[cS]
- Death from any cause[a]
- Self-reported general health[cS]
- Rate of admissions to hospital with respiratory illness as the primary reason for admission/per patient year of followup[c]
- Chronic respiratory illness (Self-reported diagnosis of asthma or COPD, self-reported chronic respiratory symptoms, admissions for asthma or chronic obstructive pulmonary disease [COPD] or prescription of pharmaceuticals for asthma or COPD) [c]
    - Asthma[cS]
    - COPD[cS]
    - Chronic respiratory symptoms[cS]
    - Admissions for asthma or chronic obstructive pulmonary disease (COPD) [c]
    - Prescriptions of pharmaceuticals for asthma or COPD[c]
- Highest level of educational attainment[cS]

5.3. Tertiary Outcomes

General health outcomes

- Functional difficulties[cS]
- Physical activity (categorical)[cS]

Other Diabetes outcomes

- Type 2 diabetes mellitus[cS]
- Type 1 diabetes mellitus[cS]
- Gestational diabetes mellitus[cS]

Other Cardiovascular outcomes

- Heart failure[cS]
- Atrial fibrillation[cS]

Reproductive system outcomes: Male

- Number of babies ≥20 weeks' gestation fathered[cS]

Reproductive system outcomes: Female

- Age at menarche[cS]
- Number who have reached menopause and age at menopause[cS]
- Need for assisted reproductive technology[cS]
- Polycystic ovarian syndrome[cS]
- Total number of pregnancies ≥20 weeks (parity)[cS]

Mental health Outcomes

- Diagnosis or treatment of a mental health disorder[c]
- Depression[cS]
- Bipolar affective disorder[cS]
- Anxiety disorder[cS]
- Schizophrenia[cS]
- Inpatient admission for a mental health disorder (excluding mental disorders due to known physiological conditions)[c]
- Prescriptions of pharmaceuticals for mental health disorders[cS]

Other health outcomes

- Total number of fractures[cS]
- Cancer diagnosis[cS]
- Allergic condition other than asthma (diagnosis of or prescription of treatments for allergic conditions)[cS]
- Chronic kidney disease[c]
- Self-reported oral health[cS]
- Number of teeth removed for decay[cS]

Social Outcome

- Any court charges or convictions[c]
- Employment status[cS]
- Proportion with tertiary qualifications[cS]
- No secondary school qualification[cS]

6. Outcome reporting

Outcomes may not all be reported in the same publication, instead being grouped into categories for reporting. These categories will likely include cardiometabolic and respiratory outcomes, other health outcomes and socioeconomic outcomes.

7. Steering Group

Distinguished Professor Dame Jane Harding, Professor Stuart Dalziel, Dr Carl Eagleton, Professor Caroline Crowther, Associate Professor Barry Milne, Mr Greg Gamble, Associate Professor Chris Mckinlay

8. General issues for statistical analysis

4.1. Analysis software

All analyses will be performed using SAS® software version 9.4 (SAS Institute Inc., Cary, NC, USA).

4.2. Analysis approach

Analyses will be performed using an intention-to-treat approach with participants analysed according to the initial treatment group to which their mother was allocated. Denominators have been indicated above for each study outcome.

4.3. Approach for withdrawals, missing data and outliers

Data up to the point of withdrawal will be used for those participants who have withdrawn from the study unless they have withdrawn their consent for use of their data. No imputation will be performed for missing data. We do not plan to exclude outliers from the analysis. Sensitivity analyses will be performed to assess the impact of missing data on the primary and secondary outcomes.

4.4. Protocol deviations

Participants will be included in the intention-to-treat analysis, regardless of protocol deviations. A small number of women were randomised more than once within the same pregnancy. These pregnancies will be analysed in the group to which they were first randomised.

4.5. Covariates and potential confounders

Adjusted and unadjusted results will be presented for all outcomes. The results will be adjusted based on the following variables. Additional baseline variables will be included in the model if these differ markedly between treatment groups and are known to influence the particular outcome.

- Gestational age at randomisation (continuous variable)

4.6 Multiple comparisons and multiplicity

Each of the primary outcomes will be tested at the 5% significance level. No adjustment to the significance level will be made for secondary or tertiary outcomes, subgroup analyses or sensitivity analyses. The total number of tests will be reported to allow interpretation of the risk of type I error.

4.7. Clustering

Inclusion of infants from multiple (twin or triplet) pregnancies in the analysis is a potential source of clustering. To account for the effect of clustering, generalised estimation equations (GEEs) with interchangeable correlations will be used.

9. Power calculation

This study involves the follow-up of a historical clinical trial and the sample size is limited to the number of participants able to be located. For the primary outcome *composite of cardiovascular risk factors*, the sample size of 420 allows for detection of a 15% increase in the proportion with the outcome (relative risk 1.33) with 90% power ($\beta = 0.10$) at a significance level of 5% ($\alpha = 0.05$), assuming a baseline prevalence of 45% in the placebo group. For the primary outcome *Age at first major adverse cardiovascular event*, the sample size of 420 allows for detection of a hazard ratio of at least 0.33 with 90% power ($\beta = 0.10$) at a significance level of 5% ($\alpha = 0.05$), assuming a baseline prevalence of 8% in both the placebo and steroid groups.

10. Analysis based on Self-reported Outcomes

An aim of this research program is to compare outcomes based on expert clinical synthesis of administrative and self-reported data and those based on questionnaire responses completed by

each participant. A complementary analysis of the appropriate outcomes indicated in section 5 above by the superscript S (self-reported) will be completed with all participants for those questions which most closely match the outcomes listed in section 5.

11. Exploratory analyses

4.6. Enriched Sample Analysis

Where appropriate data from outcomes obtained at the 30-year follow up will be incorporated in the outcomes and the denominators corrected to include the number of participants in each treatment arm for whom data were available at either 30-year or 50-year follow up.

4.7. Subgroup analyses

Exploratory analyses for primary and secondary outcomes will investigate if the treatment effect of antenatal corticosteroid is influenced by the following variables:

- Trial protocol (standard-dose treatment compared with double-standard dose)
- Multiple pregnancy (singleton pregnancy compared with multiple pregnancy)
- Sex of the infant
- The tocolytic used (ethanol vs salbutamol vs none/unknown)
- The reason for preterm delivery
  Analyses for each of the outcomes listed will be performed with each of these potential subgroups as main and interaction effects (with treatment allocation) and measures of effect will be presented for each of the subgroup strata.

4.8. Sensitivity analyses

The following sensitivity analyses will be performed to examine the primary and secondary outcomes:

- Excluding participants for whom there were protocol deviations (per protocol analysis)
- Including current BMI, sex, current socioeconomic status, gestation at birth and birthweight z score (1) in the adjusted analysis

In addition, the following sensitivity analyses will be performed for diabetes outcomes

- Excluding participants for whom the reason for preterm birth was listed as maternal diabetes mellitus
- Excluding participants for whom there was a known family history of diabetes mellitus noted at 30 year follow-up

For the secondary outcome *Hierarchical win ratio outcome* a sensitivity analysis will be performed using time to death after 28 days, excluding those with fetal death or death before 28 days from the analysis.

4.9. Post hoc analyses

Any post-hoc, exploratory analyses which were not identified in this SAP but are completed to support the listed analyses will be identified clearly.

12. Descriptive analysis

A flow chart will be completed to describe participant flow from randomisation to 50 year follow-up.

Summary statistics will be presented for F1 participants included in the intention-to-treat analysis, with a comparison between characteristics of those followed up and those not followed up. Baseline demographic variables will be reported as mean and standard deviation or median and interquartile range where appropriate for continuous variables and as frequency and percentages for categorical variables.

13. Statistical Analysis

Statistical analysis will be performed for all primary, secondary and tertiary outcomes. Statistical significance will be tested at the 5% significance level using a two-sided test of significance. Analyses will compare the antenatal corticosteroid (betamethasone) group to the control (cortisone acetate) group.

Analyses for binary outcomes will be performed using log binomial regression analysis, reported as relative risk (RR) with 95% confidence intervals.

For continuous outcomes, analysis will be by general linear modelling after assessing model assumptions, using an identity link function and normal distribution.

For time-to-event analyses, a Cox proportional hazards model will be used, reported as a hazards ratio (HR) with 95% confidence intervals. The assumption of proportionality will be verified and if required time dependent functions modelled. Competing risk models will be used if appropriate.

For the hierarchical win-ratio outcome, analysis will follow a stepwise approach in which the six components of the composite outcomes are assessed sequentially (2).The six steps in the hierarchy will start with time to death after randomisation, followed by time to major adverse cardiovascular event, diagnosis of diabetes mellitus, number of admissions to hospital for respiratory illness, self-reported general health and time spent in hospital per 10 years alive. For each step, participants in the treatment and control group will be compared in all possible pairs to determine “wins”, “losses” and “ties”. Pairwise comparisons with ties or missing data at any step will be assessed at the next sequential step in the hierarchy. The analysis will then determine the ratio of the probability of wins and losses for the treatment. The resulting odds ratio will be reported with 95% confidence intervals (2).

14. Appendix

12.1 Flow diagram

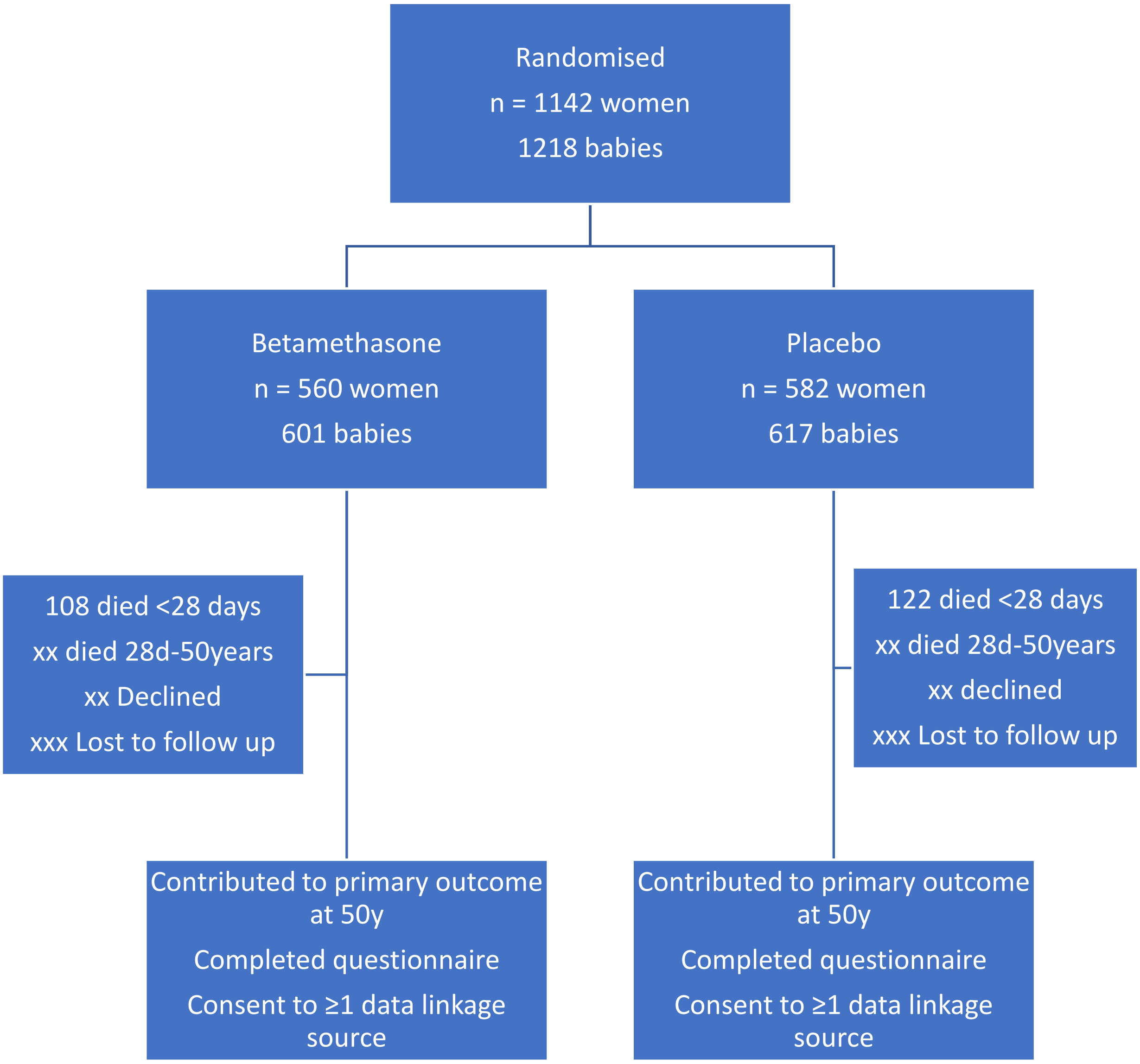

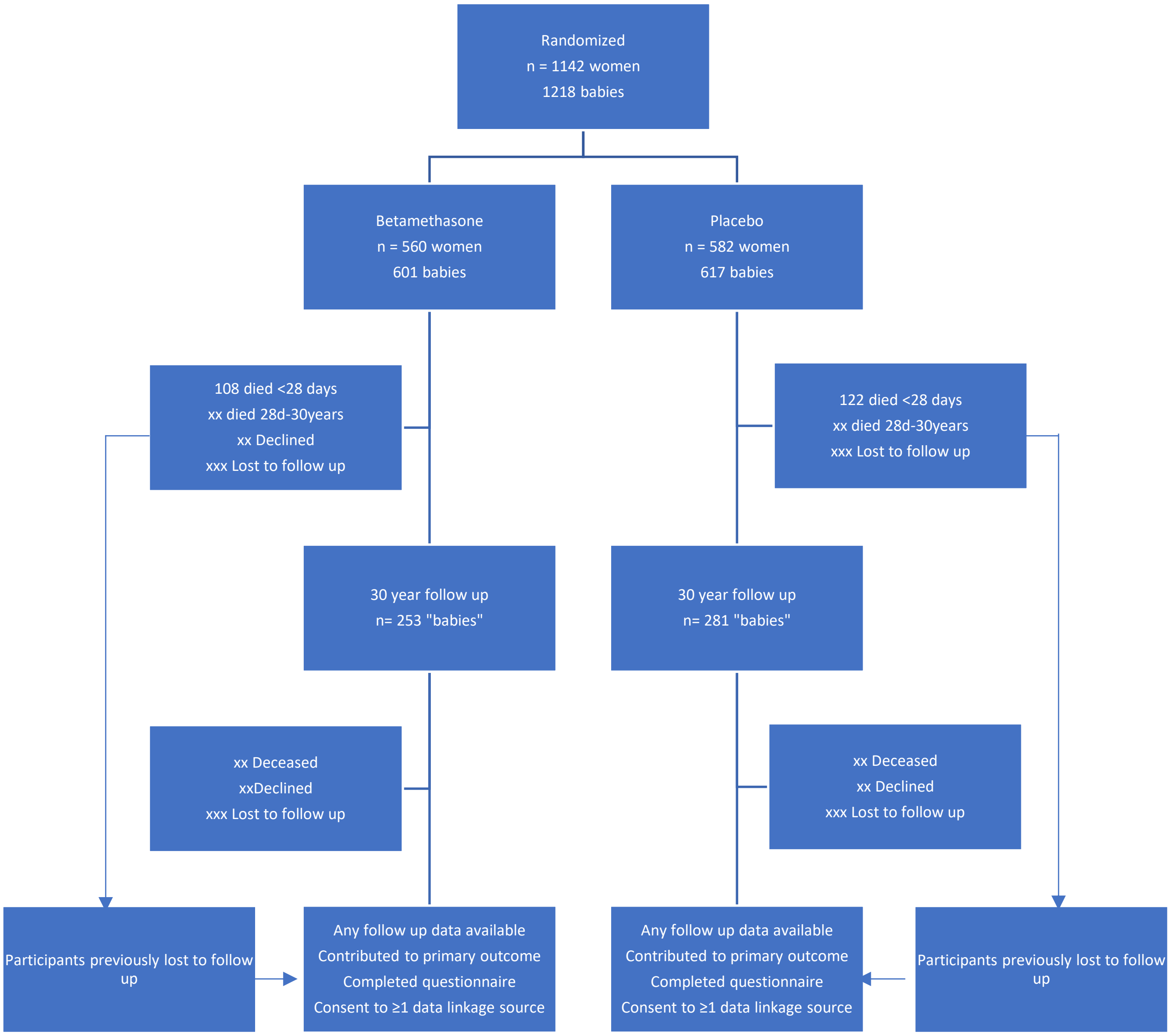
Randomized
n = 1142 women
1218 babies
Betamethasone
n = 560 women
601 babies
Placebo
n = 582 women
617 babies
108 died <28 days
xx died 28d-30years
xx Declined
xxx Lost to follow up
122 died <28 days
xx died 28d-30years
xxx Lost to follow up
30 year follow up
n= 253 "babies"
30 year follow up
n= 281 "babies"
xx Deceased
xxDeclined
xxx Lost to follow up
xx Deceased
xx Declined
xxx Lost to follow up
Participants previously lost to follow up
Any follow up data available
Contributed to primary outcome
Completed questionnaire
Consent to ≥1 data linkage source
Any follow up data available
Contributed to primary outcome
Completed questionnaire
Consent to ≥1 data linkage source
Participants previously lost to follow up

12.2 Draft Tables

Table 1 Baseline characteristics of those eligible who did and did not participate

	**Participated**	**Did not participate**
Female (%)		
Gestational age at entry, weeks (mean, SD)		
Gestational age at delivery (mean, SD)		
Multiple pregnancy (%)		
Singleton		
Multiple		
Unplanned premature labour (%)		
Planned delivery (%)		
Hypertension-oedema-proteinuria syndromes		
Rh isoimmunisation		
Placenta praevia		
Diabetes mellitus		
Other		
Mode of delivery (%)		
Normal vaginal delivery		
Instrumental delivery		
Caesarean section		
Tocolytic used (%)		
Ethanol		
Salbutamol		
Both		
Treatment dose of betamethasone or placebo (%)		
Standard dose		
Twice standard dose		
Term delivery (%)		
Birthweight, g (mean, SD)		
Birthweight Z score (mean, SD)		
5 minute Apgar score <7 (%)		

Respiratory distress syndrome		

Table 2 Baseline characteristics of participants whose mothers were randomised to betamethasone and placebo

	**Betamethasone**	**Placebo**
**Maternal Characteristics**		
Gestational age at entry, days (mean, SD)		
Gestational age at delivery, days (mean, SD		
Multiple pregnancy (%)<br>Singleton<br>Multiple		
Unplanned premature labour (%)		
Planned delivery (%)<br>Hypertension-oedema-proteinuria syndromes<br>Rh isoimmunisation<br>Placenta praevia<br>Diabetes mellitus<br>Other		
Mode of delivery (%)<br>Normal vaginal delivery<br>Instrumental delivery<br>Caesarean section		
Tocolytic used (%)<br>Ethanol<br>Salbutamol<br>Both		
Treatment dose of betamethasone or placebo (%)<br>Standard dose<br>Twice standard dose		
Protocol deviations (%)		
**Neonatal Characteristics**		
Female (%)		

Term delivery (%)		
Birthweight, g (mean, SD)		
Birthweight Z score (mean, SD)		
5 minute Apgar score <7 (%)		
Respiratory distress syndrome (%)		
**Adult Characteristics**		
Age at follow up, years (mean, SD)		
Ethnicity (%)<br>Māori<br>New Zealand European<br>Pacific<br>Other		
Smoking status (%)<br>Non-smoker<br>Previously smoked<br>Currently smokes		

Table 3 Primary outcomes

	Betamethasone N =	Placebo N =	Unadjusted RR or HR (95% CI)	Adjusted RR or HR (95% CI)	P value
**Cardiometabolic risk factor composite (%)[1]**					
**Time to first major adverse cardiovascular event (median, 95% CI)[2]**					

[1]Relative risk, [2]Hazard ratio

Table 4 Secondary outcomes

Outcome	Betamethasone N =	Placebo N =	Unadjusted RR, HR, OR	Adjusted RR, HR or mean	P value

			or mean difference (95% CI)	difference (95% CI)	
Diabetes mellitus or prediabetes (n/N, %)[1]					
Diabetes mellitus (n/N, %)[1]					
Prediabetes (n/N, %)[1]					
Hypertension (n/N, %)[1]					
Dyslipidaemia (n/N, %)[1]					
Age at cardiovascular death (median, 95% CI)[2]					
Proportion with cardiovascular death (n/N, %)[1]					
Age at first admission for myocardial infarction or coronary revascularisation (median, 95% CI)[2]					
Age at first admission for myocardial infarction (median, 95% CI)[2]					
Age at first admission for coronary revascularisation (median, 95% CI)[2]					
Age at first admission for peripheral vascular disease (median, 95% CI)[2]					
Age at first admission for arterial thrombosis/embolism (median, 95% CI)[2]					
Age at first admission for peripheral revascularisation procedure (median, 95% CI)[2]					
Age at first admission for stroke (median, 95% CI)[2]					
Age at first heart failure admission (median, 95% CI)[2]					
Proportion with at least one admission for myocardial infarction or coronary revascularisation (n/N, %)[1]					
Proportion with at least one admission for myocardial infarction (n/N, %)[1]					
Proportion with at least one admission for coronary revascularisation (n/N, %)[1]					

Proportion with at least one admission for peripheral vascular disease (arterial thrombosis/embolism or need for revascularisation) (n/N, %)[1]					
Proportion with at least one admission for arterial thrombosis/embolism (n/N, %)[1]					
Proportion with at least one admission for peripheral revascularisation procedure (n/N, %)[1]					
Proportion with at least one admission for stroke (n/N, %)[1]					
Proportion with at least one admission for heart failure (n/N, %)[1]					
Win-odds Hierarchical outcome[4]					
Time to death after randomisation (median, 95% CI)[4]					
Time to MACE (excluding cardiovascular death) (median, 95% CI)[4]					
Diagnosis of diabetes mellitus (excluding prediabetes) (n/N, %)[4]					
Number of admissions to hospital with respiratory illness as primary reason for admission (admissions per patient year) (mean, SD)[4]					
Self-reported general health (mean, SD)[4]					
Time in Hospital per 10 years alive after 1988 (median, 95% CI)[4]					
Ischaemic heart disease (n/N, %)[1]					
Stroke (n/N, %)[1]					
Peripheral vascular disease (n/N, %)[1]					
Overweight or obesity (n/N, %)[1]					
BMI (continuous) (mean, SD)[3]					
Death from any cause (n/N, %)[1]					
Time to death after randomisation (mean, SD)[3]					
Time in Hospital per 10 years alive after 1988 (mean, SD)[3]					

Self-reported general health (mean, SD)[3]					
Rate of admissions to hospital with respiratory illness as the primary reason for admission/per patient year of followup (mean, SD)[3]					
Chronic respiratory illness (Self-reported diagnosis of asthma, self-reported chronic respiratory symptoms, admissions for asthma or chronic obstructive pulmonary disease [COPD] or prescription of pharmaceuticals for asthma or COPD) c					
Asthma (n/N, %)[1]					
Chronic respiratory symptoms (n/N, %)[1]					
Proportion with at least one admission for asthma or chronic obstructive pulmonary disease (COPD) (n/N, %)[1]					
Prescriptions of pharmaceuticals for asthma or COPD (n/N, %)[1]					
Highest level of educational attainment (n/N, %)[1]<br>Tertiary<br>Secondary<br>Neither tertiary nor secondary					

[1]Relative risk, [2]Hazard ratio, [3]Mean difference, [4]Hierarchical win ratio outcome: 6 step, unmatched, win ratio hierarchy including time to death after randomisation; time to first major adverse cardiovascular event (MACE); diagnosis of diabetes mellitus; number of admissions to hospital with respiratory illness as primary reason for admission; self-reported general health; time in hospital per 10 years alive after 1988

Table 5 Tertiary outcomes

**Outcome**	**Betamethasone N =**	**Placebo N =**	**Unadjusted RR or mean difference (95% CI)**	**Adjusted RR or mean difference (95% CI)**	**P value**
Functional difficulties (n/N, %)[1]					
Physical activity (categorical) (n/N, %)[1]					

Type 2 diabetes mellitus (n/N, %)[1]					
Type 1 diabetes mellitus (n/N, %)[1]					
Gestational diabetes mellitus (n/N, %)[1]					
Heart failure (n/N, %)[1]					
Atrial fibrillation (n/N, %)[1]					
Number of babies ≥20 weeks' gestation fathered (median, 95% CI)[3]					
Age at menarche (mean, SD)[3]					
Proportion who have reached menopause (n/N, %)[1]					
Age at menopause (mean, SD)[3]					
Need for assisted reproductive technology (n/N, %)[1]					
Polycystic ovarian syndrome (n/N, %)[1]					
Total number of pregnancies ≥20 weeks (parity) (median, 95% CI)[3]					
Diagnosis or treatment of a mental health disorder (n/N, %)[1]					
Depression (n/N, %)[1]					
Bipolar affective disorder (n/N, %)[1]					
Anxiety disorder (n/N, %)[1]					
Schizophrenia (n/N, %)[1]					
Inpatient admission for a mental health disorder (excluding mental disorders due to known physiological conditions) (n/N, %)[1]					
Prescriptions of pharmaceuticals for mental health disorders (n/N, %)[1]					
Total number of fractures (median, 95% CI)[3]					
Cancer diagnosis (n/N, %)[1]					
Allergic condition other than asthma (diagnosis of or prescription of treatments for allergic conditions) (n/N, %)[1]					
Chronic kidney disease (n/N, %)[1]					
Self-reported oral health (n/N, %)[1]					

Number of teeth removed for decay (median, 95% CI)[3]					
Any court charges or convictions (n/N, %)[1]					
Employment status (n/N, %)[1]					
Proportion with tertiary qualifications (n/N, %)[1]					
No secondary school qualification (n/N, %)[1]					

[1]Relative risk, [2]Hazard ratio, [3]Mean difference

Table 6 Subgroup analyses for primary outcomes

Outcome and sub-group	Betamethasone N =	Placebo N =	Unadjusted RR or HR (95% CI)	Adjusted RR or HR (95% CI)	P value	Interaction P value
**Cardiometabolic risk factor composite**						
**Trial protocol**						
Standard-dose treatment						
Double-standard dose treatment						
**Multiple Pregnancy**						
Singleton						
Multiple						
**Infant sex**						
Female						
Male						
**Tocolytic used**						
Ethanol						
Salbutamol						
Both						
None						
**Reason for preterm birth**						
Unplanned preterm birth						
Hypertensive disorders of pregnancy						
Rh iso-immunisation						
Placenta praevia						
Diabetes mellitus						

**Time to first major adverse cardiovascular event**						
**Trial protocol**						
Standard-dose treatment						
Double-standard dose treatment						
**Multiple Pregnancy**						
Singleton						
Multiple						
**Infant sex**						
Female						
Male						
**Tocolytic used**						
Ethanol						
Salbutamol						
Both						
None						
**Reason for preterm birth**						
Unplanned preterm birth						
Hypertensive disorders of pregnancy						
Rh iso-immunisation						
Placenta praevia						
Diabetes mellitus						

[1]***Relative risk,*** [2]***Hazard ratio***

Table 7 Subgroup analyses for secondary outcomes

**Outcome and sub-group**	**Betamethasone N =**	**Placebo N =**	**Unadjusted RR, HR or mean difference (95% CI)**	**Adjusted RR, HR or mean difference (95% CI)**	**P value**	**Interaction**
**Secondary outcome**						
**Trial protocol**						
Standard-dose treatment						

Double-standard dose treatment						
**Multiple Pregnancy**						
Singleton						
Multiple						
**Infant sex**						
Female						
Male						
**Tocolytic used**						
Ethanol						
Salbutamol						
Both						
None						
**Reason for preterm birth**						
Unplanned preterm birth						
Hypertensive disorders of pregnancy						
Rh iso-immunisation						
Placenta praevia						
Diabetes mellitus						

[1]Relative risk, [2]Hazard ratio, [3]Mean difference

Table 8 Sensitivity analyses

Primary or secondary outcome	Betamethasone N =	Placebo N =	Unadjusted RR, HR or mean difference (95% CI)	Adjusted RR, HR or mean difference (95% CI)	P value
**Using questionnaire data only**					
**Using per protocol analysis**					
**Including additional covariates in the statistical model[4]**					

**Excluding participants for whom the reason for preterm birth was listed as maternal diabetes mellitus**					
Diabetes mellitus (any type)					
Type 2 diabetes mellitus					
Type 1 diabetes mellitus					
Gestational diabetes mellitus					
**Excluding participants for whom there was a known family history of diabetes mellitus noted at 30 year follow-up**					
Diabetes mellitus (any type)					
Type 2 diabetes mellitus					
Type 1 diabetes mellitus					
Gestational diabetes mellitus					

[1]Relative risk, [2]Hazard ratio, [3]Mean difference,[4] Adjusted for current BMI, sex, current socioeconomic status, gestation at birth and birthweight z score in the adjusted analysis

Table 9 Sensitivity analysis for win-odds hierarchical outcome analysis excluding those with fetal death or death before 28 days

**Outcome**	**Betamethasone N =**	**Placebo N =**	**Odds Ratio (95% CI)**	**P value**

Win-odds Hierarchical outcome				

<u>**Supplementary Tables**</u>

Supplementary Table 1 Primary outcomes based on self-reported questionnaire only

**Outcome**	**Betamethasone N =**	**Placebo N =**	**Unadjusted RR, HR or mean difference (95% CI)**	**RR, HR or mean difference (95% CI)**	**P value**

Similar tables will be completed for the complementary analysis of self-reported outcomes

15. References

1. Thompson JM, Mitchell EA, Borman B. Sex specific birthweight percentiles by gestational age for New Zealand. N Z Med J. 1994 Jan 26;107(970):1–3.

2. Redfors B, Gregson J, Crowley A, McAndrew T, Ben-Yehuda O, Stone GW, et al. The win ratio approach for composite endpoints: practical guidance based on previous experience. Eur Heart J. 2020 Dec 7;41(46):4391–9.

Addendum to ANCHOR_AST_Statistical Analysis PlanV1.8_26August2022Final
Date: 21 October 2022

Addendum to statistical analysis plan for Auckland Steroid Trial Follow up

The following text will replace section 4.7 for the approach to clustering:

Inclusion of infants from multiple (twin or triplet) pregnancies in the analysis is a potential source of clustering, as is women having more than one pregnancy included in the study. To account for the effect of clustering of infants from the same mother, random effects model will be used.

The following bullet point will be added to the first list in section 4.8 for sensitivity analyses:

- Excluding the second child in cases of women having more than one pregnancy within the study.

Addendum to ANCHOR_AST_Statistical Analysis PlanV1.8_26August2022Final
Date: 15 December 2022

Addendum to statistical analysis plan for Auckland Steroid Trial Follow up

The following text will be added to section 5.1.1 (Composite of cardiovascular risk factors. Any of:) and 5.2 (Components of the Primary outcomes):

- Gestational diabetes mellitus
