# Supplementary material for: Cardiovascular outcomes 50 years after antenatal exposure to betamethasone: Follow-up of a randomised double-blind, placebo-controlled trial
Source: PLoS Med. 2024 Apr 1;21(4):e1004378. doi: 10.1371/journal.pmed.1004378 (PMC11018286; doi:10.1371/journal.pmed.1004378)
Supplement: S1 Appendix — (DOCX) [file pmed.1004378.s003.docx]

**Auckland Steroid Trial 50-year follow up**

Supplement to statistical analysis plan:

Outcome Definitions

Version 2.0, 30 January 2023

Author: Anthony Walters

| Contents  [Outcomes list 3](#_Toc138924205)  [Primary outcomes 6](#_Toc138924206)  [Secondary outcomes 7](#_Toc138924207)  [Cardiovascular risk factors 7](#_Toc138924208)  [Cardiovascular disease outcomes 13](#_Toc138924209)  [General Health Outcomes 22](#_Toc138924210)  [Respiratory disease 24](#_Toc138924211)  [Education 25](#_Toc138924212)  [Tertiary Outcomes 26](#_Toc138924213)  [General Health 26](#_Toc138924214)  [Diabetes mellitus 28](#_Toc138924215)  [Cardiovascular 30](#_Toc138924216)  [Respiratory 31](#_Toc138924217)  [Reproductive System 35](#_Toc138924218)  [Mental Health 37](#_Toc138924219)  [Other Health Outcomes 43](#_Toc138924220)  [Social Outcomes 47](#_Toc138924221) Outcomes list | | | | |
| --- | --- | --- | --- | --- |
| **Primary Outcomes:**   1. Composite of cardiovascular risk factors. 2. Age at first major adverse cardiovascular event | | | | |
| **Secondary outcomes:**  **Components of the Primary outcomes**   - Diabetes mellitus or prediabetes^c^ - Diabetes mellitus^c^ - Prediabetes^c^ - Hypertension^c^ - Dyslipidemia^c^ - Age at cardiovascular death^b^ - Proportion with cardiovascular death^b^ - Age at first admission for myocardial infarction or coronary revascularization^b^ - Age at first admission for myocardial infarction^b^ - Age at first admission for coronary revascularization^b^ - Age at first admission for peripheral vascular disease (arterial thrombosis/embolism or need for revascularization)^b^ - Age at first admission for arterial thrombosis/embolism^b^ - Age at first admission for peripheral revascularization procedure^b^ - Age at first admission for stroke^b^ - Age at first heart failure admission^b^ - Proportion with at least one admission for myocardial infarction or coronary revascularization^b^ - Proportion with at least one admission for myocardial infarction^b^ - Proportion with at least one admission for coronary revascularization^b^ - Proportion with at least one admission for peripheral vascular disease (arterial thrombosis/embolism or need for revascularization)^b^ - Proportion with at least one admission for arterial thrombosis/embolism^b^ - Proportion with at least one admission for peripheral revascularization procedure^b^ - Proportion with at least one admission for stroke^b^ - Proportion with at least one admission for heart failure^b^   **Win Ratio Secondary Outcomes**   - Hierarchical win ratio outcome^a^: 6 step, unmatched, win ratio hierarchy including time to death after randomization; time to first major adverse cardiovascular event; diagnosis of diabetes mellitus; number of admissions to hospital with respiratory illness as primary reason for admission; self-reported general health; time in hospital per 10 years alive after 1988 - Components of the win ratio outcome   - Time to death after randomization^a^   - Time to MACE (excluding cardiovascular death)^b^   - Diagnosis of diabetes mellitus (excluding prediabetes)^c^   - Number of admissions to hospital with respiratory illness as primary reason for admission (admissions per patient year)^b^   - Self-reported general health^c^   - Time in Hospital per 10 years alive after 1988^b^   **Additional Secondary Outcomes**   - Ischemic heart disease^c^ - Stroke^c^ - Peripheral vascular disease^c^ - Overweight or obesity^c^ - BMI (continuous)^c^ - Death from any cause^a^ - Time to death after randomization^a^ - Time in Hospital per 10 years alive after 1988^b^ - Self-reported general health^c^ - Rate of admissions to hospital with respiratory illness as the primary reason for admission/per patient year of followup^c^ - Asthma or chronic obstructive pulmonary disease (Self-reported diagnosis of asthma, self-reported chronic respiratory symptoms, admissions for asthma or chronic obstructive pulmonary disease [COPD] or dispensing of pharmaceuticals for asthma or COPD) ^c^   - Asthma^c^   - Chronic respiratory symptoms^c^   - Proportion with at least one admission for asthma or chronic obstructive pulmonary disease (COPD) ^c^   - Dispensing of pharmaceuticals for asthma or COPD^c^ | | | | |
| ^a^ Denominator will be all participants whose mothers were randomized in the original trial.  ^b^ Denominator will be all participants alive at 28 days of age.  ^c^ Denominator will be all participants who consented to this follow-up study with data available for the outcome.  **Abbreviations:**  **IPAG: International primary care airways group; MORT: Ministry of health mortality data collection; NMDS: National Minimum Dataset; NNPAC National Non-admitted patients collection; NZSSD: New Zealand Society for the Study of Diabetes; NZQA: New Zealand Qualifications Authority; PHARMS: New Zealand Pharmaceuticals collection; VDR: Virtual diabetes register.** | | | | |
| **Study Outcome (definitions)** | **Priority Order for use of Data Sources** | **Details of data:** | **Criteria for adjudication (if applicable)** | **Comments:** |
| Primary outcomes | | | | |
| **Composite of cardiovascular risk factors.**  **Any of:**   1. **Diabetes mellitus or prediabetes or gestational diabetes mellitus** 2. **Hypertension** 3. **Dyslipidemia** | See below under section for cardiovascular risk factors | Composite outcome will be met if a participant has one or more of the conditions listed. |  |  |
| **Age at first major adverse cardiovascular events.**  **Earliest of :**   1. **Age at cardiovascular death** 2. **Age at first admission for ischemic heart disease (myocardial infarction, angina, coronary revascularization)** 3. **Age at first admission for peripheral vascular disease events (arterial thrombosis/embolism or need for revascularization)** 4. **Age at first admission for stroke** 5. **Age at first admission for heart failure** | See below under section for cardiovascular and mortality outcomes | Composite outcome will be met if a participant has one or more of the conditions listed. |  |  |
| Secondary outcomes | | | | |
| Cardiovascular risk factors | | | | |
| **Diabetes mellitus or prediabetes:**  **Either of : “any diabetes mellitus” or “prediabetes”** | 1. As defined below |  |  |  |
| **Any diabetes mellitus**  Defined as presence of any of the following:   1. Self-report of diagnosis of diabetes   OR two of the following   1. Hospital admission with a diagnosis code of diabetes mellitus 2. Dispensing of pharmaceuticals for treatment of diabetes mellitus 3. Two of: HbA1c ≥ 50 mmol/mol or fasting glucose ≥ 7 mmol/L or random glucose ≥ 11.1 mmol/L or 2 hour OGTT glucose ≥ 11.1 mmol/L 4. Outpatient interaction with retinal screening, diabetes education or diabetes outpatient specialist appointment | 1. Self report Questionnaire Q. B18-B21 | Told by doctor you have diabetes – excluding GDM (Q.B18)  Type of diabetes you have been told you have (Type 1, Type 2) (Q.B19)  Age at diagnosis (Q.B20)  Treatment type (No treatment, insulin, medicines, tablets, pills, diet, exercise, other) (Q.B21) | If answers “No” to question about diabetes mellitus but has 2 or more other data sources suggesting diabetes mellitus then classify as DM. If answers no to question about DM but has only one piece of information then case will be adjudicated using available information.  If answers “yes” but no diabetes treatment (self-report or NZ pharmaceuticals collection), still considered as having as diabetes. | **New Zealand Society for the Study of Diabetes 2021 definition of diabetes mellitus** [1].  If symptomatic diagnosed by:  1.HbA1c ≥ 50 mmol/mol, or  2. Fasting glucose ≥ 7 mmol/L or  3. Random glucose ≥ 11.1 mmol/L.  If asymptomatic diagnosed by  1. Two abnormal tests of [HbA1c (≥ 50 mmol/mol), or fasting glucose ≥ 7 mmol/L, or random glucose ≥ 11.1 mmol/L] either on same day or subsequent test without delay.  2. If 75 gram oral glucose tolerance test  Fasting glucose ≥ 7 mmol/L or 2-hour glucose is ≥ 11.1 mmol/L. |
|  | 1. NMDS | Hospital admissions with a diagnosis of diabetes mellitus. This includes the following ICD 10-AM codes:  E10 (Type 1 diabetes mellitus), E11 (Type 2 diabetes mellitus), E13 (Other diabetes mellitus), E14 (diabetes mellitus unspecified), O24.0 (pre-existing Type 1 diabetes mellitus during pregnancy), O24.1 (pre-existing type 2 diabetes mellitus), O24.2 (pre-existing malnutrition associated diabetes mellitus in pregnancy), O24.3 (pre-existing diabetes mellitus unspecified in pregnancy). |  |  |
|  | 1. N.Z pharmaceutical collection | Dispensing for any of the following*  Metformin/vildagliptin (ie. Galvumet or Galvus (vildagliptin))  Insulin (rapid-acting: NovoRapid, Apidra, Apidra Solostar, Humalog)  (short-acting: Actrapid, Humulin R)  (intermediate and long-acting: isophane/Protophane, Humulin NPH, Lantus, Lantus Solostar, detemeir/Levemir)  Suphonylureas (gliclazide, glibenclamide, glipizide)  Acarbose  Pioglitazone  Empagliflozin (Jardiance) or Jardiamet  (Empagliflozin/metformin)  Dapagliflozin  Dulaglutide | If dispensed metformin and is male then classify as diabetes mellitus. If dispensed metformin, is female and answers “no” to polycystic ovarian syndrome (PCOS) in questionnaire then classify as diabetes mellitus. If “yes” or did not answer PCOS question then needs additional information to diagnose diabetes mellitus. |  |
|  | 1. Testsafe lab results | HbA1c results + dates  Glucose tests + dates  GTT results and tests |  |  |
|  | 1. NNAPC | Purchase unit codes M20006 (diabetes education and care) and M200007 (diabetes fundus screening), MAOR0106 Diabetes management-Maori, M20004 Diabetes 1st attendance, M20005 Diabetes subsequent attendance, M20010 High risk type 1 diabetes support, M20011 outreach diabetes services, M20015 High risk type 1 diabetes support for up to 18 year olds, COOC0012 diabetes nurse educator, COPG0013 management services diabetes |  |  |
| **Prediabetes**  Defined as presence of any of the following, in the absence of a diagnosis of diabetes mellitus:  1. Self-report of prediabetes  2. HbA1c 41 – 49 mmol/mol or  3. Fasting glucose 6.1 – 6.9 mmol/L or  4. 2 hour glucose on GTT 7.8 – 11 mmol/L. | 1. Self report  Questionnaire  Q.B18-B21 | Told by doctor you have diabetes– excluding GDM (Q.B18)  Type of diabetes you have been told you have (Pre-diabetes) (Q.B19)  Age at diagnosis (Q.B20)  Treatment type (No treatment, insulin, medicines, tablets, pills, diet, exercise, other) (Q.B21) | If answers “no” to diabetes/prediabetes question but has biochemical evidence of prediabetes then case will be adjudicated. |  |
|  | 2. Testsafe lab results | HbA1c results + dates  Glucose tests + dates  GTT results and tests | If HbA1c 41-49 check no medications (oral hypoglycemics) from (self-report or NZ pharmaceuticals collection) to exclude well controlled diabetes. | **New Zealand Society for the Study of Diabetes 2021 definition of prediabetes** [1]:  Diagnosed if:  1. HbA1c 41 – 49 mmol/mol or  2. Fasting glucose 6.1 – 6.9 mmol/L or  3. 2 hour glucose on GTT 7.8 – 11 mmol/L. |
|  | 3. If self report but does not know type of diabetes Q.B18) | Check treatment type (No treatment, insulin, medicines, tablets, pills, diet, exercise, other) (Q.B21). | If on no treatment (self-report or NZ pharmaceuticals collection) assume pre-diabetes. |  |
| **Gestational diabetes mellitus (GDM)**  **Either of:**   1. **Self-report of a diagnosis of GDM** 2. **Admissions to hospital with a diagnostic code for GDM** | Self- report questionnaire (Q.C11-12) | During your pregnancy/ies did a doctor ever tell you that you had developed gestational diabetes? (Q.C11) What treatment did you receive for the diabetes you developed when you were pregnant? (No treatment, insulin, medicines, tablets, pills, diet, exercise, other) (Q.C12) | If reports “no” to a diabetes diagnosis but has laboratory evidence or hyperglycemia or treatment of hyperglycemia then will be adjudicated along with possible prediabetes cases. |  |
|  | NMDS | Hospital admissions with a diagnosis of gestational diabetes mellitus. This includes the following ICD 10 codes:  O24.4 (Diabetes mellitus arising during pregnancy). |  |  |
| **Hypertension**  Defined as presence of any of the following:   1. Self-report of diagnosis of high blood pressure 2. Dispensing of a listed pharmaceutical for treatment of hypertension | 1. Self-report Questionnaire | Told by doctor you have high BP (Q.B1)  Currently taking medication for high BP (Q.B2) | If responds no to high blood pressure question but has evidence of hypertension in NMDS or Pharms datasets then will be adjudicated. |  |
|  | 1. NZ pharmaceuticals collection | Prescriptions + dates  Angiotensin converting enzyme (ACE) inhibitors  (ie. Captopril, Cilazapril, Enalapril, Lisinopril, Perindopril, quinapril)  Angiotensin receptor blockers (ARBs) (ie. Candesarten, Losarten, Irbesarten)  Beta blockers (Atenolol, bisoprolol, carbedilol, celiprolol, labetalol, metoprolol, propranolol, sotalol)  Diuretics (hydrochlorothiazide, bendroflumathiazide, chlorthalidone, indapamide)  Calcium channel blockers (amlodipine, diltiazem, felodipine, nifedipine)  Combination (inhibace plus, accuretic, Arrow-Losartan & Hydrochlorothiazide) |  |  |
|  | 1. NMDS | Hospital admission with a diagnosis code of hypertension: I10 Essential hypertension; I110, I119 hypertensive heart disease; I120, I129 Hypertensive renal disease; I13x Hypertensive heart and renal disease, I15x secondary hypertension |  |  |
| **Hyperlipidemia/High Cholesterol**  Defined as presence of any of the following:   1. Self-report of diagnosis of high cholesterol 2. Testsafe lab results of hypercholesterolemia or hypertriglyceridemia 3. Dispensing of a listed pharmaceutical for treatment of dyslipidemia | 1. Self report Questionnaire | Told by a doctor that you have high cholesterol (Q.B3)  Currently taking pills for high cholesterol (Q.B4) | If responds no to high cholesterol question but has evidence in Testsafe or Pharms datasets then will be adjudicated. |  |
|  | 1. Testsafe lab results | Lipid profile tests and dates |  | Hyperlipidemia, either hypercholesterolemia or hypertriglyceridemia: if TC >5 mmol/L LDL ≥3.4 mmol/L, or Triglycerides ≥2 mmol/L based on cardiovascular disease risk enhancers [2]. |
|  | 1. NZ pharmaceuticals collection | Prescriptions + dates  Statins (Atorvastatin, Pravastatin, Rosuvastatin, Simvastatin)  Fibrate (bezafibrate)  Niacin derivatives (acipimox)  Ezetimibe  Colestipol |  |  |
| **Obesity/Overweight**  **Proportion with BMI >25 (Overweight); >30 (Obese); Obesity Class 1(30 to <35), ,2(35 to <40) and 3(≥40)** | Classified using BMI based on self-reported height and weight. | What is your current weight? (Q.F2)  What is your current height (Q.F3) |  |  |
| **BMI**  Continuous | Calculated from Self-reported Questionnaire information | What is your current weight? (Q.F2)  What is your current height (Q.F3) |  |  |
| Cardiovascular disease outcomes | | | | |
| **Cardiovascular death**  Primary cause of death is from a cardiovascular cause (myocardial infarction, stroke, revascularization procedure, arrhythmia, sudden death, death due to heart failure, death due to cardiovascular hemorrhage, death due to other cardiovascular causes) [3].  Age at cardiovascular death | MORT dataset/NMDS (primary cause of death matches ICD-10 AM codes) | **ICD-10 AM codes:**  I210-I214, I219: Acute myocardial infarction  I220-I221, I228-I229: Subsequent myocardial infarction  I230-I236, I238: Complications of acute myocardial infarction  I240, I248, I249 Other acute ischemic heart diseases  G460-468: Vascular syndromes of brain in cerebrovascular diseases  I600-I609: Subarachnoid hemorrhage  I610-I616, I618, I619: Intracerebral hemorrhage  I630-I636, I638,I639: Cerebral infarction  I64: Stroke, not specified as hemorrhage or infarction  I690, I691, I693, I694, I698: Sequelae of subarachnoid hemorrhage, Sequelae of intracerebral hemorrhage, Sequelae of cerebral infarction, Sequelae of stroke, not specified as hemorrhage or infarction, Sequelae of other and unspecified cerebrovascular diseases  I462: Cardiac arrest due to underlying cardiac conditions  I7100-I7103, I711, I713-I715, I718: Aortic aneurysm and dissection  I110 Hypertensive heart disease with heart failure  I130 Hypertensive heart and chronic kidney disease with heart failure and stage 1 through stage 4 chronic kidney disease, or unspecified chronic kidney disease  I132 Hypertensive heart and chronic kidney disease with heart failure and with stage 5 chronic kidney disease, or end stage renal disease  I50, I500-I501, I509: Heart failure, congestive heart failure, Left ventricular failure, heart failure unspecified  I255: Ischemic cardiomyopathy  I461: Sudden cardiac death |  |  |
| **Ischemic heart disease**  Defined by self-report of diagnosis or hospitalizations with diagnostic codes for myocardial infarction, angina, coronary revascularization (surgical or percutaneous) | 1. Self- report questionnaire (Q.B5-B11) | Told by doctor you have had a heart attack (Q.B5)  Age at time of first heart attack (QB6)  Admitted to hospital with heart attack (Q.B7)  Was this within the last 12m? (Q.B8)  Told by doctor you have angina (Q.B9)  Bypass surgery or angioplasty for your heart condition (Q.B10)  Age at time this happened (Q.B11) |  |  |
|  | 2. National Minimum Dataset (NMDS)  (hospitalizations with discharge codes) | **ICD-10AM codes**  I210-I214, I219: Acute myocardial infarction  I220-I221, I228-I229: Subsequent myocardial infarction  Z951, Z955: Presence of aorto-coronary bypass graft, Presence of coronary angioplasty implant and graft  3849700- 3849707, 3850000- 3850004, 3850300- 3850304, 9020100- 9020103: Coronary artery bypass procedures.  3530400- 3530401, 3530500- 3530501, 3531000- 3531005: Coronary artery angioplasty |  |  |
| **Myocardial Infarction**  Self-report of myocardial infarction diagnosis or hospitalization with diagnostic codes for myocardial infarction | Self- report questionnaire (Q.B5-B11) | Told by doctor you have had a heart attack (Q.B5)  Age at time of first heart attack (QB6)  Admitted to hospital with heart attack (Q.B7)  Was this within the last 12m? (Q.B8)  Told by doctor you have angina (Q.B9)  Bypass surgery or angioplasty for your heart condition (Q.B10)  Age at time this happened (Q.B11) |  |  |
|  | National Minimum Dataset (NMDS)  (hospitalizations with discharge codes) | **ICD-10-AM codes**:  I210-I214, I219: Acute myocardial infarction  I220-I221, I228-I229: Subsequent myocardial infarction |  |  |
| **Myocardial Infarction**  At least one admission for MI.  Age at first admission for MI. | National Minimum Dataset (NMDS)  (hospitalizations with discharge codes) | **ICD-10-AM codes**:  I210-I214, I219: Acute myocardial infarction  I220-I221, I228-I229: Subsequent myocardial infarction |  |  |
| **Coronary revascularization procedure** | Self- report questionnaire (Q.B5-B11) | Told by doctor you have had a heart attack (Q.B5)  Age at time of first heart attack (QB6)  Admitted to hospital with heart attack (Q.B7)  Was this within the last 12m? (Q.B8)  Told by doctor you have angina (Q.B9)  Bypass surgery or angioplasty for your heart condition (Q.B10)  Age at time this happened (Q.B11) |  |  |
|  | National Minimum Dataset (NMDS)  (hospitalizations with discharge codes) | **ICD-10-AM codes**:  3849700- 3849707, 3850000- 3850004, 3850300- 3850304, 9020100- 9020103: Coronary artery bypass procedures.  3530400- 3530401, 3530500- 3530501, 3531000- 3531005: Coronary artery angioplasty |  |  |
| **Admission for coronary revascularization procedure**  At least one admission for revascularization.  Age at first admission for revascularization. | National Minimum Dataset (NMDS)  (hospitalizations with discharge codes) | **ICD-10-AM codes**:  3849700- 3849707, 3850000- 3850004, 3850300- 3850304, 9020100- 9020103: Coronary artery bypass procedures.  3530400- 3530401, 3530500- 3530501, 3531000- 3531005: Coronary artery angioplasty |  |  |
| **Stroke**  Self-report of stroke diagnosis or hospitalization with diagnostic codes for stroke (ischemic or non-ischaemic) | 1. Self-report questionnaire | Have been told by a doctor that you have had a stroke? (Q.B15)  How old were you when you had your first stroke? (Q.B16)  Current treatments of stroke (Q.B17) |  |  |
|  | 1. National Minimum Dataset (NMDS)   (hospitalizations with discharge codes) | **ICD-10AM codes**  G460-468: Vascular syndromes of brain in cerebrovascular diseases  I610-I616, I618, I619: Intracerebral hemorrhage  I630-I636, I638, I639: Cerebral infarction  I64: Stroke, not specified as hemorrhage or infarction  I691, I693, I694: Sequelae of intracerebral hemorrhage, Sequelae of cerebral infarction, Sequelae of stroke, not specified as hemorrhage or infarction |  |  |
| **Admission for stroke**  At least one admission for stroke.  Age at first admission for stroke. | National Minimum Dataset (NMDS)  (hospitalizations with discharge codes) | **ICD 10-AM codes:**  G460-468: Vascular syndromes of brain in cerebrovascular diseases  I610-I616, I618, I619: Intracerebral hemorrhage  I630-I636, I638,I639: Cerebral infarction  I64: Stroke, not specified as hemorrhage or infarction  I691, I693, I694: Sequelae of intracerebral hemorrhage, Sequelae of cerebral infarction, Sequelae of stroke, not specified as hemorrhage or infarction |  |  |
| **Peripheral vascular disease**  Any of:  Self-report of diagnosis or treatment of arterial embolism or thrombosis  Hospitalization with diagnostic codes for aortoiliac or peripheral arterial embolism or thrombosis  Procedures codes for aortoiliac or peripheral arterial revascularization procedure (surgical or percutaneous) | 1. Self-reported questionnaire | Do you currently have any other major illnesses? (Q.B41)  Have you ever had any other major illnesses in the past? (Q.B42) |  |  |
|  | 1. National Minimum Dataset (NMDS)   (hospitalizations with discharge codes) | **ICD-10AM diagnostic codes**  I740-I745: Arterial embolism and thrombosis  **ICD-10AM procedure codes**  3270800 – 3270803: Aortic bypass procedure using synthetic material  3271200- 3271201, 3271800: Ilio-femoral bypass procedures  3271500- 3271501, 3271502-3271503, 3271801, 3273900, 3274200, 3274500, 3274800, 3275100, 3275101-3275103, 3275400- 3275402, 3275700- 3275701,: Subclavian- femoral bypass procedures, axillo-femoral bypass procedures, femoro-femoral crossover bypass, femoral artery bypass procedures.  **Additional codes in next box.** | 3276303: Subclavian bypass using vein,  3276306-3276308: aorto-femoro-popliteal bypass using vein, ilioiliac bypass using vein, Popliteal-tibial bypass using vein  3350601, 3350900,3351200,  3351500, 3351501,3351800,  3352100: Subclavian endarterectomy, Aorta endarterectomy, Aorto-iliac endarterectomy, Aorto-femoral endarterectomy, Bilateral Ilio-femoral endarterectomy, Iliac endarterectomy, unilateral Ilio-femoral endarterectomy.  3276310- 3276319: Subclavian-subclavian bypass using synthetic material, Subclavian-vertebral bypass using synthetic material, Subclavian-axillary bypass using synthetic material,  Axillo-axillary bypass using synthetic material, Axillo-brachial bypass using synthetic material, Aorto-coeliac bypass using synthetic material, Aorto-femoro-popliteal bypass using synthetic material, Ilio-iliac bypass using synthetic material, Popliteal-tibial bypass using synthetic material |  |
| **Admission for peripheral vascular disease**  At least one admission for PVD.  Age at first admission for PVD. | 1. National Minimum Dataset (NMDS)   (hospitalizations with discharge codes) | ICD-10-AM codes as for “Peripheral vascular disease” above |  |  |
| **Hospital admission with heart failure**  Admission to hospital with one of the listed diagnostic codes for heart failure.  Age at first admission for heart failure. | 1. National Minimum Dataset (NMDS)   (hospitalizations with discharge codes) | I110 Hypertensive heart disease with heart failure  I130 Hypertensive heart and chronic kidney disease with heart failure and stage 1 through stage 4 chronic kidney disease, or unspecified chronic kidney disease  I132 Hypertensive heart and chronic kidney disease with heart failure and with stage 5 chronic kidney disease, or end stage renal disease  I50, I500-I501, I509: Heart failure, congestive heart failure, Left ventricular failure, heart failure unspecified |  |  |
| General Health Outcomes | | | | |
| **Hierarchical win ratio outcome**  **6 step hierarchical win ratio**  Step 1: time to death after randomization  Step 2: Age at first major adverse cardiovascular event  Step 3: diagnosis of diabetes mellitus  Step 4: number of admissions to hospital with respiratory illness as primary reason for admission  Step 5: self-reported general health  Step 6: time in hospital per 10 years alive after 1988 | MORT dataset and death certificates | Date of death and cause of death | **Other outcomes defined elsewhere in this document** |  |
| **Time to death after randomization** | 1. MORT data | Death from any cause and date of death obtained from MORT dataset |  |  |
|  | 1. Death certificates | Death from any cause and date of death obtained from death certificates for those not in the MORT dataset. |  |  |
|  | 1. Trial follow up records | Death from any cause and date of death as recorded in databases for original trial data and earlier follow-up studies. |  |  |
| **All-cause mortality**  Death from any cause identified in any of the listed datasets | 1. MORT data | Death from any cause obtained from MORT dataset |  |  |
|  | 1. Death certificates | Death from any cause obtained from death certificates for those not in the MORT dataset. |  |  |
|  | 1. Trial follow up records | Death from any cause as recorded in databases for original trial data and earlier follow-up studies. |  |  |
| **Time in hospital per 10 years alive from 1988** | MORT data and NMDS admissions data | Calculated as cumulative number of days admitted to hospital from NMDS admissions data divided by number of years alive after 1988, multiplied by 10. | Denominator will be those with data available for mortality or data linkage. |  |
| **Self-reported general health**  Ordinal and categorical  (proportion with **r**eported health excellent, very good or good and proportion with reported health fair or poor) | Self-report questionnaire | In general, would you say your health is- excellent, very good, good, fair, poor? (Q. E1) |  |  |
| Respiratory disease | | | | |
| **Asthma or chronic obstructive pulmonary disease COPD**  Defined as the presence of any of:   1. Self-reported diagnosis of asthma 2. IPAG score suggestive of COPD (score >19.5) 3. Admissions with diagnostic codes for asthma or chronic obstructive pulmonary disease (COPD) 4. Dispensing of pharmaceuticals for asthma or COPD | Based on definitions below outcomes. |  |  |  |
| **Number of admissions to hospital with respiratory illness as the primary reason for admission**  Admission with primary diagnostic code for respiratory illness (see listed codes) | National minimum dataset admissions data and diagnostic codes | Hospital admissions with one of the following primary diagnostic codes:  J09-J18 Influenza and pneumonia, J20-22 other acute lower respiratory infections, J40-J47 chronic lower respiratory diseases, J80-84 other respiratory disease affecting the interstitium, J85-86 suppurative and necrotic conditions of the lower respiratory tract, J96 respiratory failure not elsewhere classified, J98 other respiratory disorders |  |  |
| Respiratory | | | | |
| **Asthma**  Defined as the presence of either of:   1. Self-report of a doctor’s diagnosis of asthma 2. Hospital admissions with diagnostic codes for asthma 3. Outpatient events with codes for asthma | 1. Self-report questionnaire | Have you ever been told by a doctor that you have asthma? (Q. B22)  What treatments do you now have for asthma? (Q. B23) | If answers no to question about asthma but has evidence of asthma from only one other data source then will be adjudicated. |  |
|  | 1. NMDS admissions data | Admissions with the following ICD-10AM codes:  J45.0 – J45.998 Asthma and subcodes.  J46 status asthmaticus |  |  |
| **Chronic obstructive pulmonary disease**  Defined as the presence of either of:   1. Self-report of a doctor’s diagnosis of COPD, emphysema or chronic bronchitis 2. Hospital admissions with diagnostic codes for COPD 3. International primary care airways group screening tool score >19.5 (see below) | 1. Self-report questionnaire | Do you currently have any other major illnesses? (Q.B41)  Have you ever had any other major illnesses in the past? (Q.B42) | If answers no to question about COPD but has evidence of COPD from only one other data source then will be adjudicated. |  |
|  | 1. NMDS admissions data | **ICD-10AM codes:**  J410, J411, J418, J42: Chronic bronchitis  J430-J432, J438, J439: Emphysema.  J440-J441, J448-J449: Chronic obstructive pulmonary disease |  |  |
| **International primary care airways group screening tool score** [4] **of >19.5** | 1. Score derived from answers to Self-report questionnaire | Do you have a cough that is affected by the weather? Y/N (Q. B24)  Do you suffer from sputum (phlegm) production even when you don't have a cold? Y/N(Q. B25)  Do you suffer from sputum (phlegm) production in the morning? Y/N (Q. B26)  How often do you have wheezing? Never/sometimes/often. (Q.B27)  Do you currently smoke? (QH1),  How many cigarettes do you smoke daily? (QH2):  Have you ever been a smoker in the past? (QH3). How many cigarettes did you smoke daily in the past (QH4) Less than 1 per day, past?. How many years in your life have you/did you smoke daily or almost daily? (QH5). | IPAG score [4]:  Age ≥ 50 score 4, age <50 score 0.  Smoking pack years: 0-15 (0), 15-25 (2), 25-50(3), ≥50(7).  BMI: <25.4 (5), 25.4-29.7 (1), ≥29.7 (0).  Do you have a cough that is affected by the weather? Y (3)/N (0).  Do you suffer from sputum (phlegm) production even when you don't have a cold? Y(3)/N(0).  Do you suffer from sputum (phlegm) production in the morning? Y(0)/N(3).  How often do you have wheezing? Never (0)/sometimes/often(4).  Have you ever been told by a doctor that you have allergies? Y(0)/N(3).  Score /32 |  |
| **Admissions for asthma or chronic obstructive pulmonary disease**  Hospital admission/s with diagnostic codes for Asthma, chronic bronchitis, emphysema or chronic obstructive pulmonary disease | 1. NMDS admissions data (hospitalizations with discharge codes) | **ICD-10AM codes:**  J410, J411, J418, J42: Chronic bronchitis  J430-J432, J438, J439: Emphysema.  J440-J441, J448-J449: Chronic obstructive pulmonary disease  J450-J451, J458, J459: Asthma  J46: Status asthmaticus  J8283 Eosinophilic asthma |  |  |
| **Dispensing of pharmaceuticals for either asthma or chronic obstructive pulmonary disease**   1. Dispensing of 2 or prescriptions for one of the listed medications | 1. NZ pharmaceuticals collection | Prescriptions + dates  **Short acting beta agonist (SABA):** Salbutamol (*Ventolin, respigen, SalAir)*, terbutaline (*Bricanyl*).  **Long acting beta agonists (LABA)**: Eformoterol (formoterol fumarate dehydrate, *Oxis, Foradil)*), salmeterol (*Serevent, Meterol),* Indacterol *(OnBrez*)  **Short acting muscarinic antagonist (SAMA):**  Ipratropium (*Atrovent, Univent*)  **Long acting muscarinic antagonist (LAMA):** tiotropium (*Spiriva, Spiriva Respimat*), glycopyrronium (*SeeBri*), and umeclidinium (*Incruse Ellipta*).  **Inhaled corticosteroid(ICS):** Beclometasone dipropionate (*Beclazone, Qvar*), budesonide (*Pulmicort*), fluticasone propionate (*Flixotide, Floair*)  **Combination of SABA and SAMA:**  ipratropium bromide + salbutamol *(Duolin HFA)*  **Combination of LABA and ICS:**  fluticasone + salmeterol (*Seretide, RexAir*), budesonide + formoterol (*Symbicort,DueResp Spiromax, Vannair),* fluticasone furoate + vilanterol *(Breo Ellipta, Relvar Ellipta)*  **Combination of LABA and LAMA:**  indacaterol + glycopyrrolate (*Ultibro Breezhaler*), tiotropium + olodaterol (*Spiolto Respimat*), umeclidinium + vilanterol (*Anoro Ellipta).*  **Combination of LAMA and ICS:**  **Oral Treatments**  Theophylline (*Nuelin SR)*  Montelukast (*Montelukast Mylan, Apo-Montelukast, Montelukast Accord*)  **Other**: Omalizumab |  |  |
| Tertiary Outcomes | | | | |
| Cardiovascular | | | | |
| **Heart failure**  Defined as the presence of any of:   1. Self-reported diagnosis of heart failure 2. Hospital admission/s with a diagnosis of heart failure (see listed codes) 3. Attendance for outpatient integrated care for heart failure | 1. Self-reported questionnaire | Have you ever been told  by a doctor that you have heart  failure? (Q.B12) |  |  |
|  | 1. National Minimum Dataset (NMDS)   (hospitalizations with discharge codes) | I110 Hypertensive heart disease with heart failure  I130 Hypertensive heart and chronic kidney disease with heart failure and stage 1 through stage 4 chronic kidney disease, or unspecified chronic kidney disease  I132 Hypertensive heart and chronic kidney disease with heart failure and with stage 5 chronic kidney disease, or end stage renal disease  I50, I500-I501, I509: Heart failure, congestive heart failure, Left ventricular failure, heart failure unspecified |  |  |
|  | 1. NNPAC | Outpatient event codes:  COOC0024 (Integrated care for heart failure) |  |  |
| **Atrial fibrillation**  Defined as the presence of either of:   1. Self-reported diagnosis of atrial fibrillation 2. Hospital admission/s with a diagnostic code for atrial fibrillation | 1. Self-reported questionnaire | 1. Have you ever been told by a doctor that you have any other heart condition/s? (Q.B13)   (If answers with atrial fibrillation) |  |  |
|  | 1. National Minimum Dataset (NMDS)   (hospitalizations with discharge codes) | I480-I489: Paroxysmal atrial fibrillation, persistent atrial fibrillation, chronic atrial fibrillation, unspecified atrial fibrillation |  |  |
| Diabetes mellitus | | | | |
| **Type 1 diabetes mellitus**  Either of:   1. Self-report of diagnosis of type 1 diabetes mellitus 2. Hospital admission/s with a diagnosis code for type 1 diabetes mellitus | 1. Self- report questionnaire (Q.B18-21) | Told by doctor you have diabetes – excluding GDM (Q.B18)  Type of diabetes you have been told you have (Type 1, Type 2) (Q.B19)  Age at diagnosis (Q.B20)  Treatment type (No treatment, insulin, medicines, tablets, pills, diet, exercise, other) (Q.B21) | If reports type 1 diabetes mellitus and is not on insulin assume type 2.  If not sure of diabetes type and on medicines but not insulin then assume type 2. |  |
|  | 1. NMDS | Hospital admissions with a diagnosis of diabetes mellitus. This includes the following ICD 10 codes:  E10 (Type 1 diabetes mellitus), O24.0 (pre-existing Type 1 diabetes mellitus during pregnancy) |  |  |
| **Type 2 diabetes mellitus**  Either of:   1. Self-report of diagnosis of type 2 diabetes mellitus   Hospital admission/s with a diagnosis code for type 2 diabetes mellitus | 1. Self- report questionnaire (Q.B18-21) | Told by doctor you have diabetes – excluding GDM (Q.B18)  Type of diabetes you have been told you have (Type 1, Type 2) (Q.B19)  Age at diagnosis (Q.B20)  Treatment type (No treatment, insulin, medicines, tablets, pills, diet, exercise, other) (Q.B21) | If not sure of diabetes type and on medicines but not insulin then assume type 2. |  |
|  | 1. NMDS | Hospital admissions with a diagnosis of diabetes mellitus. This includes the following ICD 10 codes:  E11 (Type 2 diabetes mellitus), O24.1 (pre-existing type 2 diabetes mellitus) |  |  |

General Notes:

ICD-10AM 2^nd^ edition codes used for 2001 onwards.

ICD-9 for prior to 2001, mapped to ICD10AM 2^nd^ edition.

**References**

1. New Zealand Society for the Study of Diabetes. Type 2 Diabetes Management Guidance. 2021 [cited 8 Dec 2021]. Available: https://t2dm.nzssd.org.nz/

2. Stone NJ, Robinson JG, Lichtenstein AH, Bairey Merz CN, Blum CB, Eckel RH, et al. 2013 ACC/AHA guideline on the treatment of blood cholesterol to reduce atherosclerotic cardiovascular risk in adults: A report of the American College of Cardiology/American Heart Association task force on practice guidelines. Circulation. 2014;129. doi:10.1161/01.cir.0000437738.63853.7a

3. Hicks KA, Mahaffey KW, Mehran R, Nissen SE, Wiviott SD, Dunn B, et al. 2017 Cardiovascular and Stroke Endpoint Definitions for Clinical Trials. Circulation. 2018;137: 961–972. doi:10.1161/CIRCULATIONAHA.117.033502

4. Price DB, Tinkelman DG, Nordyke RJ, Isonaka S, Halbert RJ, COPD Questionnaire Study Group. Scoring system and clinical application of COPD diagnostic questionnaires. Chest. 2006;129: 1531–1539. doi:10.1378/chest.129.6.1531
