# Supplementary material for: Cardiovascular outcomes 50 years after antenatal exposure to betamethasone: Follow-up of a randomised double-blind, placebo-controlled trial
Source: PLoS Med. 2024 Apr 1;21(4):e1004378. doi: 10.1371/journal.pmed.1004378 (PMC11018286; doi:10.1371/journal.pmed.1004378)
Supplement: S2 Appendix — Table A. Tertiary outcomes. Fig A. Wins and losses for betamethasone at each step of the 6-step unmatched win ratio. Table B. Subgroup analyses for primary outcomes. Table C. Subgroup analyses for secondary outcomes. Table D. Sensitivity analysis for win ratio hierarchical outcome. Table E. Sensitivity analysis for primary and secondary outcomes. Table F. Post hoc subgroup analysis for primary outcomes and components by gestational age at delivery. (DOCX) [file pmed.1004378.s004.docx]

Table of Contents

[Table A: Tertiary outcomes 2](#_Toc152165533)

[Figure A Wins and losses for betamethasone at each step of 6-step unmatched win ratio 3](#_Toc152165534)

[Table B: Subgroup analyses for primary outcomes 4](#_Toc152165535)

[Table C Subgroup analyses for secondary outcomes 6](#_Toc152165536)

[Table D: Sensitivity analysis for win ratio hierarchical outcome. 34](#_Toc152165537)

[Table E: Sensitivity analyses for primary and secondary outcomes. 35](#_Toc152165538)

[Table F: Post hoc subgroup analysis for primary outcomes and components by gestational age at delivery 51](#_Toc152165539)

Table A: Tertiary outcomes

| **Outcome** | **Betamethasone** | **Placebo** | **Unadjusted RR (95% CI)** | **Adjusted* RR (95% CI)** | **P value**  **(adjusted*)** |
| --- | --- | --- | --- | --- | --- |
| Type 2 diabetes mellitus, n/N (%) | 15/229 (6.6%) | 18/195 (9.2%) | 0.71 (0.37, 1.37) | 0.67 (0.33, 1.38) | 0.25 |
| Type 1 diabetes mellitus, n/N (%) | 0/229 (0%) | 1/195 (0.5%) | NE | NE | NE |
| Heart failure, n/N (%) | 2/229 (0.9%) | 4/195 (2.1%) | 0.43 (0.08, 2.31) | 0.41 (0.07, 2.55) | 0.32 |
| Atrial fibrillation, n/N (%) | 3/229 (1.3%) | 5/195 (2.6%) | 0.51 (0.12, 2.12) | 0.57 (0.12, 2.66) | 0.45 |

CI: Confidence interval. RR: Relative risk

*Analyses adjusted for sex, gestational age at trial entry and for clustering.

Figure A Wins and losses for betamethasone at each step of 6-step unmatched win ratio

| 1. **Win category** | **Number of wins (% of total comparisons)** | **b)** |
| --- | --- | --- |
| **Step 1: Time to death after randomization** |  |  |
| Betamethasone (BM) wins (only placebo died or died before betamethasone) | 89,041 (24.0%) |  |
| Placebo wins at step 1 (only BM died or died before placebo) | 72,820 (19.6%) |  |
| **Step 2: Time to first major adverse cardiovascular event** |  |  |
| BM wins (only placebo had cardiovascular event or placebo had event first) | 2,298 (0.6%) |  |
| Placebo wins (only BM had cardiovascular event or BM had event first) | 2,247 (0.6%) |  |
| **Step 3: Diagnosis of diabetes mellitus** |  |  |
| BM wins (only placebo had diagnosis of diabetes mellitus) | 4,009 (1.1%) |  |
| Placebo wins (only BM had diagnosis of diabetes mellitus) | 2,184 (0.6%) |  |
| **Step 4: Number of hospital admissions for respiratory illness** |  |  |
| BM wins (placebo had more hospital admissions for respiratory illness) | 2,146 (0.6%) |  |
| Placebo wins (BM had more hospital admissions for respiratory illness) | 2,617 (0.7%) |  |
| **Step 5: Self-reported general health** |  |  |
| BM wins (self-reported general health was better for BM) | 1,891 (0.5%) |  |
| Placebo wins (self-reported general health was better for placebo) | 3,560 (1.0%) |  |
| **Step 6: Time in hospital since 1988** |  |  |
| Betamethasone wins (Placebo had more days in hospital per 10 years alive) | 10,889 (2.9%) |  |
| Placebo wins (BM had more days in hospital per 10 years alive) | 10,300 (2.8%) |  |
| **Ties after all steps (no win determined by the above steps)** | 166,815 (45.0%) |  |

a) Table of wins at each level of the 6-step win ratio. b) Wins and ties at each level of 6-step win ratio. A win represents a favourable result for betamethasone for a pairwise comparison between a betamethasone and placebo group participant at this level of the hierarchy of six outcomes. Every possible pair of betamethasone and placebo participants are compared at each level until a win for either betamethasone or placebo is determined for the pair. BM: Betamethasone.

Table B: Subgroup analyses for primary outcomes

| **Outcome and sub-group** | **Betamethasone** | **Placebo** | **Unadjusted RR or HR (95% CI)** | **Adjusted* RR or HR (95% CI)** | **P value**  **(adjusted*)** | **Interaction P value** |
| --- | --- | --- | --- | --- | --- | --- |
| **Cardiometabolic risk factor composite, n/N (%)** | | | | | | |
| **Trial protocol** |  | | | | | 0.432 |
| Standard-dose treatment | 97/145 (66.9) | 83/123 (67.5) | RR 0.99 (0.84, 1.17) | RR 0.98 (0.83, 1.16) | 0.852 |  |
| Double-standard dose treatment | 62/84 (73.8) | 48/72 (66.7) | RR 1.11 (0.90, 1.36) | RR 1.10 (0.89, 1.35) | 0.394 |  |
| **Multiple Pregnancy** |  | | | | | 0.482 |
| Singleton | 138/195 (70.8) | 117/175 (66.9) | RR 1.06 (0.92, 1.22) | RR 1.05 (0.91, 1.20) | 0.528 |  |
| Multiple | 21/34 (61.8) | 14/20 (70.0) | RR 0.88 (0.59, 1.32) | RR 0.90 (0.61, 1.33) | 0.600 |  |
| **Infant sex** |  | | | | | 0.602 |
| Female | 71/105 (67.6) | 68/107 (63.6) | RR 1.06 (0.87, 1.29) | RR 1.06 (0.87, 1.29) | 0.535 |  |
| Male | 88/124 (71.0) | 63/88 (71.6) | RR 0.99 (0.83, 1.18) | RR 0.99 (0.83, 1.18) | 0.928 |  |
| **Tocolytic used** |  | | | | | 0.258 |
| Ethanol | 23/38 (60.5) | 21/29 (72.4) | RR 0.84 (0.59, 1.18) | RR 0.83 (0.59, 1.17) | 0.289 |  |
| Salbutamol | 71/111 (64.0) | 60/96 (62.5) | RR 1.02 (0.83, 1.26) | RR 1.01 (0.82, 1.25) | 0.926 |  |
| Both | 7/7 (100) | 5/5 (100) | RR 1.00 (NE) | RR 0.98 (0.80, 1.19) | 0.820 |  |
| None | 58/73 (79.5) | 45/65 (69.2) | RR 1.15 (0.94, 1.40) | RR 1.15 (0.94, 1.41) | 0.169 |  |
| **Reason for preterm birth** |  | | | | | 0.636 |
| Unplanned preterm birth | 132/193 (68.4) | 107/161 (66.5) | RR 1.03 (0.89, 1.19) | RR 1.02 (0.88, 1.18) | 0.823 |  |
| Hypertensive disorders of pregnancy | 17/20 (85.0) | 14/19 (73.7) | RR 1.15 (0.82, 1.62) | RR 1.23 (0.88, 1.72) | 0.234 |  |
| Rhesus iso-immunization | 6/10 (60.0) | 5/8 (62.5) | RR 0.96 (0.43, 2.13) | RR 0.92 (0.44, 1.91) | 0.816 |  |
| Placenta previa | 2/4 (50.0) | 3/4 (75.0) | RR 0.67 (0.16, 2.74) | RR 0.66 (0.21, 2.11) | 0.487 |  |
| Diabetes mellitus | 2/2 (100) | 2/3 (66.7) | RR 1.44 (0.41, 5.00) | RR 1.66 (1.43, 1.93) | <0.001 |  |
| **Age at first major adverse cardiovascular event, years, median (5^th^, 95^th^ centile)** | | | | | | |
| **Trial protocol** |  | | | | | 0.528 |
| Standard-dose treatment | 38.6 (20.5, 48.5) | 46.4 (37.6, 50.3) | HR 0.47 (0.14, 1.55) | HR 0.46 (0.14, 1.55) | 0.212 |  |
| Double-standard dose treatment | 42.2 (41.8, 44.6) | 33.9 (24.2, 43.8) | HR 0.82 (0.18, 3.67) | HR 0.86 (0.19, 3.86) | 0.845 |  |
| **Multiple Pregnancy** |  | | | | | 0.210 |
| Singleton | 42.0 (20.5, 48.5) | 46.4 (40.3, 50.3) | HR 0.78 (0.27, 2.25) | HR 0.79 (0.27, 2.28) | 0.663 |  |
| Multiple | 41.4 (41.4, 41.4) | 33.9 (24.2, 37.6) | HR 0.17 (0.02, 1.52) | HR 0.17 (0.02, 1.50) | 0.109 |  |
| **Infant sex** |  | | | | | 0.987 |
| Female | - | 42.3 (24.2, 47.6) | NE | NE | NE |  |
| Male | 41.8 (20.5, 48.5) | 44.9 (31.9, 50.3) | HR 1.08 (0.36, 3.22) | HR 1.11 (0.37, 3.31) | 0.850 |  |
| **Tocolytic used** |  | | | | | 0.999 |
| Ethanol | - | 47.1 (47.1, 47.1) | NE | NE | NE |  |
| Salbutamol | 43.2 (35.7, 48.5) | 46.7 (37.6, 50.3) | HR 0.80 (0.22, 2.99) | HR 0.80 (0.22, 2.99) | 0.744 |  |
| Both | - | 40.3 (40.3, 40.3) | NE | NE | NE |  |
| None | 41.4 (20.5, 42.2) | 35.9 (24.2, 46.0) | HR 0.69 (0.16, 2.88) | HR 0.70 (0.17, 2.96) | 0.629 |  |
| **Reason for preterm birth** |  | | | | | 1.000 |
| Unplanned preterm birth | 42.0 (35.7, 48.5) | 43.8 (24.2, 50.3) | HR 0.52 (0.19, 1.40) | HR 0.52 (0.19, 1.41) | 0.199 |  |
| Hypertensive disorders of pregnancy | 20.5 (20.5, 20.5) | - | NE | NE | NE |  |
| Rhesus iso-immunization | - | - | NE | NE | NE |  |
| Placenta previa | - | - | NE | NE | NE |  |
| Diabetes mellitus | - | 44.2 (44.2, 44.2) | NE | NE | NE |  |

CI: Confidence interval. HR: Hazard ratio. RR: Relative risk.

*Analyses adjusted for sex and gestational age at trial entry.

Table C Subgroup analyses for secondary outcomes

| **Outcome and sub-group** | **Betamethasone** | **Placebo** | **Unadjusted RR, HR or mean difference**  **(95% CI)** | **Adjusted* RR, HR or mean difference**  **(95% CI)** | **P value**  **(adjusted*)** | **Interaction p value** |
| --- | --- | --- | --- | --- | --- | --- |
| **Diabetes mellitus or prediabetes or gestational diabetes mellitus, n/N (%)** | | | | | | |
| **Trial protocol** |  | | | | | 0.621 |
| Standard-dose treatment | 33/145 (22.8) | 26/123 (21.1) | RR 1.08 (0.68, 1.70) | RR 1.07 (0.67, 1.69) | 0.784 |  |
| Double-standard dose treatment | 15/84 (17.9) | 15/72 (20.8) | RR 0.86 (0.45, 1.64) | RR 0.87 (0.46, 1.67) | 0.682 |  |
| **Multiple Pregnancy** |  | | | | | 0.136 |
| Singleton | 42/195 (21.5) | 34/175 (19.4) | RR 1.11 (0.74, 1.66) | RR 1.11 (0.74, 1.66) | 0.623 |  |
| Multiple | 6/34 (17.7) | 7/20 (35.0) | RR 0.50 (0.19, 1.32) | RR 0.51 (0.20, 1.31) | 0.161 |  |
| **Infant sex** |  | | | | | 0.509 |
| Female | 22/105 (21.0) | 20/107 (18.7) | RR 1.12 (0.65, 1.93) | RR 1.14 (0.66, 1.97) | 0.635 |  |
| Male | 26/124 (21.0) | 21/88 (23.9) | RR 0.88 (0.53, 1.46) | RR 89 (0.53, 1.48) | 0.648 |  |
| **Tocolytic used** |  | | | | | 0.532 |
| Ethanol | 13/38 (34.2) | 6/29 (20.7) | RR 1.65 (0.70, 3.88) | RR 1.63 (0.70, 3.77) | 0.254 |  |
| Salbutamol | 18/111 (16.2) | 20/96 (20.8) | RR 0.78 (0.44, 1.39) | RR 0.78 (0.44, 1.39) | 0.396 |  |
| Both | 0/7 (0.0) | 2/5 (40.0) | NE | NE | NE |  |
| None | 17/73 (23.3) | 13/65 (20.0) | RR 1.19 (0.61, 2.22) | RR 1.17 (0.61, 2.22) | 0.637 |  |
| **Reason for preterm birth** |  | | | | | 0.991 |
| Unplanned preterm birth | 43/193 (22.3) | 34/161 (21.1) | RR 1.06 (0.71, 1.57) | RR 1.08 (0.72, 1.61) | 0.724 |  |
| Hypertensive disorders of pregnancy | 3/20 (15.0) | 4/19 (21.1) | RR 0.71 (0.17, 2.90) | RR 0.74 (0.19, 2.88) | 0.659 |  |
| Rhesus iso-immunization | 2/10 (20.0) | 0/8 (0.0) | NE | NE | NE |  |
| Placenta previa | 0/4 (0.0) | 1/4 (25.0) | NE | NE | NE |  |
| Diabetes mellitus | 0/2 (0.0) | 2/3 (66.7) | NE | NE | NE |  |
| **Diabetes Mellitus (any type), n/N (%)** | | | | | | |
| **Trial protocol** |  | | | | | 0.356 |
| Standard-dose treatment | 8/145 (5.5) | 14/123 (11.4) | RR 0.48 (0.21, 1.12) | RR 0.47 (0.21, 1.08) | 0.075 |  |
| Double-standard dose treatment | 7/84 (8.3) | 7/72 (9.7) | RR 0.86 (0.31, 2.35) | RR 0.86 (0.32, 2.35) | 0.771 |  |
| **Multiple Pregnancy** |  | | | | | 0.969 |
| Singleton | 15/195 (7.7) | 17/175 (9.7) | RR 0.79 (0.41, 1.54) | RR 0.77 (0.40, 1.51) | 0.449 |  |
| Multiple | 0/34 (0.0) | 4/20 (20.0) | NE | NE | NE |  |
| **Infant sex** |  | | | | | 0.426 |
| Female | 7/105 (6.7) | 9/107 (8.4) | RR 0.79 (0.30, 2.06) | RR 0.80 (0.31, 2.08) | 0.644 |  |
| Male | 8/124 (6.5) | 12/88 (13.6) | RR 0.47 (0.20, 1.11) | RR 0.48 (0.20, 1.12) | 0.088 |  |
| **Tocolytic used** |  | | | | | 0.995 |
| Ethanol | 2/38 (5.3) | 3/29 (10.3) | RR 0.51 (0.09, 2.94) | RR 0.49 (0.09, 2.74) | 0.413 |  |
| Salbutamol | 8/111 (7.2) | 11/96 (11.5) | RR 0.63 (0.26, 1.51) | RR 0.62 (0.26, 1.48) | 0.277 |  |
| Both | 0/7 (0.0) | 0/5 (0.0) | RR 1.00 (NE) | RR 0.93 (NE) | 1.000 |  |
| None | 5/73 (6.9) | 7/65 (10.8) | RR 0.64 (0.21, 1.93) | RR 0.63 (0.21, 1.90) | 0.409 |  |
| **Reason for preterm birth** |  | | | | | 0.999 |
| Unplanned preterm birth | 13/193 (6.7) | 16/161 (9.9) | RR 0.68 (0.34, 1.37) | RR 0.68 (0.34, 1.39) | 0.92 |  |
| Hypertensive disorders of pregnancy | 1/20 (5.0) | 2/19 (10.5) | RR 0.48 (0.04, 5.21) | RR 0.49 (0.05, 5.04) | 0.550 |  |
| Rhesus iso-immunization | 1/10 (10.0) | 0/8 (0.0) | NE | NE | NE |  |
| Placenta previa | 0/4 (0.0) | 1/4 (25.0) | NE | NE | NE |  |
| Diabetes mellitus | 0/2 (0.0) | 2/3 (66.7) | NE | NE | NE |  |
| **Prediabetes, n/N (%)** | | | | | | |
| **Trial protocol** |  | | | | | 0.495 |
| Standard-dose treatment | 23/145 (15.9) | 12/122 (9.8) | RR 1.61 (0.84, 3.11) | RR 1.58 (0.81, 3.06) | 0.177 |  |
| Double-standard dose treatment | 7/83 (8.4) | 6/72 (8.3) | RR 1.01 (0.35, 2.90) | RR 1.03 (0.36, 2.92) | 0.963 |  |
| **Multiple Pregnancy** |  | | | | | 0.498 |
| Singleton | 25/194 (12.9) | 15/175 (8.6) | RR 1.50 (0.82, 2.76) | RR 1.49 (0.81, 2.75) | 0.198 |  |
| Multiple | 5/34 (14.7) | 3/19 (15.8) | RR 0.93 (0.24, 3.59) | RR 0.90 (0.24, 3.41) | 0.881 |  |
| **Infant sex** |  | | | | | 0.959 |
| Female | 12/105 (11.4) | 9/107 (8.4) | RR 1.36 (0.59, 3.10) | RR 1.38 (0.61, 3.16) | 0.440 |  |
| Male | 18/123 (14.6) | 9/87 (10.3) | RR 1.41 (0.66, 3.01) | RR 1.42 (0.67, 3.03) | 0.357 |  |
| **Tocolytic used** |  | | | | | 0.558 |
| Ethanol | 11/38 (29.0) | 3/29 (10.3) | RR 2.80 (0.84, 9.33) | RR 2.76 (0.85, 9.01) | 0.092 |  |
| Salbutamol | 9/110 (8.2) | 8/95 (8.4) | RR 0.97 (0.39, 2.43) | RR 0.96 (0.38, 2.39) | 0.922 |  |
| Both | 0/7 (0.0) | 2/5 (40.0) | NE | NE | NE |  |
| None | 10/73 (13.7) | 5/65 (7.7) | RR 1.78 (0.64, 4.98) | RR 1.78 (0.6, 4.95) | 0.271 |  |
| **Reason for preterm birth** |  | | | | | 1.000 |
| Unplanned preterm birth | 28/192 (14.6) | 17/160 (10.6) | RR 1.37 (0.78, 2.42) | RR 1.37 (0.77, 2.42) | 0.282 |  |
| Hypertensive disorders of pregnancy | 1/20 (5.0) | 1/19 (5.3) | RR 0.95 (0.06, 15.48) | RR 1.02 (0.07, 15.28) | 0.989 |  |
| Rhesus iso-immunization | 1/10 (10.0) | 0/8 (0.0) | NE | NE | NE |  |
| Placenta previa | 0/4 (0.0) | 0/4 (0.0) | NE | NE | NE |  |
| Diabetes mellitus | 0/2 (0.0) | 0/3 (0.0) | NE | NE | NE |  |
| **Gestational diabetes mellitus, n/N (%)** | | | | | | |
| **Trial protocol** |  | | | | | 0.974 |
| Standard-dose treatment | 3/66 (4.6) | 0/72 (0.0) | NE | NE | NE |  |
| Double-standard dose treatment | 4/39 (10.3) | 3/34 (8.8) | RR 1.16 (0.27, 4.95) | RR 1.34 (0.32, 5.66) | 0.685 |  |
| **Multiple Pregnancy** |  | | | | | 0.396 |
| Singleton | 6/92 (6.5) | 2/94 (2.1) | RR 3.07 (0.63, 14.95) | RR 3.40 (0.69, 16.69) | 0.131 |  |
| Multiple | 1/13 (7.7) | 1/12 (8.3) | RR 0.92 (0.06, 15.27) | RR 0.88 (0.06, 12.79) | 0.928 |  |
| **Infant sex** |  | | | | | |
| Female |  |  |  |  |  |  |
| Male |  |  |  |  |  |  |
| **Tocolytic used** |  | | | | | 0.994 |
| Ethanol | 0/14 (0.0) | 0/14 (0.0) | NE | NE | NE |  |
| Salbutamol | 4/53 (7.6) | 2/53 (3.8) | RR 2.00 (0.38, 10.66) | RR 2.14 (0.40, 11.37) | 0.369 |  |
| Both | 0/3 (0.0) | 0/4 (0.0) | NE | NE | NE |  |
| None | 3/35 (8.6) | 1/35 (2.9) | RR 3.00 (0.31, 28.58) | RR 3.21 (0.35, 29.71) | 0.303 |  |
| **Reason for preterm birth** |  | | | | | 0.819 |
| Unplanned preterm birth | 6/88 (6.8) | 2/89 (2.3) | RR 3.03 (0.62, 14.79) | RR 3.22 (0.66, 15.78) | 0.149 |  |
| Hypertensive disorders of pregnancy | 1/14 (7.1) | 1/10 (10.0) | RR 0.71 (0.04, 11.80) | RR 0.73 (0.05, 10.42) | 0.817 |  |
| Rhesus iso-immunization | 0/1 (0.0) | 0/5 (0.0) | NE | NE | NE |  |
| Placenta previa | 0/1 (0.0) | 0/2 (0.0) | NE | NE | NE |  |
| Diabetes mellitus | 0/1 (0.0) | 0/0 (0.0) | NE | NE | NE |  |
| **Hypertension, n/N (%)** | | | | | | |
| **Trial protocol** |  | | | | | 0.554 |
| Standard-dose treatment | 45/144 (31.3) | 42/123 (34.2) | RR 0.92 (0.65, 1.29) | RR 0.90 (0.64, 1.28) | 0.562 |  |
| Double-standard dose treatment | 27/84 (32.1) | 22/72 (30.6) | RR 1.05 (0.66, 1.68) | RR 1.08 (0.67, 1.72) | 0.759 |  |
| **Multiple Pregnancy** |  | | | | | 0.259 |
| Singleton | 61/194 (31.4) | 54/175 (30.9) | RR 1.02 (0.75, 1.38) | RR 1.02 (0.75, 1.30) | 0.912 |  |
| Multiple | 11/34 (32.3) | 10/20 (50.0) | RR 0.65 (0.33, 1.26) | RR 0.67 (0.35, 1.30) | 0.234 |  |
| **Infant sex** |  | | | | | 0.928 |
| Female | 32/104 (30.8) | 34/107 (31.8) | RR 0.97 (0.65, 1.45) | RR 0.98 (0.65, 1.46) | 0.901 |  |
| Male | 40/124 (32.3) | 30/88 (34.1) | RR 0.95 (0.64, 1.40) | RR 0.95 (0.65, 1.40) | 0.796 |  |
| **Tocolytic used** |  | | | | | 0.988 |
| Ethanol | 12/37 (32.4) | 9/29 (31.0) | RR 1.05 (0.50, 2.17) | RR 1.01 (0.49, 2.08) | 0.972 |  |
| Salbutamol | 34/111 (30.6) | 30/96 (31.3) | RR 0.98 (0.65,1.48) | RR 0.99 (0.65, 1.48) | 0.944 |  |
| Both | 3/7 (42.9) | 3/5 (60.0) | RR 0.71 (0.20, 2.54) | RR 0.82 (0.26, 2.55) | 0.726 |  |
| None | 23/73 (31.5) | 22/65 (33.9) | RR 0.93 (0.58, 1.51) | RR 0.93 (0.58, 1.51) | 0.770 |  |
| **Reason for preterm birth** |  | | | | | 0.807 |
| Unplanned preterm birth | 57/192 (29.7) | 51/161 (31.7) | RR 0.94 (0.68, 1.28) | RR 0.94 (0.69, 1.29) | 0.700 |  |
| Hypertensive disorders of pregnancy | 8/20 (40.0) | 8/19 (42.1) | RR 0.95 (0.44, 2.07) | RR 0.95 (0.45, 2.02) | 0.894 |  |
| Rhesus iso-immunization | 5/10 (50.0) | 2/8 (25.0) | RR 2.00 (0.46, 8.62) | RR 2.10 (0.54, 8.22) | 0.286 |  |
| Placenta previa | 1/4 (25.0) | 1/4 (25.0) | RR 1.00 (0.05, 20.02) | RR 1.02 (0.09, 11.34) | 0.986 |  |
| Diabetes mellitus | 1/2 (50.0) | 2/3 (66.7) | RR 0.75 (0.06, 10.08) | RR 0.59 (0.11, 3.09) | 0.533 |  |
| **Hyperlipidemia, n/N (%)** | | | | | | |
| **Trial protocol** |  | | | | | 0.364 |
| Standard-dose treatment | 81/144 (56.3) | 72/122 (59.0) | RR 0.95 (0.77, 1.17) | RR 0.92 (0.75, 1.13) | 0.427 |  |
| Double-standard dose treatment | 50/84 (59.5) | 40/72 (55.6) | RR 1.07 (0.81, 1.41) | RR 1.08 (0.82, 1.41) | 0.595 |  |
| **Multiple Pregnancy** |  | | | | | 0.735 |
| Singleton | 115/194 (59.3) | 102/175 (58.3) | RR 1.02 (0.86, 1.21) | RR 1.00 (0.84, 1.18) | 0.963 |  |
| Multiple | 16/34 (47.1) | 10/19 (52.6) | RR 0.89 (0.51, 1.58) | RR 0.90 (0.52, 1.57) | 0.713 |  |
| **Infant sex** |  | | | | | 0.696 |
| Female | 52/104 (50.0) | 57/107 (53.3) | RR 0.94 (0.72, 1.22) | RR 0.94 (0.72, 1.22) | 0.632 |  |
| Male | 79/124 (63.7) | 55/87 (63.2) | RR 1.01 (0.82, 1.24) | RR 1.00 (0.81, 1.24) | 0.979 |  |
| **Tocolytic used** |  | | | | | 0.558 |
| Ethanol | 18/37 (48.7) | 17/29 (58.6) | RR 0.83 (0.52, 1.31) | RR 0.81 (0.52, 1.27) | 0.358 |  |
| Salbutamol | 59/111 (53.2) | 53/95 (55.8) | RR 0.95 (0.74, 1.23) | RR 0.92 (0.72, 1.18) | 0.510 |  |
| Both | 6/7 (85.7) | 4/5 (80.0) | RR 1.07 (0.58, 1.96) | RR 0.96 (0.50, 1.84) | 0.908 |  |
| None | 48/73 (65.8) | 38/65 (58.5) | RR 1.12 (0.86, 1.47) | RR 1.13 (0.87, 1.46) | 0.365 |  |
| **Reason for preterm birth** |  | | | | | 0.306 |
| Unplanned preterm birth | 111/192 (57.8) | 94/160 (58.8) | RR 0.98 (0.83, 1.18) | RR 0.94 (0.79, 1.12) | 0.472 |  |
| Hypertensive disorders of pregnancy | 13/20 (65.0) | 9/19 (47.4) | RR 1.37 (0.76, 2.48) | RR 1.65 (0.92, 2.96) | 0.093 |  |
| Rhesus iso-immunization | 4/10 (40.0) | 5/8 (62.5) | RR 0.64 (0.23, 1.75) | RR 0.62 (0.25, 1.56) | 0.306 |  |
| Placenta previa | 2/4 (50.0) | 3/4 (75.0) | RR 0.67 (0.16, 2.74) | RR 0.76 (0.24, 2.33) | 0.625 |  |
| Diabetes mellitus | 1/2 (50.0) | 1/3 (33.3) | RR 1.50 (0.05,46.66) | RR 2.06 (0.24, 17.48) | 0.508 |  |
| **At least one admission for major adverse cardiovascular event including cardiovascular death, n/N (%)** | | | | | | |
| **Trial protocol** |  |  |  |  |  | 0.531 |
| Standard-dose treatment | 4/317 (1.3) | 8/300 (2.7) | RR 0.47 (0.14, 1.56) | RR 0.47 (0.14, 1.56) | 0.219 |  |
| Double-standard dose treatment | 3/176 (1.7) | 4/194 (2.1) | RR 0.83 (0.19, 3.66) | RR 0.87 (0.20, 3.83) | 0.852 |  |
| **Multiple Pregnancy** |  |  |  |  |  | 0.232 |
| Singleton | 6/425 (1.4) | 8/444 (1.8) | RR 0.78 (0.27, 2.24) | RR 0.80 (0.28, 2.29) | 0.673 |  |
| Multiple | 1/68 (1.5) | 4/50 (8.0) | RR 0.18 (0.02, 1.63) | RR 0.18 (0.02, 1.60) | 0.126 |  |
| **Infant sex** |  |  |  |  |  | 0.971 |
| Female | 0/209 (0.0) | 6/226 (2.7) | NE | NE | NE |  |
| Male | 7/284 (2.5) | 6/268 (2.2) | RR 1.10 (0.37, 3.24) | RR 1.12 (0.38, 3.31) | 0.832 |  |
| **Tocolytic used** |  |  |  |  |  | 0.999 |
| Ethanol | 0/82 (0.0) | 1/64 (0.0) | NE | NE | NE |  |
| Salbutamol | 4/253 (1.6) | 5/256 (2.0) | RR 0.81 (0.22, 2.99) | RR 0.81 (0.22, 2.9) | 0.751 |  |
| Both | 0/14 (0.0) | 1/10 (10.0) | NE | NE | NE |  |
| None | 3/144 (2.1) | 5/164 (3.1) | RR 0.68 (0.17, 2.83) | RR 0.71 (0.17, 2.94) | 0.639 |  |
| **Reason for preterm birth** |  |  |  |  |  | 1.000 |
| Unplanned preterm birth | 6/430 (1.4) | 11/416 (2.6) | RR 0.53 (0.2, 1.42) | RR 0.53 (0.20, 1.43) | 0.210 |  |
| Hypertensive disorders of pregnancy | 1/32 (3.1) | 0/39 (0.0) | NE | NE | NE |  |
| Rhesus iso-immunization | 0/17 (0.0) | 0/17 (0.0) | NE | NE | NE |  |
| Placenta previa | 0/9 (0.0) | 0/13 (0.0) | NE | NE | NE |  |
| Diabetes mellitus | 0/5 (0.0) | 1/9 (11.1) | NE | NE | NE |  |
| **Age at cardiovascular death, years, median (5^th^, 95^th^ centile)** | | | | | | |
| **Trial protocol** |  | | | | | 1.000 |
| Standard-dose treatment | 33.1 (20.5, 45.8) | 46.9 (46.0, 47.7) | HR 0.94 (0.13, 6.65) | HR 0.86 (0.12, 6.18) | 0.882 |  |
| Double-standard dose treatment | **-** | **-** | NE | NE | NE |  |
| **Multiple Pregnancy** |  |  |  |  |  | 1.000 |
| Singleton | 33.1 (20.5, 45.8) | 46.9 (46.0, 47.7) | HR 1.05 (0.15, 7.42) | HR 1.01 (0.14, 7.21) | 0.992 |  |
| Multiple | - | - | NE | NE | NE |  |
| **Infant sex** |  |  |  |  |  | 0.994 |
| Female | - | 47.7 (47.7, 47.7) | NE | NE | NE |  |
| Male | 33.1 (20.5, 45.8) | 46.0 (46.0, 46.0) | HR 1.87 (0.17, 20.59) | HR 1.85 (0.17, 20.42) | 0.617 |  |
| **Tocolytic used** |  |  |  |  |  | 1.000 |
| Ethanol | - | - | NE | NE | NE |  |
| Salbutamol | 45.8 (45.8, 45.8) | - | NE | NE | NE |  |
| Both | - | - | NE | NE | NE |  |
| None | 20.5 (20.5, 20.5) | 46.9 (46.0, 47.7) | HR 0.57 (0.05, 6.32) | HR 0.53 (0.05, 5.97) | 0.605 |  |
| **Reason for preterm birth** |  |  |  |  |  | 1.000 |
| Unplanned preterm birth | 45.8 (45.8, 45.8) | 46.0 (46.0, 46.0) | HR 0.96 (0.06, 15.36) | HR 0.89 (0.06, 14.33) | 0.936 |  |
| Hypertensive disorders of pregnancy | 20.5 (20.5, 20.5) | - | NE | NE | NE |  |
| Rhesus iso-immunization | - | - | NE | NE | NE |  |
| Placenta previa | - | - | NE | NE | NE |  |
| Diabetes mellitus | - | 47.7 (47.7, 47.7) | NE | NE | NE |  |
| **Cardiovascular death, n/N (%)** | | | | | | |
| **Trial protocol** |  |  |  |  |  | 1.000 |
| Standard-dose treatment | 2/317 (0.6) | 2/300 (0.7) | RR 0.95 (0.13, 6.70) | RR 0.88 (0.12, 6.26) | 0.896 |  |
| Double-standard dose treatment | 0/176 (0) | 0/194 (0) | NE | NE | NE |  |
| **Multiple Pregnancy** |  |  |  |  |  | 1.000 |
| Singleton | 2/520 (0.4) | 2/548 (0.4) | RR 1.05 (0.15, 7.47) | RR 1.06 (0.15, 7.55) | 0.950 |  |
| Multiple | 0/81 (0) | 0/69 (0) | NE | NE | NE |  |
| **Infant sex** |  |  |  |  |  | 0.971 |
| Female | 0/264 (0) | 1/277 (0.4) | NE | NE | NE |  |
| Male | 2/337 (0.6) | 1/340 (0.3) | RR 2.02 (0.18, 22.2) | RR 2.02 (0.18, 22.21) | 0.566 |  |
| **Tocolytic used** |  |  |  |  |  | 0.979 |
| Ethanol | 0/96 (0) | 0/88 (0) | NE | NE | NE |  |
| Salbutamol | 1/318 (0.3) | 0/321 (0) | NE | NE | NE |  |
| Both | 0/15 (0) | 0/13 (0) | NE | NE | NE |  |
| None | 1/172 (0.6) | 2/195 (1.0) | RR 0.57 (0.05, 6.25) | RR 0.56 (0.05, 6.22) | 0.637 |  |
| **Reason for preterm birth** |  |  |  |  |  | 1.000 |
| Unplanned preterm birth | 1/514 (0.2) | 1/522 (0.2) | RR 1.02 (0.06, 16.25) | RR 1.00 (0.06, 15.97) | 0.999 |  |
| Hypertensive disorders of pregnancy | 1/46 (2.2) | 0/49 (0) | NE | NE | NE |  |
| Rhesus iso-immunization | 0/25 (0) | 0/21 (0) | NE | NE | NE |  |
| Placenta previa | 0/11 (0) | 0/14 (0) | NE | NE | NE |  |
| Diabetes mellitus | 0/5 (0) | 1/11 (9.1) | NE | NE | NE |  |
| **Age at first admission for myocardial infarction or coronary revascularization, years, median (5^th^, 95^th^ centile)** | | | | | | |
| **Trial protocol** |  | | | | | 0.262 |
| Standard-dose treatment | 43.3 (41.4, 45.2) | 44.2 (37.6, 47.1) | HR 0.63 (0.10, 3.74) | HR 0.65 (0.11, 3.91) | 0.636 |  |
| Double-standard dose treatment | 44.6 (42.2, 45.1) | 26.4 (26.4, 26.4) | HR 3.32 (0.35, 31.94) | HR 3.40 (0.35, 32.71) | 0.290 |  |
| **Multiple Pregnancy** |  |  |  |  |  | 0.238 |
| Singleton | 44.9 (42.2, 45.2) | 45.7 (44.2, 47.1) | HR 2.09 (0.38, 11.41) | HR 2.15 (0.39, 11.82) | 0.377 |  |
| Multiple | 41.4 (41.4, 41.4) | 32.0 (26.4, 37.6) | HR 0.36 (0.03, 3.97) | HR 0.37 (0.03, 4.05) | 0.413 |  |
| **Infant sex** |  |  |  |  |  | 0.993 |
| Female | - | 40.9 (26.4, 47.1) | NE | NE | NE |  |
| Male | 44.6 (41.4, 45.2) | - | NE | NE | NE |  |
| **Tocolytic used** |  |  |  |  |  | 0.942 |
| Ethanol | - | 47.1 (47.1, 47.1) | NE | NE | NE |  |
| Salbutamol | 45.1 (44.6, 45.2) | 37.6 (37.6, 37.6) | HR 3.04 (0.32, 29.19) | HR 3.07 (0.32, 29.53) | 0.332 |  |
| Both | - | - | NE | NE | NE |  |
| None | 41.8 (41.4, 42.2) | 35.3 (26.4, 44.2) | HR 1.14 (0.16, 8.09) | HR 1.18 (0.17, 8.41) | 0.870 |  |
| **Reason for preterm birth** |  |  |  |  |  | 1.000 |
| Unplanned preterm birth | 44.6 (41.4, 45.2) | 37.6 (26.4, 47.1) | HR 1.61 (0.38, 6.73) | HR 1.67 (0.40, 7.02) | 0.485 |  |
| Hypertensive disorders of pregnancy | - | - | NE | NE | NE |  |
| Rhesus iso-immunization | - | - | NE | NE | NE |  |
| Placenta previa | - | - | NE | NE | NE |  |
| Diabetes mellitus | - | 44.2 (44.2, 44.2) | NE | NE | NE |  |
| **At least one admission for myocardial infarction or coronary revascularization, n/N (%)** | | | | | | |
| **Trial protocol** |  |  |  |  |  | 0.264 |
| Standard-dose treatment | 2/317 (0.6) | 3/300 (1.0) | RR 0.63 (0.11, 3.76) | RR 0.65 (0.11, 3.92) | 0.641 |  |
| Double-standard dose treatment | 3/176 (1.7) | 1/194 (0.5) | RR 3.31 (0.34, 31.73) | RR 3.34 (0.35, 32.36) | 0.290 |  |
| **Multiple Pregnancy** |  |  |  |  |  | 0.241 |
| Singleton | 4/425 (0.9) | 2/444 (0.5) | RR 2.09 (0.38, 11.37) | RR 2.16 (0.39, 11.79) | 0.375 |  |
| Multiple | 1/68 (1.5) | 2/50 (4.0) | RR 0.37 (0.03, 4.04) | RR 0.38 (0.04, 4.05) | 0.420 |  |
| **Infant sex** |  |  |  |  |  | 0.973 |
| Female | 0/209 (0.0) | 4/226 (1.8) | NE | NE | NE |  |
| Male | 5/284 (1.8) | 0/268 (0.0) | NE | NE | NE |  |
| **Tocolytic used** |  |  |  |  |  | 0.940 |
| Ethanol | 0/82 (0.0) | 1/64 (1.6) | NE | NE | NE |  |
| Salbutamol | 3/253 (1.2) | 1/256 (0.4) | RR 3.04 (0.32, 29.15) | RR 3.07 (0.32, 29.45) | 0.330 |  |
| Both | 0/14 (0.0) | 0/10 (0.0) | NE | NE | NE |  |
| None | 2/144 (1.4) | 2/164 (1.2) | RR 1.14 (0.16, 8.04) | RR 1.18 (0.17, 8.31) | 0.871 |  |
| **Reason for preterm birth** |  |  |  |  |  | 1.000 |
| Unplanned preterm birth | 5/430 (1.2) | 3/416 (0.7) | RR 1.61 (0.39, 6.72) | RR 1.68 (0.40, 7.03) | 0.478 |  |
| Hypertensive disorders of pregnancy | 0/32 (0.0) | 0/39 (0.0) | NE | NE | NE |  |
| Rhesus iso-immunization | 0/17 (0.0) | 0/17(0.0) | NE | NE | NE |  |
| Placenta previa | 0/9 (0.0) | 0/13 (0.0) | NE | NE | NE |  |
| Diabetes mellitus | 0/5 (0.0) | 1/9 (11.1) | NE | NE | NE |  |
| **Age at first admission for myocardial infarction, years, median (5^th^, 95^th^ centile)** | | | | | | |
| **Trial protocol** |  |  |  |  |  | 0.993 |
| Standard-dose treatment | 43.3 (41.4, 45.2) | 41.9 (37.6, 46.2) | HR 0.94 (0.13, 6.67) | HR 0.96 (0.13, 6.92) | 0.971 |  |
| Double-standard dose treatment | 43.7 (42.2, 45.1) | - | NE | NE | NE |  |
| **Multiple Pregnancy** |  |  |  |  |  | 0.420 |
| Singleton | 45.1 (42.2, 45.2) | 46.2 (46.2, 46.2) | HR 3.14 (0.33, 30.17) | HR 3.27 (0.34, 31.57) | 0.306 |  |
| Multiple | 41.4 (41.4, 41.4) | 37.6 (37.6, 37.6) | HR 0.73 (0.05, 11.64) | HR 0.75 (0.05, 11.97) | 0.837 |  |
| **Infant sex** |  |  |  |  |  | 0.994 |
| Female | 43.7 (41.4, 45.2) | - | NE | NE | NE |  |
| Male | - | 41.9 (37.6, 46.2) | NE | NE | NE |  |
| **Tocolytic used** |  |  |  |  |  | 1.000 |
| Ethanol | - | - | NE | NE | NE |  |
| Salbutamol | 45.1 (45.1, 45.1) | 37.6 (37.6, 37.6) | HR 2.02 (0.18, 22.29) | HR 2.04 (0.18, 22.52) | 0.561 |  |
| Both | - | - | NE | NE | NE |  |
| None | 41.8 (41.4, 42.2) | 46(46, 46) | HR 2.29 (0.21, 25.29) | HR 2.45 (0.22, 27.18) | 0.465 |  |
| **Reason for preterm birth** |  |  |  |  |  | 1.000 |
| Unplanned preterm birth | 43.7 (41.4, 45.2) | 37.6 (37.6, 37.6) | HR 3.86 (0.43, 34.57) | HR 4.00 (0.44, 35.97) | 0.217 |  |
| Hypertensive disorders of pregnancy | - | - | NE | NE | NE |  |
| Rhesus iso-immunization | - | - | NE | NE | NE |  |
| Placenta previa | - | - | NE | NE | NE |  |
| Diabetes mellitus | - | 46.2 (46.2, 46.2) | NE | NE | NE |  |
| **At least one admission for myocardial infarction, n/N (%)** | | | | | | |
| **Trial protocol** |  |  |  |  |  | 0.974 |
| Standard-dose treatment | 2/317 (0.6) | 2/300 (0.7) | RR 0.95 (0.13, 6.70) | RR 0.97 (0.14, 6.93) | 0.975 |  |
| Double-standard dose treatment | 2/176 (1.1) | 0/194 (0.0) | NE | NE | NE |  |
| **Multiple Pregnancy** |  |  |  |  |  | 0.420 |
| Singleton | 3/425 (0.7) | 1/444 (0.2) | RR 3.13 (0.33, 30.11) | RR 3.27 (0.34, 31.49) | 0.306 |  |
| Multiple | 1/68 (1.5) | 1/50 (2.0) | RR 0.74 (0.05, 11.81) | RR 0.76 (0.05, 11.81) | 0.842 |  |
| **Infant sex** |  |  |  |  |  | 0.974 |
| Female | 0/209 (0.0) | 2/226 (0.9) | NE | NE | NE |  |
| Male | 4/284 (1.4) | 0/268 (0.0) | NE | NE | NE |  |
| **Tocolytic used** |  |  |  |  |  | 1.000 |
| Ethanol | 0/82 (0.0) | 0/64 (0.0) | NE | NE | NE |  |
| Salbutamol | 2/253 (0.8) | 1/256 (0.4) | RR 2.02 (0.18, 22.31) | RR 2.04 (0.19, 22.48) | 0.559 |  |
| Both | 0/14 (0.0) | 0/10 (0.0) | NE | NE | NE |  |
| None | 2/144 (1.4) | 1/164 (0.6) | RR 2.28 (0.21, 25.10) | RR 2.43 (0.22, 26.70) | 0.467 |  |
| **Reason for preterm birth** |  |  |  |  |  | 1.000 |
| Unplanned preterm birth | 4/430 (0.9) | 1/416 (0.2) | RR 3.87 (0.43, 34.59) | RR 4.04 (0.45, 36.28) | 0.213 |  |
| Hypertensive disorders of pregnancy | 0/32 (0.0) | 0/39 (0.0) | NE | NE | NE |  |
| Rhesus iso-immunization | 0/17 (0.0) | 0/17 (0.0) | NE | NE | NE |  |
| Placenta previa | 0/9 (0.0) | 0/13 (0.0) | NE | NE | NE |  |
| Diabetes mellitus | 0/5 (0.0) | 1/9 (11.1) | NE | NE | NE |  |
| **Age at first admission for coronary revascularization, years, median (5^th^, 95^th^ centile)** | | | | | | |
| **Trial protocol** |  |  |  |  |  | 0.995 |
| Standard-dose treatment | - | 44.2 (37.6, 47.1) | NE | NE | NE |  |
| Double-standard dose treatment | 44.6 (44.6, 44.6) | 26.4 (26.4, 26.4) | HR 1.10 (0.07, 17.63) | HR 1.06 (0.07, 16.95) | 0.969 |  |
| **Multiple Pregnancy** |  |  |  |  |  | 0.995 |
| Singleton | 44.6 (44.6, 44.6) | 45.7 (44.2, 47.1) | HR 0.52 (0.05, 5.74) | HR 0.60 (0.05, 6.70) | 0.677 |  |
| Multiple | - | 32.0 (26.4, 37.6) | NE | NE | NE |  |
| **Infant sex** |  |  |  |  |  | 0.995 |
| Female | - | 40.9 (26.4, 47.1) | NE | NE | NE |  |
| Male | 44.6 (44.6, 44.6) | - | NE | NE | NE |  |
| **Tocolytic used** |  |  |  |  |  | 1.000 |
| Ethanol | - | 47.1 (47.1, 47.1) | NE | NE | NE |  |
| Salbutamol | 44.6 (44.6, 44.6) | 37.6 (37.6, 37.6) | HR 1.01 (0.06, 16.15) | HR 1.10 (0.07, 17.72) | 0.947 |  |
| Both | - | - | NE | NE | NE |  |
| None | - | 35.3 (26.4, 44.2) | NE | NE | NE |  |
| **Reason for preterm birth** |  |  |  |  |  | 1.000 |
| Unplanned preterm birth | 44.6 (44.6, 44.6) | 37.6 (26.4, 47.1) | HR 0.32 (0.03, 3.08) | HR 0.36 (0.04, 3.54) | 0.384 |  |
| Hypertensive disorders of pregnancy | - | - | NE | NE | NE |  |
| Rhesus iso-immunization | - | - | NE | NE | NE |  |
| Placenta previa | - | - | NE | NE | NE |  |
| Diabetes mellitus | - | 44.2 (44.2, 44.2) | NE | NE | NE |  |
| **At least one admission for coronary revascularization, n/N (%)** | | | | | | |
| **Trial protocol** |  |  |  |  |  | 0.979 |
| Standard-dose treatment | 0/317 (0.0) | 3/300 (1.0) | NE | NE | NE |  |
| Double-standard dose treatment | 1/176 (0.6) | 1/194 (0.5) | RR 1.10 (0.07, 17.65) | RR 1.05 (0.07, 16.77) | 0.970 |  |
| **Multiple Pregnancy** |  |  |  |  |  | 0.975 |
| Singleton | 1/425 (0.2) | 2/444 (0.5) | RR 0.52 (0.05, 5.76) | RR 0.60 (0.05, 6.71) | 0.678 |  |
| Multiple | 0/68 (0.0) | 2/50 (4.0) | NE | NE | NE |  |
| **Infant sex** |  |  |  |  |  | 0.975 |
| Female | 0/209 (0.0) | 4/226 (1.8) | NE | NE | NE |  |
| Male | 1/284 (0.4) | 0/268 (0.0) | NE | NE | NE |  |
| **Tocolytic used** |  |  |  |  |  | 1.000 |
| Ethanol | 0/82 (0.0) | 1/64 (1.6) | NE | NE | NE |  |
| Salbutamol | 1/253 (0.4) | 1/256 (0.4) | RR 1.02 (0.06, 16.20) | RR 1.10 (0.07, 17.69) | 0.945 |  |
| Both | 0/14 (0.0) | 0/10 (0.0) | NE | NE | NE |  |
| None | 0/144 (0.0) | 2/164 (1.2) | NE | NE | NE |  |
| **Reason for preterm birth** |  |  |  |  |  | 1.000 |
| Unplanned preterm birth | 1/430 (0.2) | 3/416 (0.7) | RR 0.33 (0.03, 3.10) | RR 0.37 (0.04, 3.61) | 0.393 |  |
| Hypertensive disorders of pregnancy | 0/32 (0.0) | 0/39 (0.0) | NE | NE | NE |  |
| Rhesus iso-immunization | 0/17 (0.0) | 0/17 (0.0) | NE | NE | NE |  |
| Placenta previa | 0/9 (0.0) | 0/13 (0.0) | NE | NE | NE |  |
| Diabetes mellitus | 0/5 (0.0) | 1/9 (11.1) | NE | NE | NE |  |
| **Age at first admission for peripheral vascular disease, years, median (5^th^, 95^th^ centile)** | No subgroup analysis was performed as no events occurred in either treatment group | | | | |  |
| **At least one admission for peripheral vascular disease (arterial thrombosis/embolism or need for revascularization), n/N (%)** |  |  |  |  |  |  |
| **Age at first admission for stroke, years, median (5^th^, 95^th^ centile)** | | | | | | |
| **Trial protocol** |  |  |  |  |  | 0.996 |
| Standard-dose treatment | - | 48.9 (47.6, 50.3) | NE | NE | NE |  |
| Double-standard dose treatment | 47.0 (47.0, 47.0) | - | NE | NE | NE |  |
| **Multiple Pregnancy** |  |  |  |  |  | 1.000 |
| Singleton | 47.0 (47.0, 47.0) | 48.9 (47.6, 50.3) | HR 0.52 (0.05, 5.75) | HR 0.54 (0.05, 5.97) | 0.613 |  |
| Multiple | - | - | NE | NE | NE |  |
| **Infant sex** |  |  |  |  |  | 0.995 |
| Female | - | 47.6 (47.6, 47.6) | NE | NE | NE |  |
| Male | 47.0 (47.0, 47.0) | 50.3 (50.3, 50.3) | HR 0.95 (0.06, 15.14) | HR 0.97 (0.06, 15.61) | 0.985 |  |
| **Tocolytic used** |  |  |  |  |  | 1.000 |
| Ethanol | - | - | NE | NE | NE |  |
| Salbutamol | 47.0 (47.0, 47.0) | 48.9 (47.6, 50.3) | HR 0.51 (0.05, 5.59) | HR 0.51 (0.05, 5.63) | 0.582 |  |
| Both | - | - | NE | NE | NE |  |
| None | - | - | NE | NE | NE |  |
| **Reason for preterm birth** |  |  |  |  |  | 1.000 |
| Unplanned preterm birth | 47.0 (47.0, 47.0) | 48.9 (47.6, 50.3) | HR 0.48 (0.04, 5.32) | HR 0.49 (0.04, 5.49) | 0.566 |  |
| Hypertensive disorders of pregnancy | - | - | NE | NE | NE |  |
| Rhesus iso-immunization | - | - | NE | NE | NE |  |
| Placenta previa | - | - | NE | NE | NE |  |
| Diabetes mellitus | - | - | NE | NE | NE |  |
| **At least one admission for stroke, n/N (%)** | | | | | | |
| **Trial protocol** |  |  |  |  |  | 0.975 |
| Standard-dose treatment | 0/317 (0.0) | 2/300 (0.7) | NE | NE | NE |  |
| Double-standard dose treatment | 1/176 (0.6) | 0/194 (0.0) | NE | NE | NE |  |
| **Multiple Pregnancy** |  |  |  |  |  | 1.000 |
| Singleton | 1/425 (0.2) | 2/444 (0.5) | RR 0.52 (0.05, 5.76) | RR 0.54 (0.05, 5.99) | 0.614 |  |
| Multiple | 0/68 (0.0) | 0/50 (0.0) | NE | NE | NE |  |
| **Infant sex** |  |  |  |  |  | 0.976 |
| Female | 0/209 (0.0) | 1/226 (0.4) | NE | NE | NE |  |
| Male | 1/284 (0.0) | 1/268 (0.4) | RR 0.94 (0.06, 15.10) | RR 0.97 (0.06, 15.58) | 0.985 |  |
| **Tocolytic used** |  |  |  |  |  | <0.001 |
| Ethanol | 0/82 (0.0) | 0/64 (0.0) | NE | NE | NE |  |
| Salbutamol | 1/253 (0.4) | 2/256 (0.8) | RR 0.51 (0.05, 5.58) | RR 0.51 (0.05, 5.62) | 0.582 |  |
| Both | 0/14 (0.0) | 0/10 (0.0) | NE | NE | NE |  |
| None | 0/144 (0.0) | 0/164 (0.0) | NE | NE | NE |  |
| **Reason for preterm birth** |  |  |  |  |  | 1.000 |
| Unplanned preterm birth | 1/430 (0.2) | 2/416 (0.5) | RR 0.48 (0.04, 5.33) | RR 0.50 (0.04, 5.52) | 0.568 |  |
| Hypertensive disorders of pregnancy | 0/32 (0.0) | 0/39 (0.0) | NE | NE | NE |  |
| Rhesus iso-immunization | 0/17 (0.0) | 0/17 (0.0) | NE | NE | NE |  |
| Placenta previa | 0/9 (0.0) | 0/13 (0.0) | NE | NE | NE |  |
| Diabetes mellitus | 0/5 (0.0) | 0/9 (0.0) | NE | NE | NE |  |
| **Age at first heart failure admission, years, median (5^th^, 95^th^ centile)** | | | | | | |
| **Trial protocol** |  |  |  |  |  | 0.598 |
| Standard-dose treatment | 42.1 (35.7, 48.5) | 46.7 (40.3, 47.1) | HR 0.63 (0.10, 3.74) | HR 0.61 (0.10, 3.68) | 0.590 |  |
| Double-standard dose treatment | 41.8 (41.8, 41.8) | 33.9 (24.2, 43.8) | HR 0.28 (0.03, 2.46) | HR 0.29 (0.03, 2.55) | 0.262 |  |
| **Multiple Pregnancy** |  |  |  |  |  | 0.991 |
| Singleton | 41.8 (35.7, 48.5) | 45.3 (40.3, 47.1) | HR 0.78 (0.18, 3.49) | HR 0.78 (0.17, 3.48) | 0.741 |  |
| Multiple | - | 31.9 (24.2, 36.0) | NE | NE | NE |  |
| **Infant sex** |  |  |  |  |  | 0.991 |
| Female | - | 40.3 (24.2, 47.1) | NE | NE | NE |  |
| Male | 41.8 (37.7, 48.5) | 39.9 (31.9, 46.7) | HR 0.71 (0.16, 3.16) | HR 0.71 (0.16, 3.18) | 0.656 |  |
| **Tocolytic used** |  |  |  |  |  | 1.000 |
| Ethanol | - | 47.1 (47.1, 47.1) | NE | NE | NE |  |
| Salbutamol | 41.8 (35.7, 48.5) | 45.3 (43.8, 46.7) | HR 1.52 (0.25, 9.11) | HR 1.51 (0.25, 9.03) | 0.653 |  |
| Both | - | 40.3 (40.3, 40.3) | NE | NE | NE |  |
| None | - | 31.9 (24.2, 36.0) | NE | NE | NE |  |
| **Reason for preterm birth** |  |  |  |  |  | 1.000 |
| Unplanned preterm birth | 41.7 (35.7, 48.5) | 40.3 (24.2, 47.1) | HR 0.41 (0.11, 1.59) | HR 0.41 (0.10, 1.57) | 0.192 |  |
| Hypertensive disorders of pregnancy | - | - | NE | NE | NE |  |
| Rhesus iso-immunization | - | - | NE | NE | NE |  |
| Placenta previa | - | - | NE | NE | NE |  |
| Diabetes mellitus | - | - | NE | NE | NE |  |
| **At least one admission for heart failure, n/N (%)** | | | | | | |
| **Trial protocol** |  |  |  |  |  | 0.596 |
| Standard-dose treatment | 2/317 (0.6) | 3/300 (1.0) | RR 0.63 (0.11, 3.76) | RR 0.62 (0.10, 3.69) | 0.595 |  |
| Double-standard dose treatment | 1/176 (0.6) | 4/194 (2.1) | RR 0.28 (0.03, 2.46) | RR 0.29 (0.03, 2.55) | 0.263 |  |
| **Multiple Pregnancy** |  |  |  |  |  | 0.974 |
| Singleton | 3/425 (0.7) | 4/444 (0.9) | RR 0.78 (0.18, 3.49) | RR 0.78 (0.17, 3.47) | 0.743 |  |
| Multiple | 0/68 (0.0) | 3/50 (6.0) | NE | NE | NE |  |
| **Infant sex** |  |  |  |  |  | 0.974 |
| Female | 0/209 (0.0) | 3/226 (1.3) | NE | NE | NE |  |
| Male | 3/284 (1.1) | 4/268(1.5) | RR 0.71 (0.16, 3.14) | RR 0.71 (0.16, 3.16) | 0.656 |  |
| **Tocolytic used** |  |  |  |  |  | 1.000 |
| Ethanol | 0/82 (0.0) | 1/64 (1.6) | NE | NE | NE |  |
| Salbutamol | 3/253 (1.2) | 2/256 (0.8) | RR 1.52 (0.25, 9.05) | RR 1.50 (0.25, 8.93) | 0.654 |  |
| Both | 0/14 (0.0) | 1/10 (10.0) | NE | NE | NE |  |
| None | 0/144 (0.0) | 3/164 (1.8) | NE | NE | NE |  |
| **Reason for preterm birth** |  |  |  |  |  | 1.000 |
| Unplanned preterm birth | 3/430 (0.7) | 7/416 (1.7) | RR 0.41 (0.11, 1.60) | RR 0.41 (0.11, 1.58) | 0.195 |  |
| Hypertensive disorders of pregnancy | 0/32 (0.0) | 0/39 (0.0) | NE | NE | NE |  |
| Rhesus iso-immunization | 0/17 (0.0) | 0/17 (0.0) | NE | NE | NE |  |
| Placenta previa | 0/9 (0.0) | 0/13 (0.0) | NE | NE | NE |  |
| Diabetes mellitus | 0/5 (0.0) | 0/9 (0.0) | NE | NE | NE |  |
| **Time to death after randomization, days, median (5^th^, 95^th^ centile)** | | | | | | |
| **Trial protocol** |  |  |  |  |  | 0.736 |
| Standard-dose treatment | 10.6 (0.3, 12849.6) | 6.1 (0.3, 17401.0) | HR 0.83 (0.63, 1.09) | HR 0.84 (0.64, 1.11) | 0.215 |  |
| Double-standard dose treatment | 6.4 (0.5, 4636.3) | 2.9 (0.3, 16895.0) | HR 0.84 (0.56, 1.24) | HR 0.77 (0.52, 1.15) | 0.202 |  |
| **Multiple Pregnancy** |  |  |  |  |  | 0.591 |
| Singleton | 10.8 (0.4, 12283.1) | 4.1 (0.3, 17401.0) | HR 0.91 (0.71, 1.16) | HR 0.84 (0.66, 1.07) | 0.157 |  |
| Multiple | 9.8 (0.0, 14315.4) | 3.5 (0.0, 16725.0) | HR 0.48 (0.26, 0.90) | HR 0.69 (0.36, 1.33) | 0.271 |  |
| **Infant sex** |  |  |  |  |  | 0.146 |
| Female | 10.5 (0.5, 4374.63) | 5.3 (0.3, 17429.3) | HR 1.02 (0.72, 1.44) | HR 1.00 (0.70, 1.41) | 0.981 |  |
| Male | 10.0 (0.3, 13806.9) | 3.8 (0.3, 16895.0) | HR 0.72 (0.53, 0.97) | HR 0.71 (0.53, 0.96) | 0.025 |  |
| **Tocolytic used** |  |  |  |  |  | 0.045 |
| Ethanol | 8.8 (0.0, 15635.3) | 4.5 (0.8, 15138.1) | HR 0.51 (0.29, 0.90) | HR 0.48 (0.27, 0.84) | 0.011 |  |
| Salbutamol | 10.5 (0.5, 12283.1) | 2.9 (0.3, 15691.9) | HR 0.96 (0.71, 1.31) | HR 1.06 (0.77, 1.45) | 0.739 |  |
| Both | 120.2 (1.4, 239.0) | 18.0 (0.0, 18580.9) | HR 0.32 (0.06, 1.64) | HR 0.25 (0.05, 1.29) | 0.097 |  |
| None | 10.0 (0.3, 14315.4) | 12.2 (0.3, 17807.0) | HR 0.91 (0.60, 1.39) | HR 0.76 (0.50, 1.16) | 0.209 |  |
| **Reason for preterm birth** |  |  |  |  |  | 0.677 |
| Unplanned preterm birth | 10.5 (0.3, 13806.9) | 3.5 (0.3, 16895.0) | HR 0.76 (0.59, 0.98) | HR 0.77 (0.60, 0.99) | 0.045 |  |
| Hypertensive disorders of pregnancy | 11.0 (1.4, 7472.4) | 10.0 (0.3, 6544.0) | HR 1.60 (0.76, 3.35) | HR 1.39 (0.66, 2.92) | 0.383 |  |
| Rhesus iso-immunization | 9.0 (1.0, 62.3) | 5.3 (1.8, 17828.4) | HR 1.64 (0.55, 4.89) | HR 0.92 (0.31, 2.77) | 0.885 |  |
| Placenta previa | 26.0 (7.0, 94.0) | 150.5 (36.0, 7305.3) | HR 1.46 (0.30, 7.25) | HR 1.11 (0.22, 5.52) | 0.898 |  |
| Diabetes mellitus | - | 7492.3 (10.1, 17613.0) | NE | NE | NE |  |
| **Time to MACE after randomization (excluding cardiovascular death), days, median (5^th^, 95 centile)** | | | | | | |
| **Trial protocol** |  |  |  |  |  | 0.468 |
| Standard-dose treatment | 15135 (13132, 17718) | 17075 (13726, 18444) | HR 0.40 (0.10, 1.55) | HR 0.41 (0.10, 1.58) | 0.194 |  |
| Double-standard dose treatment | 15436 (15267, 16302) | 12449 (8874, 16064) | HR 0.82 (0.18, 3.67) | HR 0.86 (0.19, 3.85) | 0.844 |  |
| **Multiple Pregnancy** |  |  |  |  |  | 0.232 |
| Singleton | 15436 (13132, 17718) | 17075 (14746, 18444) | HR 0.74 (0.24, 2.34) | HR 0.76 (0.24, 2.41) | 0.644 |  |
| Multiple | 15135 (15135, 15135) | 12449 (8874, 13727) | HR 0.17 (0.02, 1.52) | HR 0.17 (0.02, 1.51) | 0.112 |  |
| **Infant sex** |  |  |  |  |  | 0.988 |
| Female | - | 15447 (8874, 17376) | NE | NE | NE |  |
| Male | 15351 (13132, 17718) | 16064 (11712, 18444) | HR 1.11 (0.34, 3.65) | HR 1.15 (0.35, 3.78) | 0.818 |  |
| **Tocolytic used** |  |  |  |  |  | 0.994 |
| Ethanol | - | 17233 (17233, 17233) | NE | NE | NE |  |
| Salbutamol | 15784 (13132, 17718) | 17075 (13727, 18444) | HR 0.80 (0.22, 2.98) | HR 0.81 (0.22, 3.01) | 0.751 |  |
| Both | - | 14746 (14746, 14746) | NE | NE | NE |  |
| None | 15286 (15135, 15436) | 12449 (8874, 16147) | HR 0.57 (0.11, 3.12) | HR 0.59 (0.11, 3.26) | 0.549 |  |
| **Reason for preterm birth** |  |  |  |  |  | 1.000 |
| Unplanned preterm birth | 15351 (13132, 17718) | 15405 (8874, 18444) | HR 0.57 (0.21, 1.57) | HR 0.58 (0.21, 1.61) | 0.297 |  |
| Hypertensive disorders of pregnancy | - | - | NE | NE | NE |  |
| Rhesus iso-immunization | - | - | NE | NE | NE |  |
| Placenta previa | - | - | NE | NE | NE |  |
| Diabetes mellitus | - | 16147 (16147, 16147) | NE | NE | NE |  |
| **Number of admissions to hospital with respiratory illness as primary reason for admission, mean (SD)** | | | | | | |
| **Trial protocol** |  |  |  |  |  | 0.209 |
| Standard-dose treatment | 0.2 (0.8) | 0.2 (0.8) | MD -0.1 (-0.3, 0.1) | MD -0.1 (-0.4, 0.3) | 0.749 |  |
| Double-standard dose treatment | 0.1 (0.5) | 0.5 (3.0) | MD -0.4 (-1.0, 0.3) | MD -0.4 (-0.8, 0.0) | 0.082 |  |
| **Multiple Pregnancy** |  |  |  |  |  | 0.837 |
| Singleton | 0.2 (0.7) | 0.4 (2.0) | MD -0.2 (-0.5, 0.1) | MD -0.2 (-0.5, 0.1) | 0.219 |  |
| Multiple | 0.0 (0.2) | 0.1 (0.5) | MD -0.1 (-0.3, 0.1) | MD -0.1 (-0.9, 0.6) | 0.758 |  |
| **Infant sex** |  |  |  |  |  | 0.281 |
| Female | 0.2 (0.5) | 0.5 (2.6) | MD -0.3 (-0.8, 0.2) | MD -0.3 (-0.7, 0.1) | 0.099 |  |
| Male | 0.1 (0.8) | 0.2 (0.6) | MD 0.0 (-0.2, 0.1) | MD 0.0 (-0.4, 0.3) | 0.825 |  |
| **Tocolytic used** |  |  |  |  |  | 0.807 |
| Ethanol | 0.1 (0.4) | 0.2 (0.9) | MD -0.1 (-0.4, 0.2) | MD -0.1 (-0.7, 0.6) | 0.838 |  |
| Salbutamol | 0.2 (0.8) | 0.4 (2.6) | MD -0.3 (-0.8, 0.2) | MD -0.3 (-0.6, 0.1) | 0.168 |  |
| Both | 0.1 (0.4) | 0.8 (1.6) | MD -0.7 (-2.1, 0.7) | MD -0.7 (-2.2, 0.8) | 0.364 |  |
| None | 0.2 (0.5) | 0.2 (0.7) | MD -0.1 (-0.3, 0.1) | MD -0.1 (-0.5, 0.4) | 0.828 |  |
| **Reason for preterm birth** |  |  |  |  |  | 0.971 |
| Unplanned preterm birth | 0.2 (0.7) | 0.4 (2.1) | MD -0.2 (-0.5, 0.1) | MD -0.2 (-0.5, 0.1) | 0.170 |  |
| Hypertensive disorders of pregnancy | 0.2 (0.4) | 0.2 (0.7) | MD 0.0 (-0.3, 0.3) | MD 0.0 (-0.8, 0.8) | 0.999 |  |
| Rhesus iso-immunization | 0.0 (0.0) | 0.0 (0.0) | NE | NE | NE |  |
| Placenta previa | 0.0 (0.0) | 0.0 (0.0) | NE | NE | NE |  |
| Diabetes mellitus | 0.0 (0.0) | 0.7 (1.2) | MD -0.7 (-2.9, 1.5) | MD -0.7 (-2.9, 1.6) | 0.557 |  |
| **Self-reported general health fair/poor, n/N (%)** | | | | | | |
| **Trial protocol** |  |  |  |  |  | 0.647 |
| Standard-dose treatment | 24/141 (17.0) | 14/120 (11.7) | RR 1.46 (0.79, 2.70) | RR 1.46 (0.79, 2.71) | 0.228 |  |
| Double-standard dose treatment | 13/82 (15.9) | 10/71 (14.1) | RR 1.13 (0.52, 2.42) | RR 1.16 (0.54, 2.50) | 0.699 |  |
| **Multiple Pregnancy** |  |  |  |  |  | 0.598 |
| Singleton | 32/189 (16.9) | 21/172 (12.2) | RR 1.39 (0.83, 2.31) | RR 1.40 (0.84, 2.34) | 0.195 |  |
| Multiple | 5/34 (14.7) | 3/19 (15.8) | RR 0.93 (0.24, 3.59) | RR 0.96 (0.25, 3.61) | 0.950 |  |
| **Infant sex** |  |  |  |  |  | 0.550 |
| Female | 18/103 (17.5) | 12/104 (11.5) | RR 1.51 (0.77, 2.99) | RR 1.54 (0.78, 3.05) | 0.210 |  |
| Male | 19/120 (15.8) | 12/87 (13.8) | RR 1.15 (0.59, 2.25) | RR 1.16 (0.59, 2.26) | 0.672 |  |
| **Tocolytic used** |  |  |  |  |  | 0.469 |
| Ethanol | 4/38 (10.5) | 1/28 (3.6) | RR 2.95 (0.33, 26.01) | RR 2.96 (0.35, 25.25) | 0.320 |  |
| Salbutamol | 22/108 (20.4) | 12/94 (12.8) | RR 1.60 (0.83, 3.06) | RR 1.60 (0.84, 3.07) | 0.154 |  |
| Both | 2/7 (28.6) | 0/4 (0.0) | NE | NE | NE |  |
| None | 9/70 (12.9) | 11/65 (16.9) | RR 0.76 (0.33, 1.73) | RR 0.77 (0.34, 1.75) | 0.534 |  |
| **Reason for preterm birth** |  |  |  |  |  | 0.987 |
| Unplanned preterm birth | 32/187 (17.1) | 21/157 (13.4) | RR 1.28 (0.77, 2.13) | RR 1.29 (0.77, 2.16) | 0.326 |  |
| Hypertensive disorders of pregnancy | 4/20 (20.0) | 2/19 (10.5) | RR 1.90 (0.37, 9.70) | RR 1.92 (0.40, 9.35) | 0.416 |  |
| Rhesus iso-immunization | 1/10 (10.0) | 1/8 (12.5) | RR 0.80 (0.05, 13.48) | RR 0.83 (0.06, 11.46) | 0.887 |  |
| Placenta previa | 0/4 (0.0) | 0/4 (0.0) | NE | NE | NE |  |
| Diabetes mellitus | 0/2 (0.0) | 0/3 (0.0) | NE | NE | NE |  |
| **Time in Hospital after 1988, days per 10 years alive, median (5^th^, 95^th^ centile)** | | | | | | |
| **Trial protocol** |  |  |  |  |  | 0.690 |
| Standard-dose treatment | 1.6 (0.0, 31.1) | 3.2 (0.0, 21.3) | MD -8.0 (-21.1, 5.0) | MD -8.0 (-18.8, 2.9) | 0.149 |  |
| Double-standard dose treatment | 2.3 (0.0, 13.5) | 2.3 (0.0, 51.8) | MD -4.0 (-10.1, 2.1) | MD -3.7 (-18.1, 10.7) | 0.614 |  |
| **Multiple Pregnancy** |  |  |  |  |  | 0.883 |
| Singleton | 2.0 (0.0, 23.0) | 2.4 (0.0, 31.6) | MD -6.5 (-16.3, 3.2) | MD -6.4 (-15.7, 2.8) | 0.171 |  |
| Multiple | 1.1 (0.0, 21.6) | 4.0 (0.0, 30.4) | MD -5.2 (-11.5, 1.1) | MD -4.6 (-29.8, 20.6) | 0.720 |  |
| **Infant sex** |  |  |  |  |  | 0.934 |
| Female | 3.2 (0.0, 25.0) | 3.4 (0.0, 31.6) | MD -6.0 (-21.0, 9.1) | MD -6.0 (-18.5, 6.4) | 0.339 |  |
| Male | 0.9 (0.0, 18.4) | 1.1 (0.0, 38.8) | MD -6.5 (-15.6, 2.6) | MD -6.5 (-18.6, 5.6) | 0.293 |  |
| **Tocolytic used** |  |  |  |  |  | 0.439 |
| Ethanol | 0.9 (0.0, 31.1) | 1.6 (0.0, 20.1) | MD -1.2 (-5.2, 2.9) | MD -0.8 (-23.1, 21.4) | 0.941 |  |
| Salbutamol | 1.7 (0.0, 19.5) | 2.9 (0.0, 24.4) | MD -1.0 (-6.3, 4.4) | MD -0.8 (-13.2, 11.5) | 0.893 |  |
| Both | 1.1 (0.0, 6.9) | 5.7 (0.0, 21.2) | MD -5.1 (-12.0, 1.8) | MD -5.0 (-55.5, 45.6) | 0.847 |  |
| None | 2.4 (0.0, 39.3) | 2.2 (0.0, 82.9) | MD -16.5 (-41.2, 8.3) | MD -16.3 (-31.2,-1.3) | 0.033 |  |
| **Reason for preterm birth** |  |  |  |  |  | 0.004 |
| Unplanned preterm birth | 1.9 (0.0, 23.0) | 3.0 (0.0, 24.4) | MD 0.2 (-3.7, 4.0) | MD 0.4 (-8.7, 9.6) | 0.925 |  |
| Hypertensive disorders of pregnancy | 3.6 (0.0, 10.5) | 1.4 (0.0, 38.8) | MD -35.7 (-108.8, 37.3) | MD -35.4 (-62.1, -8.7) | 0.010 |  |
| Rhesus iso-immunization | 0.7 (0.0, 11.2) | 0.3 (0.3, 18.4) | MD -1.0 (-5.9, 3.9) | MD 1.0 (-39.3, 41.4) | 0.960 |  |
| Placenta previa | 2.3 (0.0, 3.2) | 2.6 (0.0, 3.2) | MD 0.1 (-2.6, 2.8) | MD 1.2 (-62.8, 65.1) | 0.972 |  |
| Diabetes mellitus | 4.6 (0.6, 8.6) | 51.2 (1.1, 469.3) | MD -109.8 (-436.6, 217.0) | MD -110.4 (-181.9, -39.0) | 0.003 |  |
| **Ischemic heart disease (combined hierarchical outcome), n/N (%)** | | | | | | |
| **Trial protocol** |  |  |  |  |  | 0.767 |
| Standard-dose treatment | 2/145 (1.4) | 4/123 (3.3) | RR 0.42 (0.08, 2.29) | RR 0.40 (0.07, 2.15) | 0.284 |  |
| Double-standard dose treatment | 3/84 (3.6) | 5/72 (6.9) | RR 0.51 (0.13, 2.10) | RR 0.55 (0.14, 2.22) | 0.403 |  |
| **Multiple Pregnancy** |  |  |  |  |  | 0.220 |
| Singleton | 4/195 (2.1) | 5/175 (2.9) | RR 0.72 (0.20, 2.64) | RR 0.70 (0.19, 2.57) | 0.587 |  |
| Multiple | 1/34 (2.9) | 4/20 (20.0) | RR 0.15 (0.02, 1.29) | RR 0.15 (0.02, 1.23) | 0.077 |  |
| **Infant sex** |  |  |  |  |  | 0.826 |
| Female | 1/105 (1.0) | 3/107 (2.8) | RR 0.34 (0.04, 3.26) | RR 0.37 (0.04, 3.56) | 0.390 |  |
| Male | 4/124 (3.2) | 6/88 (6.8) | RR 0.47 (0.14, 1.64) | RR 0.50 (0.14, 1.71) | 0.268 |  |
| **Tocolytic used** |  |  |  |  |  | 0.981 |
| Ethanol | 0/38 (0.0) | 1/29 (3.5) | NE | NE | NE |  |
| Salbutamol | 2/111 (1.8) | 4/96 (4.2) | RR 0.43 (0.08, 2.33) | RR 0.41 (0.08, 2.20) | 0.299 |  |
| Both | 0/7 (0.0) | 0/5 (0.0) | NE | NE | NE |  |
| None | 3/73 (4.1) | 4/65 (6.2) | RR 0.67 (0.15, 2.91) | RR 0.66 (0.15, 2.84) | 0.579 |  |
| **Reason for preterm birth** |  |  |  |  |  | 1.000 |
| Unplanned preterm birth | 4/193 (2.1) | 8/161 (5.0) | RR 0.42 (0.13, 1.37) | RR 0.41 (0.13, 1.35) | 0.142 |  |
| Hypertensive disorders of pregnancy | 1/20 (5.0) | 0/19 (0.0) | NE | NE | NE |  |
| Rhesus iso-immunization | 0/10 (0.0) | 0/8 (0.0) | NE | NE | NE |  |
| Placenta previa | 0/4 (0.0) | 0/4 (0.0) | NE | NE | NE |  |
| Diabetes mellitus | 0/2 (0.0) | 1/3 (33.3) | NE | NE | NE |  |
| **Stroke (combined hierarchical outcome), n/N (%)^1^** | | | | | | |
| **Trial protocol** |  |  |  |  |  | 0.973 |
| Standard-dose treatment | 0/145 (0.0) | 3/123 (0.0) | NE | NE | NE |  |
| Double-standard dose treatment | 1/84 (1.2) | 0/72 (0.0) | NE | NE | NE |  |
| **Multiple Pregnancy** |  |  |  |  |  | 0.999 |
| Singleton | 1/195 (0.5) | 3/175 (1.7) | RR 0.30 (0.03, 2.87) | RR 0.32 (0.03, 3.10) | 0.325 |  |
| Multiple | 0/34 (0.0) | 0/20 (0.0) | NE | NE | NE |  |
| **Infant sex** |  |  |  |  |  | 0.971 |
| Female | 0/105 (0.0) | 2/107 (1.9) | NE | NE | NE |  |
| Male | 1/124 (0.8) | 1/88 (1.1) | RR 0.71 (0.04, 11.37) | RR 0.75 (0.05, 12.03) | 0.841 |  |
| **Tocolytic used** |  |  |  |  |  | 1.000 |
| Ethanol | 0/38 (0.0) | 0/29 (0.0) | NE | NE | NE |  |
| Salbutamol | 1/111 (0.9) | 3/96 (3.1) | RR 0.29 (0.03, 2.76) | RR 0.30 (0.03, 2.83) | 0.289 |  |
| Both | 0/7 (0) | 0/5 (0.0) | NE | NE | NE |  |
| None | 0/73 (0.0) | 0/65 (0.0) | NE | NE | NE |  |
| **Reason for preterm birth** |  |  |  |  |  | 1.000 |
| Unplanned preterm birth | 1/193 (0.5) | 3/161 (1.9) | RR 0.28 (0.03, 2.67) | RR 0.30 (0.03, 2.93) | 0.300 |  |
| Hypertensive disorders of pregnancy | 0/20 (0.0) | 0/19 (0.0) | NE | NE | NE |  |
| Rhesus iso-immunization | 0/10 (0.0) | 0/8 (0.0) | NE | NE | NE |  |
| Placenta previa | 0/4 (0.0) | 0/4 (0.0) | NE | NE | NE |  |
| Diabetes mellitus | 0/2 (0.0) | 0/3 (0.0) | NE | NE | NE |  |
| **Peripheral vascular disease, n/N (%)** | No subgroup analysis was performed as no events occurred in either treatment group | | | | | |
| **Weight, kg, mean (SD)** | | | | | | |
| **Trial protocol** |  |  |  |  |  | 0.999 |
| Standard-dose treatment | 88.9 (21.4) | 84.3 (20.7) | MD 4.6 (-0.8, 10.0) | MD 4.7 (-0.3, 9.6) | 0.064 |  |
| Double-standard dose treatment | 88.7 (21.0) | 85.8 (18.7) | MD 2.9 (-3.7, 9.5) | MD 2.9 (-3.4, 9.3) | 0.366 |  |
| **Multiple Pregnancy** |  |  |  |  |  | 0.007 |
| Singleton | 89.2 (21.8) | 83.9 (19.5) | MD 5.4 (0.9, 9.8) | MD 5.6 (1.5, 9.7) | 0.008 |  |
| Multiple | 86.3 (17.5) | 94.0 (22.6) | MD -7.7 (-19.6, 4.2) | MD -9.2 (-20.7, 2.3) | 0.117 |  |
| **Infant sex** |  |  |  |  |  | 0.776 |
| Female | 80.7 (18.9) | 77.5 (18.4) | MD 3.2 (-2.2, 8.7) | MD 3.2 (-2.4, 8.8) | 0.261 |  |
| Male | 95.4 (20.7) | 93.3 (18.3) | MD 2.1 (-3.5, 7.8) | MD 2.1 (-3.6, 7.6) | 0.445 |  |
| **Tocolytic used** |  |  |  |  |  | 0.067 |
| Ethanol | 89.9 (14.8) | 89.3 (23.3) | MD 0.6 (-9.3, 10.5) | MD 0.6 (-9.2, 10.4) | 0.902 |  |
| Salbutamol | 89.4 (23.8) | 85.1 (20.9) | MD 4.3 (-2.2, 10.8) | MD 4.3 (-1.2, 9.8) | 0.129 |  |
| Both | 73.7 (12.2) | 94.5 (11.7) | MD -20.8 (-37.9, -3.7) | MD -20.0 (-43.7, 3.6) | 0.096 |  |
| None | 89.0 (20.1) | 81.8 (16.9) | MD 7.2 (0.5, 13.9) | MD 7.3 (0.5, 14.2) | 0.036 |  |
| **Reason for preterm birth** |  |  |  |  |  | 0.631 |
| Unplanned preterm birth | 88.8 (21.6) | 85.3 (21.0) | MD 3.5 (-1.2, 8.2) | MD 3.6 (-0.7, 7.9) | 0.103 |  |
| Hypertensive disorders of pregnancy | 81.8 (17.5) | 1.8 (15.9) | MD -0.1 (-11.2, 11.1) | MD 0.3 (-12.2, 12.8) | 0.962 |  |
| Rhesus iso-immunization | 99.3 (17.5) | 86.0 (13.2) | MD 13.3 (-3.5, 30.1) | MD 14.1 (-4.6, 32.8) | 0.139 |  |
| Placenta previa | 85.3 (12.7) | 82.0 (3.5) | MD 3.3 (-17.8, 24.5) | MD 1.8 (-29.2, 32.8) | 0.907 |  |
| Diabetes mellitus | 109.1 (23.9) | 84.0 (26.9) | MD 25.1 (-84.3, 134.5) | MD 25.2 (-12.8, 63.1) | 0.193 |  |
| **Height, centimetres, mean (SD)** | | | | | | |
| **Trial protocol** |  |  |  |  |  | 0.175 |
| Standard-dose treatment | 172.2 (10.2) | 170.0 (10.6) | MD 2.2 (-0.5, 4.9) | MD 2.3 (0.4, 4.3) | 0.021 |  |
| Double-standard dose treatment | 173.8 (9.5) | 170.6 (9.8) | MD 3.2 (-0.1, 6.5) | MD 3.1 (0.6, 5.7) | 0.016 |  |
| **Multiple Pregnancy** |  |  |  |  |  | 0.895 |
| Singleton | 172.7 (9.8) | 170.6 (10.3) | MD 2.1 (0.0, 4.3) | MD 2.4 (0.8, 4.1) | 0.004 |  |
| Multiple | 173.2 (10.9) | 166.9 (10.4) | MD 6.3 (-0.6, 13.2) | MD 4.2 (-0.5, 8.9) | 0.079 |  |
| **Infant sex** |  |  |  |  |  | 0.555 |
| Female | 165.2 (6.4) | 164.3 (8.3) | MD 0.9 (-1.2, 3.1) | MD 1.0 (-1.2, 3.2) | 0.382 |  |
| Male | 179.1 (7.8) | 177.3 (7.6) | MD 1.8 (-0.5, 4.0) | MD 1.8 (-0.4, 4.0) | 0.105 |  |
| **Tocolytic used** |  |  |  |  |  | 0.083 |
| Ethanol | 172.1 (10.7) | 173.3 (8.7) | MD -1.2 (-6.5, 4.0) | MD -1.0 (-4.9, 2.9) | 0.631 |  |
| Salbutamol | 173.8 (9.7) | 169.3 (11.4) | MD 4.4 (1.4, 7.5) | MD 4.1 (1.9, 6.3) | <0.001 |  |
| Both | 170.2 (8.8) | 169.0 (5.9) | MD 1.2 (-10.6, 12.9) | MD 2.7 (-6.9, 12.2) | 0.581 |  |
| None | 171.8 (10.1) | 170.3 (9.3) | MD 1.5 (-2.1, 5.1) | MD 2.0 (-0.8, 4.7) | 0.155 |  |
| **Reason for preterm birth** |  |  |  |  |  | 0.130 |
| Unplanned preterm birth | 172.9 (10.3) | 170.2 (10.4) | MD 2.7 (0.4, 5.1) | MD 2.8 (1.1, 4.5) | 0.001 |  |
| Hypertensive disorders of pregnancy | 168.1 (6.6) | 170.3 (10.5) | MD -2.2 (-8.2, 3.7) | MD -1.6 (-6.5, 3.3) | 0.515 |  |
| Rhesus iso-immunization | 177.7 (6.8) | 172.7 (7.8) | MD 5.0 (-2.6, 12.6) | MD 5.7 (-1.5, 13.0) | 0.121 |  |
| Placenta previa | 180.0 (5.2) | 173.3 (5.0) | MD 6.7 (-4.9, 18.3) | MD 5.8 (-6.2, 17.8) | 0.340 |  |
| Diabetes mellitus | 165.0 (NE) | 152.0 (NE) | NE | MD 20.4 (-0.4, 41.3) | 0.054 |  |
| **Body mass index, kg/m^2^, mean (SD)** | | | | | | |
| **Trial protocol** |  |  |  |  |  | 0.485 |
| Standard-dose treatment | 29.8 (6.7) | 28.7 (5.8) | MD 1.1 (-0.6, 2.8) | MD 1.1 (-0.6, 2.7) | 0.216 |  |
| Double-standard dose treatment | 29.5 (6.0) | 29.4 (6.8) | MD 0.0 (-2.1, 2.2) | MD 0.0 (-2.2, 2.1) | 0.979 |  |
| **Multiple Pregnancy** |  |  |  |  |  | 0.003 |
| Singleton | 29.9 (6.7) | 28.5 (5.7) | MD 1.3 (-0.1, 2.7) | MD 1.3 (-0.1, 2.7) | 0.066 |  |
| Multiple | 28.6 (4.7) | 33.5 (8.3) | MD -4.9 (-8.8, -1.0) | MD -5.0 (-8.8, -1.1) | 0.013 |  |
| **Infant sex** |  |  |  |  |  | 0.86 |
| Female | 29.5 (6.8) | 28.7 (6.7) | MD 0.8 (-1.3, 2.8) | MD 0.7 (-1.2, 2.6) | 0.472 |  |
| Male | 29.9 (6.1) | 29.4 (5.5) | MD 0.5 (-1.2, 2.3) | MD 0.5 (-1.4, 2.4) | 0.594 |  |
| **Tocolytic used** |  |  |  |  |  | 0.087 |
| Ethanol | 30.5 (5.2) | 29.3 (6.6) | MD 1.2 (-1.9, 4.3) | MD 1.1 (-2.2, 4.4) | 0.498 |  |
| Salbutamol | 29.6 (7.1) | 29.4 (6.7) | MD 0.2 (-1.9, 2.2) | MD 0.1 (-1.8, 2.0) | 0.912 |  |
| Both | 24.4 (2.3) | 33.1 (3.4) | MD -8.6 (-12.7, -4.5) | MD -8.5 (-16.5, -0.5) | 0.038 |  |
| None | 29.9 (6.0) | 27.9 (5.2) | MD 2.0 (-0.1, 4.1) | MD 2.0 (-0.4, 4.3) | 0.096 |  |
| **Reason for preterm birth** |  |  |  |  |  | 0.387 |
| Unplanned preterm birth | 29.6 (6.5) | 29.1 (6.4) | MD 0.5 (-1.0, 1.9) | MD 0.4 (-1.1, 1.8) | 0.590 |  |
| Hypertensive disorders of pregnancy | 29.3 (5.6) | 28.3 (4.8) | MD 1.0 (-2.6, 4.6) | MD 1.1 (-3.1, 5.3) | 0.609 |  |
| Rhesus iso-immunization | 31.3 (4.5) | 29.2 (6.4) | MD 2.1 (-3.5, 7.7) | MD 2.2 (-3.9, 8.3) | 0.488 |  |
| Placenta previa | 26.2 (2.3) | 27.3 (1.0) | MD -1.1 (-5.1, 3.0) | MD -1.3 (-11.4, 8.8) | 0.799 |  |
| Diabetes mellitus | 46.3 (NE) | 28.1 (NE) | NE | MD 18.1 (0.5, 35.6) | 0.043 |  |
| **Overweight or obesity, n/N (%)** | | | | | | |
| **Trial protocol** |  |  |  |  |  | 0.809 |
| Standard-dose treatment | 95/124 (76.6) | 68/98 (69.4) | RR 1.10 (0.94, 1.30) | RR 1.07 (0.90, 1.26) | 0.449 |  |
| Double-standard dose treatment | 57/74 (77.0) | 46/62 (74.2) | RR 1.04 (0.85, 1.26) | RR 1.03 (0.85, 1.25) | 0.727 |  |
| **Multiple Pregnancy** |  |  |  |  |  | 0.092 |
| Singleton | 129/168 (76.8) | 101/145 (69.7) | RR 1.10 (0.96, 1.26) | RR 1.07 (0.93, 1.23) | 0.319 |  |
| Multiple | 23/30 (76.7) | 13/15 (86.7) | RR 0.88 (0.66, 1.18) | RR 0.81 (0.60, 1.10) | 0.181 |  |
| **Infant sex** |  |  |  |  |  | 0.119 |
| Female | 66/87 (75.9) | 55/86 (64.0) | RR 1.19 (0.97, 1.45) | RR 1.19 (0.97, 1.45) | 0.092 |  |
| Male | 86/111 (77.5) | 59/74 (79.7) | RR 0.97 (0.83, 1.13) | RR 0.97 (0.83, 1.13) | 0.711 |  |
| **Tocolytic used** |  |  |  |  |  | 0.176 |
| Ethanol | 28/34 (82.4) | 16/24 (66.7) | RR 1.24 (0.89, 1.72) | RR 1.20 (0.86, 1.66) | 0.290 |  |
| Salbutamol | 72/98 (73.5) | 60/80 (75.0) | RR 0.98 (0.82, 1.17) | RR 0.96 (0.80, 1.14) | 0.640 |  |
| Both | 2/6 (33.3) | 4/4 (100.0) | RR 0.49 (NE) | RR 0.32 (0.25, 0.40) | <0.001 |  |
| None | 50/60 (83.3) | 34/52 (65.4) | RR 1.27 (1.01, 1.60) | RR 1.25 (0.99, 1.57) | 0.062 |  |
| **Reason for preterm birth** |  |  |  |  |  | 0.591 |
| Unplanned preterm birth | 126/167 (75.5) | 91/131 (69.5) | RR 1.09 (0.94, 1.25) | RR 1.07 (0.92, 1.23) | 0.391 |  |
| Hypertensive disorders of pregnancy | 13/17 (76.5) | 14/18 (77.8) | RR 0.98 (0.68, 1.43) | RR 0.96 (0.67, 1.38) | 0.820 |  |
| Rhesus iso-immunization | 10/10 (100.0) | 5/7 (71.4) | RR 1.33 (NE) | RR 1.35 (1.17, 1.57) | <0.001 |  |
| Placenta previa | 2/3 (66.7) | 3/3 (100.0) | RR 0.73 (NE) | RR 0.61 (0.52, 0.70) | <0.001 |  |
| Diabetes mellitus | 1/1 (100.0) | 1/1 (100.0) | RR 1.00 (NE) | RR 1.11 (0.96, 1.28) | 0.167 |  |
| **Overweight, n/N (%)** | | | | | | |
| **Trial protocol** |  |  |  |  |  | 0.956 |
| Standard-dose treatment | 44/124 (35.5) | 35/98 (35.7) | RR 0.99 (0.69, 1.42) | RR 0.94 (0.66, 1.34) | 0.735 |  |
| Double-standard dose treatment | 29/74 (39.2) | 24/62 (38.7) | RR 1.01 (0.66, 1.55) | RR 0.96 (0.63, 1.46) | 0.832 |  |
| **Multiple Pregnancy** |  |  |  |  |  | 0.416 |
| Singleton | 60/168 (35.7) | 55/145 (37.9) | RR 0.94 (0.70, 1.26) | RR 0.91 (0.68, 1.21) | 0.516 |  |
| Multiple | 13/30 (43.3) | 4/15 (26.7) | RR 1.63 (0.62, 4.25) | RR 1.37 (0.53, 3.54) | 0.518 |  |
| **Infant sex** |  |  |  |  |  | 0.028 |
| Female | 34/87 (39.1) | 24/86 (27.9) | RR 1.40 (0.91, 2.16) | RR 1.36 (0.89, 2.10) | 0.156 |  |
| Male | 39/111 (35.1) | 35/74 (47.3) | RR 0.74 (0.52, 1.06) | RR 0.74 (0.52, 1.04) | 0.081 |  |
| **Tocolytic used** |  |  |  |  |  | 0.851 |
| Ethanol | 10/34 (29.4) | 8/24 (33.3) | RR 0.88 (0.40, 1.94) | RR 0.87 (0.41, 1.88) | 0.727 |  |
| Salbutamol | 36/98 (36.7) | 33/80 (41.3) | RR 0.89 (0.61, 1.29) | RR 0.86 (0.59, 1.23) | 0.403 |  |
| Both | 2/6 (33.3) | 0/4 (0.0) | NE | NE | NE |  |
| None | 25/60 (41.7) | 18/52 (34.6) | RR 1.20 (0.74, 1.95) | RR 1.12 (0.69, 1.81) | 0.651 |  |
| **Reason for preterm birth** |  |  |  |  |  | 0.936 |
| Unplanned preterm birth | 60/167 (35.9) | 45/131 (34.4) | RR 1.05 (0.77, 1.43) | RR 1.00 (0.73, 1.36) | 0.995 |  |
| Hypertensive disorders of pregnancy | 7/17 (41.2) | 8/18 (44.4) | RR 0.93 (0.42, 2.06) | RR 0.90 (0.42, 1.91) | 0.779 |  |
| Rhesus iso-immunization | 4/10 (40.0) | 2/7 (28.6) | RR 1.40 (0.31, 6.39) | RR 1.19 (0.29, 4.89) | 0.812 |  |
| Placenta previa | 2/3 (66.7) | 3/3 (100.0) | RR 0.73 (NE) | RR 0.59 (0.43, 0.81) | 0.001 |  |
| Diabetes mellitus | 0/1 (0.0) | 1/1 (100.0) | NE | NE | NE |  |
| **Obesity, n/N (%)** | | | | | | |
| **Trial protocol** |  |  |  |  |  | 0.746 |
| Standard-dose treatment | 51/124 (41.1) | 33/98 (33.7) | RR 1.22 (0.86, 1.73) | RR 1.20 (0.85, 1.70) | 0.300 |  |
| Double-standard dose treatment | 28/74 (37.8) | 22/62 (35.5) | RR 1.07 (0.68, 1.67) | RR 1.10 (0.70, 1.71) | 0.688 |  |
| **Multiple Pregnancy** |  |  |  |  |  | 0.043 |
| Singleton | 69/168 (41.1) | 46/145 (31.7) | RR 1.29 (0.96, 1.75) | RR 1.28 (0.95, 1.72) | 0.108 |  |
| Multiple | 10/30 (33.3) | 9/15 (60.0) | RR 0.56 (0.28, 1.09) | RR 0.60 (0.31, 1.17) | 0.134 |  |
| **Infant sex** |  |  |  |  |  | 0.354 |
| Female | 32/87 (36.8) | 31/86 (36.1) | RR 1.02 (0.69, 1.52) | RR 1.02 (0.69, 1.50) | 0.941 |  |
| Male | 47/111 (42.3) | 24/74 (32.4) | RR 1.31 (0.88, 1.94) | RR 1.32 (0.89, 1.95) | 0.167 |  |
| **Tocolytic used** |  |  |  |  |  | 0.637 |
| Ethanol | 18/34 (52.9) | 8/24 (33.3) | RR 1.59 (0.82, 3.08) | RR 1.54 (0.80, 2.95) | 0.193 |  |
| Salbutamol | 36/98 (36.7) | 27/80 (33.8) | RR 1.09 (0.73, 1.63) | RR 1.09 (0.73, 1.63) | 0.679 |  |
| Both | 0/6 (0.0) | 4/4 (100.0) | NE | NE | NE |  |
| None | 25/60 (41.7) | 16/52 (30.8) | RR 1.35 (0.81, 2.26) | RR 1.32 (0.80, 2.19) | 0.272 |  |
| **Reason for preterm birth** |  |  |  |  |  | 0.965 |
| Unplanned preterm birth | 66/167 (39.5) | 46/131 (35.1) | RR 1.13 (0.83, 1.52) | RR 1.11 (0.82, 1.49) | 0.499 |  |
| Hypertensive disorders of pregnancy | 6/17 (35.3) | 6/18 (33.3) | RR 1.06 (0.41, 2.74) | RR 1.16 (0.46, 2.91) | 0.748 |  |
| Rhesus iso-immunization | 6/10 (60.0) | 3/7 (42.9) | 1.40 (0.48, 4.13) | RR 1.46 (0.54, 3.94) | 0.458 |  |
| Placenta previa | 0/3 (0.0) | 0/3 (0.0) | NE | NE | NE |  |
| Diabetes mellitus | 1/1 (100.0) | 0/1 (0.0) | NE | NE | NE |  |
| **Death from any cause, n/N (%)** | | | | | | |
| **Trial protocol** |  |  |  |  |  | 0.644 |
| Standard-dose treatment | 96/390 (24.6) | 109/379 (28.8) | RR 0.86 (0.68, 1.08) | RR 0.92 (0.73, 1.16) | 0.481 |  |
| Double-standard dose treatment | 43/211 (20.4) | 56/238 (23.5) | RR 0.87 (0.61, 1.23) | RR 0.84 (0.60, 1.17) | 0.301 |  |
| **Multiple Pregnancy** |  |  |  |  |  | 0.835 |
| Singleton | 123/520 (23.7) | 140/548 (25.6) | RR 0.93 (0.75, 1.14) | RR 0.9 (0.74, 1.09) | 0.287 |  |
| Multiple | 16/81 (19.8) | 25/69 (36.2) | RR 0.55 (0.32, 0.94) | RR 0.84 (0.43, 1.63) | 0.599 |  |
| **Infant sex** |  |  |  |  |  | 0.224 |
| Female | 64/264 (24.2) | 66/277 (23.8) | RR 1.02 (0.75, 1.37) | RR 1.03 (0.76, 1.40) | 0.855 |  |
| Male | 75/337 (22.3) | 99/340 (29.1) | RR 0.76 (0.59, 0.99) | RR 0.80 (0.63, 1.03) | 0.079 |  |
| **Tocolytic used** |  |  |  |  |  | 0.047 |
| Ethanol | 19/96 (19.8) | 31/88 (35.2) | RR 0.56 (0.34, 0.92) | RR 0.58 (0.36, 0.93) | 0.024 |  |
| Salbutamol | 79/318 (24.8) | 81/321 (25.2) | RR 0.98 (0.75, 1.29) | RR 1.09 (0.83, 1.41) | 0.540 |  |
| Both | 2/15 (13.3) | 5/13 (38.5) | RR 0.35 (0.07, 1.61) | RR 0.28 (0.07, 1.16) | 0.078 |  |
| None | 39/172 (22.7) | 48/195 (24.6) | RR 0.92 (0.64, 1.33) | RR 0.85 (0.61, 1.18) | 0.329 |  |
| **Reason for preterm birth** |  |  |  |  |  | 0.808 |
| Unplanned preterm birth | 110/514 (21.4) | 140/522 (26.8) | RR 0.80 (0.64, 0.99) | RR 0.86 (0.69, 1.06) | 0.159 |  |
| Hypertensive disorders of pregnancy | 17/46 (37.0) | 12/49 (24.5) | RR 1.51 (0.81, 2.83) | RR 1.28 (0.71, 2.31) | 0.415 |  |
| Rhesus iso-immunization | 9/25 (36.0) | 5/21 (23.8) | RR 1.51 (0.58, 3.92) | RR 0.89 (0.36, 2.17) | 0.790 |  |
| Placenta previa | 3/11 (27.3) | 3/14 (21.4) | RR 1.27 (0.29, 5.53) | RR 1.07 (0.28, 4.06) | 0.926 |  |
| Diabetes mellitus | 0/5 (0.0) | 5/11 (45.5) | NE | NE | NE |  |
| **Chronic respiratory illness (Self-reported diagnosis of asthma, COPD IPAG questionnaire >19.5, admissions for asthma or COPD or prescription of pharmaceuticals for asthma or COPD), n/N (%)** | | | | | | |
| **Trial protocol** |  |  |  |  |  | 0.920 |
| Standard-dose treatment | 44/145 (30.3) | 38/123 (30.9) | RR 0.98 (0.68, 1.41) | RR 1.02 (0.71, 1.47) | 0.912 |  |
| Double-standard dose treatment | 33/84 (39.3) | 28/72 (38.9) | RR 1.01 (0.68, 1.50) | RR 0.99 (0.67, 1.48) | 0.972 |  |
| **Multiple Pregnancy** |  |  |  |  |  | 0.160 |
| Singleton | 68/195 (34.9) | 57/175 (32.6) | RR 1.07 (0.80, 1.43) | RR 1.08 (0.81, 1.45) | 0.582 |  |
| Multiple | 9/34 (26.5) | 9/20 (45.0) | RR 0.59 (0.28, 1.26) | RR 0.61 (0.29, 1.29) | 0.198 |  |
| **Infant sex** |  |  |  |  |  | 0.984 |
| Female | 40/105 (38.1) | 40/107 (37.4) | RR 1.02 (0.72, 1.44) | RR 1.01 (0.71, 1.44) | 0.934 |  |
| Male | 37/124 (29.8) | 26/88 (29.6) | RR 1.01 (0.66, 1.54) | RR 1.01 (0.66, 1.54) | 0.966 |  |
| **Tocolytic used** |  |  |  |  |  | 0.568 |
| Ethanol | 12/38 (31.6) | 8/29 (27.6) | RR 1.14 (0.53, 2.47) | RR 1.22 (0.57, 2.60) | 0.609 |  |
| Salbutamol | 39/111 (35.1) | 37/96 (38.5) | RR 0.91 (0.64, 1.31) | RR 0.92 (0.64, 1.32) | 0.654 |  |
| Both | 2/7 (28.6) | 3/5 (60.0) | RR 0.48 (0.10, 2.27) | RR 0.48 (0.12, 1.91) | 0.295 |  |
| None | 24/73 (32.9) | 18/65 (27.7) | RR 1.19 (0.71, 1.99) | RR 1.19 (0.71, 1.98) | 0.515 |  |
| **Reason for preterm birth** |  |  |  |  |  | 0.950 |
| Unplanned preterm birth | 65/193 (33.7) | 55/161 (34.2) | RR 0.99 (0.74, 1.32) | RR 1.01 (0.75, 1.36) | 0.934 |  |
| Hypertensive disorders of pregnancy | 7/20 (35.0) | 6/19 (31.6) | RR 1.11 (0.44, 2.79) | RR 1.06 (0.43, 2.59) | 0.898 |  |
| Rhesus iso-immunization | 3/10 (30.0) | 2/8 (25.0) | RR 1.20 (0.23, 6.27) | RR 1.33 (0.29, 6.13) | 0.718 |  |
| Placenta previa | 2/4 (50.0) | 1/4 (25.0) | RR 2.00 (0.17, 23.11) | RR 2.22 (0.31, 15.83) | 0.425 |  |
| Diabetes mellitus | 0/2 (0.0) | 2/3 (66.7) | NE | NE | NE |  |
| **Asthma, n/N (%)** | | | | | | |
| **Trial protocol** |  |  |  |  |  | 0.757 |
| Standard-dose treatment | 44/145 (30.3) | 37/123 (30.1) | RR 1.01 (0.70, 1.46) | RR 1.04 (0.72, 1.51) | 0.831 |  |
| Double-standard dose treatment | 32/84 (38.1) | 28/72 (38.9) | RR 0.98 (0.66, 1.46) | RR 0.96 (0.64, 1.43) | 0.823 |  |
| **Multiple Pregnancy** |  |  |  |  |  | 0.153 |
| Singleton | 67/195 (34.4) | 56/175 (32.0) | RR 1.07 (0.80, 1.44) | RR 1.08 (0.81, 1.45) | 0.607 |  |
| Multiple | 9/34 (26.5) | 9/20 (45.0) | RR 0.59 (0.28, 1.26) | RR 0.61 (0.29, 1.27) | 0.186 |  |
| **Infant sex** |  |  |  |  |  | 0.998 |
| Female | 39/105 (37.1) | 39/107 (36.5) | RR 1.02 (0.71, 1.45) | RR 1.01 (0.70, 1.44) | 0.969 |  |
| Male | 37/124 (29.8) | 2688 (29.6) | RR 1.01 (0.66, 1.54) | RR 1.01 (0.66, 1.54) | 0.971 |  |
| **Tocolytic used** |  |  |  |  |  | 0.473 |
| Ethanol | 12/38 (31.6) | 8/29 (27.6) | RR 1.14 (0.53, 2.47) | RR 1.20 (0.57, 2.57) | 0.629 |  |
| Salbutamol | 38/111 (34.2) | 37/96 (38.5) | RR 0.89 (0.62, 1.28) | RR 0.90 (0.62, 1.28) | 0.547 |  |
| Both | 2/7 (28.6) | 3/5 (60.0) | RR 0.48 (0.10, 2.27) | RR 0.46 (0.12, 1.85) | 0.275 |  |
| None | 24/73 (32.9) | 17/65 (26.2) | RR 1.26 (0.74, 2.13) | RR 1.25 (0.74, 2.12) | 0.410 |  |
| **Reason for preterm birth** |  |  |  |  |  | 0.997 |
| Unplanned preterm birth | 65/193 (33.7) | 55/161 (34.2) | RR 0.99 (0.74, 1.32) | RR 1.01 (0.75, 1.35) | 0.962 |  |
| Hypertensive disorders of pregnancy | 6/20 (30.0) | 6/19 (31.6) | RR 0.95 (0.36, 2.51) | RR 0.90 (0.35, 2.32) | 0.832 |  |
| Rhesus iso-immunization | 3/10 (30.0) | 2/8 (25.0) | RR 1.20 (0.23, 6.27) | RR 1.31 (0.28, 6.07) | 0.729 |  |
| Placenta previa | 2/4 (50.0) | 0/4 (0.0) | NE | NE | NE |  |
| Diabetes mellitus | 0/2 (0.0) | 2/3 (66.7) | NE | NE | NE |  |
| **Chronic obstructive pulmonary disease diagnosis, n/N (%)** | | | | | | |
| **Trial protocol** |  |  |  |  |  | 0.972 |
| Standard-dose treatment | 2/145 (1.4) | 4/123 (3.3) | RR 0.42 (0.08, 2.29) | RR 0.55 (0.10, 2.99) | 0.488 |  |
| Double-standard dose treatment | 2/84 (2.4) | 0/72 (0.0) | NE | NE | NE |  |
| **Multiple Pregnancy** |  |  |  |  |  | 1.000 |
| Singleton | 4/195 (2.1) | 4/175 (2.3) | RR 0.90 (0.23, 3.55) | RR 1.18 (0.29, 4.77) | 0.812 |  |
| Multiple | 0/34 (0.0) | 0/20 (0.0) | NE | NE | NE |  |
| **Infant sex** |  |  |  |  |  | 0.971 |
| Female | 2/105 (1.9) | 4/107 (3.7) | RR 0.51 (0.09, 2.75) | RR 0.62 (0.11, 3.32) | 0.572 |  |
| Male | 2/124 (1.6) | 0/88 (0.0) | NE | NE | NE |  |
| **Tocolytic used** |  |  |  |  |  | 0.997 |
| Ethanol | 0/38 (0.0) | 0/29 (0.0) | NE | NE | NE |  |
| Salbutamol | 3/111 (2.7) | 2/96 (2.1) | RR 1.30 (0.22, 7.68) | RR 1.59 (0.27 9.35) | 0.609 |  |
| Both | 0/7 (0.0) | 1/5 (20.0) | NE | NE | NE |  |
| None | 1/73 (1.4) | 1/65 (1.5) | RR 0.89 (0.06, 14.30) | RR 1.07 (0.07, 16.82) | 0.960 |  |
| **Reason for preterm birth** |  |  |  |  |  | 1.000 |
| Unplanned preterm birth | 2/193 (1.0) | 3/161 (1.9) | RR 0.56 (0.09, 3.31) | RR 0.71 (0.12, 4.24) | 0.705 |  |
| Hypertensive disorders of pregnancy | 2/20 (10.0) | 0/19 (0.0) | NE | NE | NE |  |
| Rhesus iso-immunization | 0/10 (0.0) | 0/8 (0.0) | NE | NE | NE |  |
| Placenta previa | 0/4 (0.0) | 1/4 (25.0) | NE | NE | NE |  |
| Diabetes mellitus | 0/2 (0.0) | 0/3 (0.0) | NE | NE | NE |  |
| **IPAG COPD questionnaire score >19.5, (n/N, %)** | | | | | | |
| **Trial protocol** |  |  |  |  |  | 0.975 |
| Standard-dose treatment | 1/113 (0.9) | 2/90 (2.2) | RR 0.40 (0.04, 4.38) | RR 0.39 (0.04, 4.43) | 0.450 |  |
| Double-standard dose treatment | 2/66 (3.0) | 0/57 (0.0) | NE | NE | NE |  |
| **Multiple Pregnancy** |  |  |  |  |  | 0.974 |
| Singleton | 2/152 (1.3) | 2/133 (1.5) | RR 0.88 (0.12, 6.18) | RR 0.91 (0.13, 6.45) | 0.921 |  |
| Multiple | 1/27 (3.7) | 0/14 (0.0) | NE | NE | NE |  |
| **Infant sex** |  |  |  |  |  | 0.912 |
| Female | 1/76 (1.3) | 1/81 (1.2) | RR 1.07 (0.07, 17.11) | RR 1.08 (0.07, 17.12) | 0.956 |  |
| Male | 2/103 (1.9) | 1/66 (1.5) | RR 1.28 (0.12, 14.10) | RR 1.33 (0.12, 14.46) | 0.816 |  |
| **Tocolytic used** |  |  |  |  |  | 1.000 |
| Ethanol | 0/32 (0.0) | 1/21 (4.8) | NE | NE | NE |  |
| Salbutamol | 3/87 (3.5) | 0/74 (0.0) | NE | NE | NE |  |
| Both | 0/3 (0.0) | 0/3 (0.0) | NE | NE | NE |  |
| None | 0/57 (0.0) | 1/49(2.0) | NE | NE | NE |  |
| **Reason for preterm birth** |  |  |  |  |  | 1.000 |
| Unplanned preterm birth | 3/150 (2.0) | 1/121 (0.8) | RR 2.42 (0.25, 23.21) | RR 2.30 (0.24, 22.38) | 0.473 |  |
| Hypertensive disorders of pregnancy | 0/16 (0.0) | 1/15 (6.7) | NE | NE | NE |  |
| Rhesus iso-immunization | 0/10 (0.0) | 0/7 (0.0) | NE | NE | NE |  |
| Placenta previa | 0/3 (0.0) | 0/3 (0.0) | NE | NE | NE |  |
| Diabetes mellitus | - | 0/1 (0.0) | NE | NE | NE |  |
| **Proportion with at least one admission for asthma or chronic obstructive pulmonary disease, n/N (%)** | | | | | | |
| **Trial protocol** |  |  |  |  |  | 0.523 |
| Standard-dose treatment | 13/142 (9.2) | 9/118 (7.6) | RR 1.20 (0.53, 2.72) | RR 1.37 (0.61, 3.08) | 0.446 |  |
| Double-standard dose treatment | 7/81 (8.6) | 7/70 (10.0) | RR 0.86 (0.32, 2.36) | RR 0.90 (0.33, 2.46) | 0.838 |  |
| **Multiple Pregnancy** |  |  |  |  |  | 0.980 |
| Singleton | 19/190 (10.0) | 16/169 (9.5) | RR 1.06 (0.56, 1.99) | RR 1.16 (0.62, 2.18) | 0.638 |  |
| Multiple | 1/33 (3.0) | 0/19 (0.0) | NE | NE | NE |  |
| **Infant sex** |  |  |  |  |  | 0.162 |
| Female | 13/101 (12.9) | 8/104 (7.7) | RR 1.67 (0.72, 3.88) | RR 1.71 (0.74, 3.97) | 0.211 |  |
| Male | 7/122 (5.7) | 8/84 (9.5) | RR 0.60 (0.23, 1.61) | RR 0.70 (0.27, 1.78) | 0.449 |  |
| **Tocolytic used** |  |  |  |  |  | 0.563 |
| Ethanol | 4/35 (11.4) | 2/28 (7.1) | RR 1.6 (0.31, 8.38) | RR 1.72 (0.34, 8.79) | 0.514 |  |
| Salbutamol | 7/109 (6.4) | 8/92 (8.7) | RR 0.74 (0.28, 1.97) | RR 0.88 (0.34, 2.25) | 0.782 |  |
| Both | 1/7 (14.3) | 2/5 (40.0) | RR 0.36 (0.03, 3.92) | RR 0.43 (0.05, 3.65) | 0.439 |  |
| None | 8/72 (11.1) | 4/63 (6.4) | RR 1.75 (0.55, 5.59) | RR 1.85 (0.58, 5.89) | 0.297 |  |
| **Reason for preterm birth** |  |  |  |  |  | 0.862 |
| Unplanned preterm birth | 16/188 (8.5) | 14/154 (9.1) | RR 0.94 (0.47, 1.86) | RR 1.05 (0.53, 2.07) | 0.897 |  |
| Hypertensive disorders of pregnancy | 4/20 (20.0) | 1/19 (5.3) | RR 3.80 (0.43, 33.29) | RR 3.78 (0.46, 31.04) | 0.215 |  |
| Rhesus iso-immunization | 0/10 (0.0) | 0/8 (0.0) | NE | NE | NE |  |
| Placenta previa | 0/3 (0.0) | 0/4 (0.0) | NE | NE | NE |  |
| Diabetes mellitus | 0/2 (0.0) | 1/3 (33.3) | NE | NE | NE |  |
| **Prescriptions of pharmaceuticals for asthma or COPD, n/N (%)** | | | | | | |
| **Trial protocol** |  |  |  |  |  | 0.269 |
| Standard-dose treatment | 35/140 (25.0) | 28/114 (24.6) | RR 1.02 (0.66, 1.57) | RR 1.08 (0.70, 1.67) | 0.719 |  |
| Double-standard dose treatment | 34/79 (43.0) | 19/68 (27.9) | RR 1.54 (0.97, 2.45) | RR 1.55 (0.97, 2.45) | 0.064 |  |
| **Multiple Pregnancy** |  |  |  |  |  | 0.162 |
| Singleton | 60/187 (32.1) | 40/165 (24.2) | RR 1.32 (0.94, 1.86) | RR 1.37 (0.97, 1.93) | 0.075 |  |
| Multiple | 9/32 (28.1) | 7/17 (41.2) | RR 0.68 (0.30, 1.54) | RR 0.74 (0.33, 1.64) | 0.458 |  |
| **Infant sex** |  |  |  |  |  | 0.366 |
| Female | 38/98 (38.8) | 27/99 (27.3) | RR 1.42 (0.94, 2.14) | RR 1.43 (0.95, 2.16) | 0.089 |  |
| Male | 31/121 (25.6) | 20/83 (24.1) | RR 1.06 (0.65, 1.74) | RR 1.06 (0.65, 1.74) | 0.801 |  |
| **Tocolytic used** |  |  |  |  |  | 0.386 |
| Ethanol | 10/34 (29.4) | 5/25 (20.0) | RR 1.47 (0.56, 3.85) | RR 1.62 (0.63, 4.18) | 0.315 |  |
| Salbutamol | 34/106 (32.1) | 24/90 (26.7) | RR 1.20 (0.77, 1.87) | RR 1.23 (0.79, 1.92) | 0.353 |  |
| Both | 1/7 (14.3) | 3/5 (60.0) | RR 0.24 (0.03, 2.19) | RR 0.25 (0.04, 1.79) | 0.168 |  |
| None | 24/72 (33.3) | 15/62 (24.2) | RR 1.38 (0.79, 2.40) | RR 1.40 (0.81, 2.42) | 0.232 |  |
| **Reason for preterm birth** |  |  |  |  |  | 0.931 |
| Unplanned preterm birth | 57/184 (31.0) | 37/149 (24.8) | RR 1.25 (0.88, 1.78) | RR 1.31 (0.92, 1.87) | 0.138 |  |
| Hypertensive disorders of pregnancy | 7/20 (35.0) | 5/18 (27.8) | RR 1.26 (0.47, 3.38) | RR 1.19 (0.46, 3.08) | 0.72 |  |
| Rhesus iso-immunization | 3/10 (30.0) | 2/8 (25.0) | RR 1.20 (0.23, 6.27) | RR 1.45 (0.31, 6.75) | 0.632 |  |
| Placenta previa | 2/3 (66.7) | 1/4 (25.0) | RR 2.67 (0.23, 31.25) | RR 3.09 (0.47, 20.22) | 0.238 |  |
| Diabetes mellitus | 0/2 (0.0) | 2/3 (66.7) | NE | NE | NE |  |

CI: Confidence interval. COPD: Chronic obstructive pulmonary disease. IPAG: International primary care airways group. HR: Hazard ratio. MACE: Major adverse cardiovascular event. MD: Mean difference. NE: Not estimable. RR: Relative risk. SD: Standard deviation.

*Analyses adjusted for sex and gestational age at trial entry.

Table D: Sensitivity analysis for win ratio hierarchical outcome.

| **Outcome** | **Betamethasone**  **Wins** | **Placebo**  **Wins** | **Win Ratio (95% CI)** | **P value** |
| --- | --- | --- | --- | --- |
| **Win ratio Hierarchical outcome excluding stillbirth** | 80,288 | 67,115 | 1.20 (0.97, 1.47) | 0.092 |
| **Win ratio Hierarchical outcome excluding death before 28 days of age** | 41,168 | 35,691 | 1.15 (0.90, 1.48) | 0.261 |

CI: Confidence interval.

Table E: Sensitivity analyses for primary and secondary outcomes.

| **Primary or secondary outcome** | **Betamethasone** | **Placebo** | **Unadjusted RR, HR or mean difference (95% CI)** | **Adjusted* RR, HR or mean difference**  **(95% CI)** | **P value (Adjusted*)** |
| --- | --- | --- | --- | --- | --- |
| **Cardiometabolic risk factor composite, n/N (%)** | | | | | |
| Using per protocol analysis | 155/223 (69.5) | 127/191 (66.5) | RR 1.05 (0.91, 1.19) | RR 1.04 (0.89, 1.20) | 0.619 |
| Including additional covariates in the statistical model^1^ | | | | | |
| BMI | 159/229 (69.4) | 131/195 (67.2) | RR 1.03 (0.91, 1.18) | RR 0.97 (0.84, 1.12) | 0.673 |
| Including GA at birth rather than GA at entry | 159/229 (69.4) | 131/195 (67.2) | RR 1.03 (0.91, 1.18) | RR 1.01 (0.89, 1.16) | 0.849 |
| Birthweight Z score | 159/229 (69.4) | 131/195 (67.2) | RR 1.03 (0.91, 1.18) | RR 1.04 (0.91, 1.19) | 0.568 |
| SES | 159/229 (69.4) | 131/195 (67.2) | RR 1.03 (0.91, 1.18) | RR 1.02 (0.89, 1.16) | 0.81 |
| Excluding the second child in cases of women having more than one pregnancy within the study. | 154/223 (69.1) | 129/191 (67.5) | RR 1.02 (0.90, 1.17) | RR 1.01 (0.87, 1.16) | 0.919 |
| **Age at first major adverse cardiovascular event, years, median (5^th^, 95^th^ centiles)** | | | | | |
| Using per protocol analysis | 41.8 (20.5, 48.5) | 43.8 (24.2, 47.6) | HR 0.64 (0.25, 1.66) | HR 0.67 (0.26, 1.72) | 0.401 |
| Including additional covariates in the statistical model^1^ | | | | | |
| BMI | 41.8 (20.5, 48.5) | 44.0 (24.2, 50.3) | HR 0.58 (0.23, 1.47) | HR 0.44 (0.12, 1.58) | 0.209 |
| Including GA at birth rather than GA at entry | 41.8 (20.5, 48.5) | 44.0 (24.2, 50.3) | HR 0.58 (0.23, 1.47) | HR 0.59 (0.23, 1.51) | 0.272 |
| Birthweight Z score | 41.8 (20.5, 48.5) | 44.0 (24.2, 50.3) | HR 0.58 (0.23, 1.47) | HR 0.59 (0.23, 1.49) | 0.262 |
| SES | 41.8 (20.5, 48.5) | 44.0 (24.2, 50.3) | HR 0.58 (0.23, 1.47) | HR 0.84 (0.24, 2.95) | 0.788 |
| Excluding the second child in cases of women having more than one pregnancy within the study. | 41.8 (20.5, 48.5) | 44.0 (24.2, 50.3) | HR 0.58 (0.23, 1.48) | HR 0.59 (0.23, 1.50) | 0.267 |
| **Diabetes mellitus, prediabetes or gestational diabetes mellitus, n/N (%)** | | | | | |
| Using per protocol analysis | 47/223 (21.1) | 39/191 (20.4) | RR 1.03 (0.71, 1.51) | RR 1.05 (0.70, 1.57) | 0.821 |
| Including additional covariates in the statistical model^1^ | | | | | |
| BMI | 48/229 (21.0) | 41/195 (21.0) | RR 1.00 (0.69, 1.45) | RR 0.83 (0.53, 1.32) | 0.436 |
| Including GA at birth rather than GA at entry | 48/229 (21.0) | 41/195 (21.0) | RR 1.00 (0.69, 1.45) | RR 0.96 (0.66, 1.39) | 0.815 |
| Birthweight Z score | 48/229 (21.0) | 41/195 (21.0) | RR 1.00 (0.69, 1.45) | RR 1.00 (0.70, 1.46) | 0.981 |
| SES | 48/229 (21.0) | 41/195 (21.0) | RR 1.00 (0.69, 1.45) | RR 0.93 (0.64, 1.36) | 0.716 |
| Excluding the second child in cases of women having more than one pregnancy within the study. | 47/223 (21.1) | 41/191 (21.5) | RR 0.98 (0.68, 1.43) | RR 0.98 (0.66, 1.46) | 0.918 |
| **Diabetes mellitus, n/N (%)** | | | | | |
| Using per protocol analysis | 15/223 (6.7) | 19/191 (10.0) | RR 0.68 (0.35, 1.30) | RR 0.68 (0.33, 1.37) | 0.255 |
| Including additional covariates in the statistical model^1^ | | | | | |
| BMI | 15/229 (6.6) | 21/195 (10.8) | RR 0.61 (0.32, 1.15) | RR 0.44 (0.21, 0.90) | 0.025 |
| Including GA at birth rather than GA at entry | 15/229 (6.6) | 21/195 (10.8) | RR 0.61 (0.32, 1.15) | RR 0.58 (0.31, 1.11) | 0.098 |
| Birthweight Z score | 15/229 (6.6) | 21/195 (10.8) | RR 0.61 (0.32, 1.15) | RR 0.60 (0.32, 1.14) | 0.116 |
| SES | 15/229 (6.6) | 21/195 (10.8) | RR 0.61 (0.32, 1.15) | RR 0.60 (0.32, 1.13) | 0.115 |
| Excluding the second child in cases of women having more than one pregnancy within the study. | 15/223 (6.7) | 21/191 (11.0) | RR 0.61 (0.32, 1.16) | RR 0.59 (0.30, 1.19) | 0.129 |
| **Prediabetes, n/N (%)** | | | | | |
| Using per protocol analysis | 29/222 (13.1) | 18/190 (9.5) | RR 1.38 (0.79, 2.41) | RR 1.38 (0.75, 2.52) | 0.277 |
| Including additional covariates in the statistical model^1^ | | | | | |
| BMI | 30/228 (13.2) | 18/194 (9.3) | RR 1.41 (0.82, 2.47) | RR 1.65 (0.85, 3.23) | 0.140 |
| Including GA at birth rather than GA at entry | 30/228 (13.2) | 18/194 (9.3) | RR 1.41 (0.82, 2.47) | RR 1.34 (0.77, 2.34) | 0.298 |
| Birthweight Z score | 30/228 (13.2) | 18/194 (9.3) | RR 1.41 (0.82, 2.47) | RR 1.42 (0.818, 2.47) | 0.222 |
| SES | 30/228 (13.2) | 18/194 (9.3) | RR 1.41 (0.82, 2.47) | RR 1.26 (0.71, 2.23) | 0.422 |
| Excluding the second child in cases of women having more than one pregnancy within the study. | 29/222 (13.1) | 18/190 (9.5) | RR 1.38 (0.79, 2.41) | RR 1.39 (0.76, 2.54) | 0.261 |
| **Gestational diabetes mellitus, n/N (%)** | | | | | |
| Using per protocol analysis | 7/103 (6.8) | 3/104 (2.9) | RR 2.36 (0.62, 8.93) | RR 2.67 (0.47, 15.00) | 0.204 |
| Including additional covariates in the statistical model^1^ | | | | | |
| BMI | 7/105 (6.7) | 3/106 (2.8) | RR 2.36 (0.62, 8.93) | RR 2.06 (0.53, 8.00) | 0.294 |
| Including GA at birth rather than GA at entry | 7/105 (6.7) | 3/106 (2.8) | RR 2.36 (0.62, 8.93) | RR 2.28 (0.60, 8.67) | 0.226 |
| Birthweight Z score | 7/105 (6.7) | 3/106 (2.8) | RR 2.36 (0.62, 8.93) | RR 2.53 (0.66, 9.61) | 0.173 |
| SES | 7/105 (6.7) | 3/106 (2.8) | RR 2.36 (0.62, 8.93) | RR 2.67 (0.70, 10.16) | 0.148 |
| Excluding the second child in cases of women having more than one pregnancy within the study. | 7/101 (6.9) | 3/104 (2.9) | RR 2.40 (0.63, 9.11) | RR 2.70 (0.42, 17.41) | 0.212 |
| **Hypertension, n/N (%)** | | | | | |
| Using per protocol analysis | 70/222 (31.5) | 62/191 (32.5) | RR 0.97 (0.73, 1.29) | RR 0.97 (0.72, 1.31) | 0.821 |
| Including additional covariates in the statistical model^1^ | | | | | |
| BMI | 72/228 (31.6) | 64/195 (32.8) | RR 0.96 (0.73, 1.27) | RR 0.95 (0.72, 1.25) | 0.723 |
| Including GA at birth rather than GA at entry | 72/228 (31.6) | 64/195 (32.8) | RR 0.96 (0.73, 1.27) | RR 0.95 (0.72, 1.25) | 0.699 |
| Birthweight Z score | 72/228 (31.6) | 64/195 (32.8) | RR 0.96 (0.73, 1.27) | RR 0.96 (0.73, 1.27) | 0.771 |
| SES | 72/228 (31.6) | 64/195 (32.8) | RR 0.96 (0.73, 1.27) | RR 0.97 (0.72, 1.30) | 0.832 |
| Excluding the second child in cases of women having more than one pregnancy within the study. | 70/222 (31.5) | 62/191 (32.5) | RR 0.97 (0.73, 1.29) | RR 0.96 (0.71, 1.30) | 0.785 |
| **Hyperlipidemia, n/N (%)** | | | | | |
| Using per protocol analysis | 129/222 (58.1) | 109/190 (57.4) | RR 1.01 (0.86, 1.20) | RR 1.00 (0.83, 1.19) | 0.975 |
| Including additional covariates in the statistical model^1^ | | | | | |
| BMI | 131/228 (57.5) | 112/194 (57.7) | RR 1.00 (0.84, 1.17) | RR 0.93 (0.77, 1.12) | 0.438 |
| Including GA at birth rather than GA at entry | 131/228 (57.5) | 112/194 (57.7) | RR 1.00 (0.84, 1.17) | RR 0.97 (0.83, 1.14) | 0.723 |
| Birthweight Z score | 131/228 (57.5) | 112/194 (57.7) | RR 1.00 (0.84, 1.17) | RR 1.00 (0.84, 1.17) | 0.957 |
| SES | 131/228 (57.5) | 112/194 (57.7) | RR 1.00 (0.84, 1.17) | RR 0.95 (0.81, 1.12) | 0.534 |
| Excluding the second child in cases of women having more than one pregnancy within the study. | 128/222 (57.7) | 112/190 (59.0) | 0.98 (0.83, 1.15) | 0.96 (0.80, 1.14) | 0.610 |
| **At least one admission for major adverse cardiovascular event, n/N (%)** | | | | | |
| Using per protocol analysis | 7/482 (1.5) | 11/489 (2.3) | RR 0.65 (0.25, 1.65) | RR 0.66 (0.26, 1.68) | 0.374 |
| Including additional covariates in the statistical model^1^ | | | | | |
| BMI | 7/493 (1.4) | 12/494 (2.4) | RR 0.58 (0.23, 1.47) | RR 0.50 (0.15, 1.65) | 0.253 |
| Including GA at birth rather than GA at entry | 7/493 (1.4) | 12/494 (2.4) | RR 0.58 (0.23, 1.47) | RR 0.61 (0.24, 1.53) | 0.287 |
| Birthweight Z score | 7/493 (1.4) | 12/494 (2.4) | RR 0.58 (0.23, 1.47) | RR 0.60 (0.24, 1.51) | 0.274 |
| SES | 7/493 (1.4) | 12/494 (2.4) | RR 0.58 (0.23, 1.47) | RR 0.87 (0.25, 2.96) | 0.817 |
| Excluding the second child in cases of women having more than one pregnancy within the study. | 7/481 (1.5) | 12/483 (2.5) | RR 0.59 (0.23, 1.48) | RR 0.59 (0.23, 1.49) | 0.259 |
| **Age at cardiovascular death, years, median (5^th^, 95^th^ centiles)** | | | | | |
| Using per protocol analysis | 33.1 (20.5, 45.8) | 46.9 (46.0, 47.7) | HR 1.01 (0.14, 7.16) | HR 0.97 (0.14, 6.93) | 0.977 |
| Including additional covariates in the statistical model^1^ | | | | | |
| BMI | Not performed as BMI data not available for deceased participants. | | | | |
| Including GA at birth rather than GA at entry | 33.1 (20.5, 45.8) | 46.9 (46.0, 47.7) | HR 1.00 (0.14, 7.07) | HR 1.04 (0.15, 7.42) | 0.966 |
| Birthweight Z score | 33.1 (20.5, 45.8) | 46.9 (46.0, 47.7) | HR 1.00 (0.14, 7.07) | HR 0.96 (0.13, 6.85) | 0.967 |
| SES | 33.1 (20.5, 45.8) | 46.9 (46.0, 47.7) | HR 1.00 (0.14, 7.07) | NE | NE |
| Excluding the second child in cases of women having more than one pregnancy within the study. | 33.1 (20.5, 45.8) | 46.9 (46.0, 47.7) | HR 1.00 (0.14, 7.11) | HR 0.96 (0.13, 6.86) | 0.965 |
| **Cardiovascular death, n/N (%)** | | | | | |
| Using per protocol analysis | 2/482 (0.4) | 2/489 (0.4) | RR 1.01 (0.14, 7.19) | RR 0.99 (0.13, 7.21) | 0.988 |
| Including additional covariates in the statistical model^1^ | | | | | |
| BMI | Not performed as BMI data not available for deceased participants. | | | | |
| Including GA at birth rather than GA at entry | 2/493 (0.4) | 2/494 (0.4) | RR 1.00 (0.14, 7.10) | RR 1.06 (0.15, 7.52) | 0.951 |
| Birthweight Z score | 2/493 (0.4) | 2/494 (0.4) | RR 1.00 (0.14, 7.10) | RR 0.97 (0.14, 6.93) | 0.979 |
| SES | 2/493 (0.4) | 2/494 (0.4) | RR 1.00 (0.14, 7.10) | NE | NE |
| Excluding the second child in cases of women having more than one pregnancy within the study. | 2/481 (0.4) | 2/483 (0.4) | RR 1.00 (0.14, 7.12) | RR 0.97 (0.13, 7.17) | 0.973 |
| **Age at first admission for myocardial infarction or coronary revascularization, years, median (5^th^, 95^th^ centiles)** | | | | | |
| Using per protocol analysis | 44.6 (41.4, 45.2) | 40.9 (26.4, 47.1) | HR 1.27 (0.34, 4.72) | HR 1.30 (0.35, 4.8) | 0.699 |
| Including additional covariates in the statistical model^1^ | | | | | |
| BMI | 44.6 (41.4, 45.2) | 40.9 (26.4, 47.1) | HR 1.25 (0.34, 4.66) | HR 2.01 (0.20, 19.90) | 0.553 |
| Including GA at birth rather than GA at entry | 44.6 (41.4, 45.2) | 40.9 (26.4, 47.1) | HR 1.25 (0.34, 4.66) | HR 1.24 (0.33, 4.66) | 0.746 |
| Birthweight Z score | 44.6 (41.4, 45.2) | 40.9 (26.4, 47.1) | HR 1.25 (0.34, 4.66) | HR 1.29 (0.35, 4.84) | 0.702 |
| SES | 44.6 (41.4, 45.2) | 40.9 (26.4, 47.1) | HR 1.25 (0.34, 4.66) | HR 3.40 (0.38, 30.84) | 0.278 |
| Excluding the second child in cases of women having more than one pregnancy within the study. | 44.6 (41.4, 45.2) | 40.9 (26.4, 47.1) | HR 1.25 (0.34, 4.67) | HR 1.30 (0.35, 4.86) | 0.700 |
| **At least one admission for myocardial infarction or coronary revascularization, n/N (%)** | | | | | |
| Using per protocol analysis | 5/482 (1.0) | 4/489 (0.8) | RR 1.27 (0.34, 4.70) | RR 1.30 (0.34, 4.96) | 0.698 |
| Including additional covariates in the statistical model^1^ | | | | | |
| BMI | 5/493 (1.0) | 4/494 (0.8) | RR 1.25 (0.34, 4.64) | RR 2.05 (0.22, 19.58) | 0.530 |
| Including GA at birth rather than GA at entry | 5/493 (1.0) | 4/494 (0.8) | RR 1.25 (0.34, 4.64) | RR 1.25 (0.33, 4.64) | 0.743 |
| Birthweight Z score | 5/493 (1.0) | 4/494 (0.8) | RR 1.25 (0.34, 4.64) | RR 1.29 (0.35, 4.82) | 0.701 |
| SES | 5/493 (1.0) | 4/494 (0.8) | RR 1.25 (0.34, 4.64) | RR 3.40 (.038, 30.45) | 0.273 |
| Excluding the second child in cases of women having more than one pregnancy within the study. | 5/481 (1.0) | 4/483 (0.8) | RR 1.26 (0.34, 4.65) | RR 1.30 (0.34, 5.00) | 0.699 |
| **Age at first admission for myocardial infarction, years, median (5^th^, 95^th^ centiles)** | | | | | |
| Using per protocol analysis | 43.7 (41.4, 45.2) | 41.9 (37.6, 46.2) | HR 2.03 (0.37, 11.10) | HR 2.10 (0.38, 11.53) | 0.393 |
| Including additional covariates in the statistical model^1^ | | | | | |
| BMI | 43.7 (41.4, 45.2) | 41.9 (37.6, 46.2) | HR 2.01 (0.37, 10.96) | HR 1.46 (0.13, 16.78) | 0.760 |
| Including GA at birth rather than GA at entry | 43.7 (41.4, 45.2) | 41.9 (37.6, 46.2) | HR 2.01 (0.37, 10.96) | HR 1.99 (0.36, 10.91) | 0.429 |
| Birthweight Z score | 43.7 (41.4, 45.2) | 41.9 (37.6, 46.2) | HR 2.01 (0.37, 10.96) | HR 2.09 (0.38, 11.46) | 0.397 |
| SES | 43.7 (41.4, 45.2) | 41.9 (37.6, 46.2) | HR 2.01 (0.37, 10.96) | HR 2.67 (0.27, 26.10) | 0.399 |
| Excluding the second child in cases of women having more than one pregnancy within the study. | 43.7 (41.4, 45.2) | 41.9 (37.6, 46.2) | HR 2.01 (0.37, 10.98) | HR 2.11 (0.38, 11.60) | 0.391 |
| **At least one admission for myocardial infarction, n/N (%)** | | | | | |
| Using per protocol analysis | 4/482 (0.8) | 2/489 (0.4) | RR 2.03 (0.37, 11.05) | RR 2.10 (0.37, 11.87) | 0.395 |
| Including additional covariates in the statistical model^1^ | | | | | |
| BMI | 4/493 (0.8) | 2/494 (0.4) | RR 2.00 (0.37, 10.91) | RR 1.51 (0.14, 16.65) | 0.737 |
| Including GA at birth rather than GA at entry | 4/493 (0.8) | 2/494 (0.4) | RR 2.00 (0.37, 10.91) | RR 1.99 (0.36, 10.87) | 0.429 |
| Birthweight Z score | 4/493 (0.8) | 2/494 (0.4) | RR 2.00 (0.37, 10.91) | RR 2.08 (0.38, 11.38) | 0.398 |
| SES | 4/493 (0.8) | 2/494 (0.4) | RR 2.00 (0.37, 10.91) | RR 2.68 (0.28, 25.96) | 0.394 |
| Excluding the second child in cases of women having more than one pregnancy within the study. | 4/481 (0.8) | 2/483 (0.4) | RR 2.01 (0.37, 10.94) | RR 2.11 (0.37, 12.02) | 0.395 |
| **Age at first admission for coronary revascularization, years, median (5^th^, 95^th^ centiles)** | | | | | |
| Using per protocol analysis | 44.6 (44.6, 44.6) | 40.9 (26.4, 47.1) | HR 0.25 (0.03, 2.26) | HR 0.28 (0.03, 2.50) | 0.253 |
| Including additional covariates in the statistical model^1^ | | | | | |
| BMI | 44.6 (44.6, 44.6) | 40.9 (26.4, 47.1) | HR 0.25 (0.03, 2.23) | HR 0.78 (0.05, 12.86) | 0.737 |
| Including GA at birth rather than GA at entry | 44.6 (44.6, 44.6) | 40.9 (26.4, 47.1) | HR 0.25 (0.03, 2.23) | HR 0.25 (0.03, 2.27) | 0.429 |
| Birthweight Z score | 44.6 (44.6, 44.6) | 40.9 (26.4, 47.1) | HR 0.25 (0.03, 2.23) | HR 0.28 (0.03, 2.53) | 0.398 |
| SES | 44.6 (44.6, 44.6) | 40.9 (26.4, 47.1) | HR 0.25 (0.03, 2.23) | HR 0.92 (0.06, 15.09) | 0.394 |
| Excluding the second child in cases of women having more than one pregnancy within the study. | 44.6 (44.6, 44.6) | 40.9 (26.4, 47.1) | HR 0.25 (0.03, 2.24) | HR 0.28 (0.03, 2.53) | 0.395 |
| **At least one admission for coronary revascularization, n/N (%)** | | | | | |
| Using per protocol analysis | 1/482 (0.2) | 4/489 (0.8) | RR 0.25 (0.03, 2.27) | RR 0.28 (0.03, 2.62) | 0.260 |
| Including additional covariates in the statistical model^1^ | | | | | |
| BMI | 1/493 (0.2) | 4/494 (0.8) | RR 0.25 (0.03, 2.24) | RR 0.79 (0.05, 12.89) | 0.868 |
| Including GA at birth rather than GA at entry | 1/493 (0.2) | 4/494 (0.8) | RR 0.25 (0.03, 2.24) | RR 0.25 (0.03, 2.28) | 0.220 |
| Birthweight Z score | 1/493 (0.2) | 4/494 (0.8) | RR 0.25 (0.03, 2.24) | RR 0.28 (0.03, 2.54) | 0.259 |
| SES | 1/493 (0.2) | 4/494 (0.8) | RR 0.25 (0.03, 2.24) | RR 0.92 (0.06, 15.14) | 0.956 |
| Excluding the second child in cases of women having more than one pregnancy within the study. | 1/481 (0.2) | 4/483 (0.8) | RR 0.25 (0.03, 2.24) | RR 0.28 (0.03, 2.68) | 0.265 |
| **Age at first admission for peripheral vascular disease (arterial thrombosis/embolism or need for revascularization)** | | | | | |
| No sensitivity analysis performed as no events occurred in the primary analysis. | | | | | |
| **At least one admission for peripheral vascular disease (arterial thrombosis/embolism or need for revascularization)** | | | | | |
| No sensitivity analysis performed as no events occurred in the primary analysis. | | | | | |
| **Age at first admission for arterial thrombosis/embolism** | | | | | |
| No sensitivity analysis performed as no events occurred in the primary analysis. | | | | | |
| **At least one admission for arterial thrombosis/embolism** | | | | | |
| No sensitivity analysis performed as no events occurred in the primary analysis. | | | | | |
| **Age at first admission for peripheral revascularization procedure** | | | | | |
| No sensitivity analysis performed as no events occurred in the primary analysis. | | | | | |
| **At least one admission for peripheral revascularization procedure** | | | | | |
| No sensitivity analysis performed as no events occurred in the primary analysis. | | | | | |
| **Age at first admission for stroke, years, median (5^th^, 95^th^ centiles)** | | | | | |
| Using per protocol analysis | 47.0 (47.0, 47.0) | 47.6 (47.6, 47.6) | HR 1.02 (0.06, 16.22) | HR 1.63 (0.09, 29.16) | 0.739 |
| Including additional covariates in the statistical model^1^ | | | | | |
| BMI | 47.0 (47.0, 47.0) | 48.9 (47.6, 50.3) | HR 0.50 (0.05, 5.52) | HR 0.30 (0.03, 3.56) | 0.343 |
| Including GA at birth rather than GA at entry | 47.0 (47.0, 47.0) | 48.9 (47.6, 50.3) | HR 0.50 (0.05, 5.52) | HR 0.52 (0.05, 5.79) | 0.597 |
| Birthweight Z score | 47.0 (47.0, 47.0) | 48.9 (47.6, 50.3) | HR 0.50 (0.05, 5.52) | HR 0.52 (0.05, 5.83) | 0.598 |
| SES | 47.0 (47.0, 47.0) | 48.9 (47.6, 50.3) | HR 0.50 (0.05, 5.52) | HR 0.42 (0.04, 4.76) | 0.486 |
| Excluding the second child in cases of women having more than one pregnancy within the study. | 47.0 (47.0, 47.0) | 48.9 (47.6, 50.3) | HR 0.50 (0.05, 5.53) | HR 0.52 (0.05, 5.78) | 0.593 |
| **At least one admission for stroke, n/N (%)** | | | | | |
| Using per protocol analysis | 1/482 (0.2) | 1/489 (0.2) | RR 1.01 (0.06, 16.23) | RR 1.63 (0.09, 30.83) | 0.743 |
| Including additional covariates in the statistical model^1^ | | | | | |
| BMI | 1/493 (0.2) | 2/494 (0.4) | RR 0.50 (0.05, 5.52) | RR 0.33 (0.03, 3.55) | 0.360 |
| Including GA at birth rather than GA at entry | 1/493 (0.2) | 2/494 (0.4) | RR 0.50 (0.05, 5.52) | RR 0.52 (0.05, 5.79) | 0.597 |
| Birthweight Z score | 1/493 (0.2) | 2/494 (0.4) | RR 0.50 (0.05, 5.52) | RR 0.52 (0.05, 5.84) | 0.598 |
| SES | 1/493 (0.2) | 2/494 (0.4) | RR 0.50 (0.05, 5.52) | RR 0.42 (0.04, 4.74) | 0.485 |
| Excluding the second child in cases of women having more than one pregnancy within the study. | 1/481 (0.2) | 2/483 (0.4) | RR 0.50 (0.05, 5.54) | RR 0.52 (0.04, 6.04) | 0.593 |
| **Age at first heart failure admission, years, median (5^th^, 95^th^ centiles)** | | | | | |
| Using per protocol analysis | 41.8 (35.7, 48.5) | 40.3 (24.2, 47.1) | HR 0.43 (0.11, 1.67) | HR 0.43 (0.11, 1.67) | 0.222 |
| Including additional covariates in the statistical model^1^ | | | | | |
| BMI | 41.8 (35.7, 48.5) | 40.3 (24.2, 47.1) | HR 0.43 (0.11, 1.65) | HR 0.37 (0.06, 2.34) | 0.291 |
| Including GA at birth rather than GA at entry | 41.8 (35.7, 48.5) | 40.3 (24.2, 47.1) | HR 0.43 (0.11, 1.65) | HR 0.45 (0.12, 1.74) | 0.246 |
| Birthweight Z score | 41.8 (35.7, 48.5) | 40.3 (24.2, 47.1) | HR 0.43 (0.11, 1.65) | HR 0.43 (0.11, 1.65) | 0.216 |
| SES | 41.8 (35.7, 48.5) | 40.3 (24.2, 47.1) | HR 0.43 (0.11, 1.65) | HR 0.78 (0.11, 5.75) | 0.807 |
| Excluding the second child in cases of women having more than one pregnancy within the study. | 41.8 (35.7, 48.5) | 40.3 (24.2, 47.1) | HR 0.43 (0.11, 1.66) | HR 0.43 (0.11, 1.65) | 0.216 |
| **At least one admission for heart failure, n/N (%)** | | | | | |
| Using per protocol analysis | 3/482 (0.6) | 7/489 (1.4) | RR 0.43 (0.11, 1.67) | RR 0.43 (0.11, 1.71) | 0.227 |
| Including additional covariates in the statistical model^1^ | | | | | |
| BMI | 4/493 (0.6) | 7/494 (1.4) | RR 0.43 (0.11, 1.65) | RR 0.44 (0.08, 2.37) | 0.339 |
| Including GA at birth rather than GA at entry | 4/493 (0.6) | 7/494 (1.4) | RR 0.43 (0.11, 1.65) | RR 0.45 (0.12, 1.74) | 0.247 |
| Birthweight Z score | 4/493 (0.6) | 7/494 (1.4) | RR 0.43 (0.11, 1.65) | RR 0.43 (0.11, 1.65) | 0.217 |
| SES | 4/493 (0.6) | 7/494 (1.4) | RR 0.43 (0.11, 1.65) | RR 0.80 (0.11, 5.79) | 0.823 |
| Excluding the second child in cases of women having more than one pregnancy within the study. | 3/481 (0.6) | 7/483 (1.5) | RR 0.43 (0.11, 1.66) | RR 0.43 (0.11, 1.70) | 0.223 |
| **Time to death after randomization, days, median (5^th^, 95^th^ centiles)** | | | | | |
| Using per protocol analysis | 10.5 (0.4, 12850) | 3.8 (0.3, 17401) | HR 0.87 (0.69, 1.09) | HR 0.79 (0.63, 0.99) | 0.042 |
| Including additional covariates in the statistical model^1^ | | | | | |
| BMI* |  |  |  |  |  |
| Including GA at birth rather than GA at entry | 10.5 (0.4, 12849.6) | 4.0 (0.3, 16902.0) | HR 0.84 (0.67, 1.05) | HR 0.76 (0.61, 0.95) | 0.017 |
| Birthweight Z score | 10.5 (0.4, 12849.6) | 4.0 (0.3, 16902.0) | HR 0.84 (0.67, 1.05) | HR 0.76 (0.61, 0.96) | 0.022 |
| SES* |  |  |  |  |  |
| Excluding the second child in cases of women having more than one pregnancy within the study. | 10.5 (0.4, 12849.6) | 3.8 (0.3, 17151.5) | HR 0.85 (0.68, 1.07) | HR 0.82 (0.66, 1.04) | 0.095 |
| **Time to MACE after randomization (excluding cardiovascular death), days, median (5^th^, 95^th^ centiles)** | | | | | |
| Using per protocol analysis | 15351 (13132, 17718) | 15405 (8874, 17376) | HR 0.61 (0.22, 1.67) | HR 0.64 (0.23, 1.76) | 0.385 |
| Including additional covariates in the statistical model^1^ | | | | | |
| BMI | 15351 (13132, 17718) | 16064 (8874, 18444) | HR 0.54 (0.20, 1.46) | HR 0.44 (0.12, 1.58) | 0.209 |
| Including GA at birth rather than GA at entry | 15351 (13132, 17718) | 16064 (8874, 18444) | HR 0.54 (0.20, 1.46) | HR 0.55 (0.20, 1.50) | 0.244 |
| Birthweight Z score | 15351 (13132, 17718) | 16064 (8874, 18444) | HR 0.54 (0.20, 1.46) | HR 0.55 (0.20, 1.50) | 0.246 |
| SES | 15351 (13132, 17718) | 16064 (8874, 18444) | HR 0.54 (0.20, 1.46) | HR 0.84 (0.24, 2.94) | 0.785 |
| Excluding the second child in cases of women having more than one pregnancy within the study. | 15351 (13132, 17718) | 16064 (8874, 18444) | HR 0.54 (0.20, 1.47) | HR 0.56 (0.21, 1.52) | 0.252 |
| **Number of admissions to hospital with respiratory illness as primary reason for admission (admissions since 1988), mean (SD)** | | | | | |
| Using per protocol analysis | 0.1 (0.7) | 0.2 (0.7) | MD -0.1 (-0.2, 0.0) | MD -0.1 (-0.2, 0.1) | 0.356 |
| Including additional covariates in the statistical model^1^ | | | | | |
| BMI | Not applicable (BMI not available for deceased participants). | | | | |
| Including GA at birth rather than GA at entry | 0.2 (0.7) | 0.3 (1.9) | MD -0.2 (-0.4, 0.1) | MD -0.2 (-0.4, 0.1) | 0.200 |
| Birthweight Z score | 0.2 (0.7) | 0.3 (1.9) | MD -0.2 (-0.4, 0.1) | MD -0.2 (-0.4, 0.1) | 0.177 |
| SES | Not applicable (Current SES not available for deceased participants). | | | | |
| Excluding the second child in cases of women having more than one pregnancy within the study. | 0.2 (0.7) | 0.3 (1.9) | MD -0.2 (-0.5, 0.1) | MD -0.2 (-0.5, 0.1) | 0.225 |
| **Self-reported general health fair/poor, n/N (%)** | | | | | |
| Using per protocol analysis | 35/217 (16.1) | 23/187 (12.3) | RR 1.31 (0.80, 2.14) | RR 1.38 (0.82, 2.34) | 0.211 |
| Including additional covariates in the statistical model^1^ | | | | | |
| BMI | 37/223 (16.6) | 24/191 (12.6) | RR 1.32 (0.82, 2.13) | RR 1.04 (0.64, 1.70) | 0.866 |
| Including GA at birth rather than GA at entry | 37/223 (16.6) | 24/191 (12.6) | RR 1.32 (0.82, 2.13) | RR 1.32 (0.82, 2.14) | 0.252 |
| Birthweight Z score | 37/223 (16.6) | 24/191 (12.6) | RR 1.32 (0.82, 2.13) | RR 1.34 (0.83, 2.17) | 0.226 |
| SES | 37/223 (16.6) | 24/191 (12.6) | RR 1.32 (0.82, 2.13) | RR 1.29 (0.80, 2.09) | 0.292 |
| Excluding the second child in cases of women having more than one pregnancy within the study. | 37/217 (17.1) | 23/187 (12.3) | RR 1.39 (0.85, 2.25) | RR 1.43 (0.85, 2.42) | 0.164 |
| **Time in hospital after 1988, days per 10 years alive, median (5^th^, 95^th^ centiles)** | | | | | |
| Using per protocol analysis | 5.5 (20.2) | 3.9 (6.5) | MD 1.6 (-1.3, 4.4) | MD 1.8 (-1.3, 4.9) | 0.243 |
| Including additional covariates in the statistical model^1^ | | | | | |
| BMI | 5.4 (19.9) | 4.2 (7.1) | MD 1.2 (-1.6, 4.1) | MD 1.6 (-2.1, 5.3) | 0.393 |
| Including GA at birth rather than GA at entry | 5.4 (19.9) | 4.2 (7.1) | MD 1.2 (-1.6, 4.1) | MD 1.7 (-1.2, 4.5) | 0.254 |
| Birthweight Z score | 5.4 (19.9) | 4.2 (7.1) | MD 1.2 (-1.6, 4.1) | MD 1.5 (-1.4, 4.3) | 0.321 |
| SES | 5.4 (19.9) | 4.2 (7.1) | MD 1.2 (-1.6, 4.1) | MD 1.7 (-1.6, 5.1) | 0.310 |
| Excluding the second child in cases of women having more than one pregnancy within the study. | 4.7 (17.2) | 4.3 (7.2) | MD 0.5 (-2.1, 3.0) | MD 0.7 (-2.0, 3.5) | 0.578 |
| **Ischemic heart disease (combined hierarchical outcome), n/N (%)** | | | | | |
| Using per protocol analysis | 5/223 (2.2) | 9/191 (4.7) | RR0.48 (0.16, 1.40) | RR 0.46 (0.14, 1.47) | 0.175 |
| Including additional covariates in the statistical model^1^ | | | | | |
| BMI | 5/229 (2.2) | 9/195 (4.6) | RR 0.47 (0.16, 1.39) | RR 0.65 (0.18, 2.34) | 0.505 |
| Including GA at birth rather than GA at entry | 5/229 (2.2) | 9/195 (4.6) | RR 0.47 (0.16, 1.39) | RR 0.44 (0.15, 1.28) | 0.132 |
| Birthweight Z score | 5/229 (2.2) | 9/195 (4.6) | RR 0.47 (0.16, 1.39) | RR 0.46 (0.16, 1.35) | 0.155 |
| SES | 5/229 (2.2) | 9/195 (4.6) | RR 0.47 (0.16, 1.39) | RR 0.44 (0.15, 1.30) | 0.137 |
| Excluding the second child in cases of women having more than one pregnancy within the study. | 5/223 (2.2) | 9/191 (4.7) | RR 0.48 (0.16, 1.40) | RR 0.46 (0.14, 1.47) | 0.174 |
| **Stroke (combined hierarchical outcome), n/N (%)** | | | | | |
| Using per protocol analysis | 1/223 (0.5) | 2/191 (1.1) | RR 0.43 (0.04, 4.72) | RR 0.66 (0.05, 9.48) | 0.745 |
| Including additional covariates in the statistical model^1^ | | | | | |
| BMI | 1/229 (0.4) | 3/195 (1.5) | RR 0.28 (0.03, 2.72) | RR 0.26 (0.03, 2.46) | 0.239 |
| Including GA at birth rather than GA at entry | 1/229 (0.4) | 3/195 (1.5) | RR 0.28 (0.03, 2.72) | RR 0.29 (0.03, 2.78) | 0.281 |
| Birthweight Z score | 1/229 (0.4) | 3/195 (1.5) | RR 0.28 (0.03, 2.72) | RR 0.29 (0.03, 2.86) | 0.291 |
| SES | 1/229 (0.4) | 3/195 (1.5) | RR 0.28 (0.03, 2.72) | RR 0.31 (0.03, 3.01) | 0.312 |
| Excluding the second child in cases of women having more than one pregnancy within the study. | 1/223 (0.5) | 3/191 (1.6) | RR 0.29 (0.03, 2.74) | RR 0.30 (0.03, 3.49) | 0.311 |
| **Peripheral vascular disease (combined hierarchical outcome)** | | | | | |
| No sensitivity analysis performed as no events occurred in primary analysis. | | | | | |
| **Weight, mean (SD)** | | | | | |
| Using per protocol analysis | 89.0 (21.2) | 84.1 (19.2) | MD 4.9 (0.7, 9.1) | MD 4.0 (-0.2, 8.3) | 0.063 |
| Including additional covariates in the statistical model^1^ | | | | | |
| BMI |  |  |  |  |  |
| Including GA at birth rather than GA at entry | 88.8 (21.2) | 84.9 (20.0) | MD 4.0 (-0.2, 8.1) | MD 2.7 (-1.2, 6.6) | 0.177 |
| Birthweight Z score | 88.8 (21.2) | 84.9 (20.0) | MD 4.0 (-0.2, 8.1) | MD 2.4 (-1.5, 6.3) | 0.226 |
| SES | 88.8 (21.2) | 84.9 (20.0) | MD 4.0 (-0.2, 8.1) | MD 2.6 (-1.4, 6.6) | 0.205 |
| Excluding the second child in cases of women having more than one pregnancy within the study. | 88.7 (21.4) | 84.8 (20.1) | MD 3.9 (-0.3, 8.2) | MD 2.9 (-1.5, 7.4) | 0.177 |
| **Height, mean (SD)** | | | | | |
| Using per protocol analysis | 172.7 (9.9) | 170.1 (10.3) | MD 2.6 (0.5, 4.7) | MD 1.4 (-0.3, 3.2) | 0.104 |
| Including additional covariates in the statistical model^1^ | | | | | |
| BMI |  |  |  |  |  |
| Including GA at birth rather than GA at entry | 172.8 (10.0) | 170.2 (10.3) | MD 2.5 (0.5, 4.6) | MD 1.4 (-0.3, 3.2) | 0.092 |
| Birthweight Z score | 172.8 (10.0) | 170.2 (10.3) | MD 2.5 (0.5, 4.6) | MD 1.0 (-0.6, 2.7) | 0.203 |
| SES | 172.8 (10.0) | 170.2 (10.3) | MD 2.5 (0.5, 4.6) | MD 1.4 (-0.2, 3.0) | 0.095 |
| Excluding the second child in cases of women having more than one pregnancy within the study. | 172.8 (9.8) | 170.1 (10.3) | MD 2.7 (0.6, 4.8) | MD 1.3 (-0.5, 3.0) | 0.146 |
| **BMI in kg/m^2^, mean (SD)** | | | | | |
| Using per protocol analysis | 29.8 (6.5) | 28.8 (6.0) | MD 1.0 (-0.3, 2.3) | MD 1.2 (-0.3, 2.6) | 0.116 |
| Including additional covariates in the statistical model^1^ | | | | | |
| BMI |  |  |  |  |  |
| Including GA at birth rather than GA at entry | 29.7 (6.4) | 29.0 (6.2) | MD 0.7 (-0.6, 2.0) | MD 0.6 (-0.7, 1.9) | 0.369 |
| Birthweight Z score | 29.7 (6.4) | 29.0 (6.2) | MD 0.7 (-0.6, 2.0) | MD 0.6 (-0.7, 1.9) | 0.365 |
| SES | 29.7 (6.4) | 29.0 (6.2) | MD 0.7 (-0.6, 2.0) | MD 0.5 (-0.8, 1.9) | 0.433 |
| Excluding the second child in cases of women having more than one pregnancy within the study. | 29.6 (6.4) | 29.0 (6.2) | MD 0.6 (-0.7, 2.0) | MD 0.8 (-0.7, 2.3) | 0.277 |
| **Overweight or obesity, n/N (%)** | | | | | |
| Using per protocol analysis | 149/192 (77.6) | 110/156 (70.5) | RR 1.10 (0.97, 1.25) | RR 1.07 (0.93, 1.24) | 0.283 |
| Including additional covariates in the statistical model^1^ | | | | | |
| BMI |  |  |  |  |  |
| Including GA at birth rather than GA at entry | 152/198 (76.8) | 114/160 (71.3) | RR 1.08 (0.95, 1.22) | RR 1.04 (0.92, 1.19) | 0.506 |
| Birthweight Z score | 152/198 (76.8) | 114/160 (71.3) | RR 1.08 (0.95, 1.22) | RR 1.06 (0.93, 1.20) | 0.398 |
| SES | 152/198 (76.8) | 114/160 (71.3) | RR 1.08 (0.95, 1.22) | RR 1.04 (0.92, 1.19) | 0.479 |
| Excluding the second child in cases of women having more than one pregnancy within the study. | 146/192 (76.0) | 111/157 (70.7) | RR 1.08 (0.95, 1.22) | RR 1.05 (0.91, 1.21) | 0.466 |
| **Overweight, n/N (%)** | | | | | |
| Using per protocol analysis | 71/192 (37.0) | 59/156 (37.8) | RR 0.98 (0.74, 1.29) | RR 0.91 (0.68, 1.22) | 0.490 |
| Including additional covariates in the statistical model^1^ | | | | | |
| BMI |  |  |  |  |  |
| Including GA at birth rather than GA at entry | 73/198 (36.9) | 59/160 (36.9) | RR 1.00 (0.76, 1.31) | RR 0.96 (0.73, 1.27) | 0.794 |
| Birthweight Z score | 73/198 (36.9) | 59/160 (36.9) | RR 1.00 (0.76, 1.31) | RR 0.95 (0.72, 1.25) | 0.697 |
| SES | 73/198 (36.9) | 59/160 (36.9) | RR 1.00 (0.76, 1.31) | RR 0.98 (0.73, 1.31) | 0.883 |
| Excluding the second child in cases of women having more than one pregnancy within the study. | 70/192 (36.5) | 57/157 (36.3) | RR 1.00 (0.76, 1.33) | RR 0.95 (0.70, 1.29) | 0.731 |
| **Obesity, n/N (%)** | | | | | |
| Using per protocol analysis | 78/192 (40.6) | 51/156 (32.7) | RR 1.24 (0.94, 1.65) | RR 1.25 (0.92, 1.70) | 0.136 |
| Including additional covariates in the statistical model^1^ | | | | | |
| BMI |  |  |  |  |  |
| Including GA at birth rather than GA at entry | 79/198 (39.9) | 55/160 (34.4) | RR 1.16 (0.88, 1.53) | RR 1.16 (0.88, 1.52) | 0.299 |
| Birthweight Z score | 79/198 (39.9) | 55/160 (34.4) | RR 1.16 (0.88, 1.53) | RR 1.15 (0.88, 1.51) | 0.316 |
| SES | 79/198 (39.9) | 55/160 (34.4) | RR 1.16 (0.88, 1.53) | RR 1.13 (0.86, 1.49) | 0.387 |
| Excluding the second child in cases of women having more than one pregnancy within the study. | 76/192 (39.6) | 54/157 (34.4) | RR 1.15 (0.87, 1.52) | RR 1.16 (0.86, 1.57) | 0.295 |
| **Death from any cause, n/N (%)** | | | | | |
| Using per protocol analysis | 139/590 (23.6) | 160/607 (26.4) | RR 0.89 (0.73, 1.09) | RR 0.92 (0.77, 1.10) | 0.346 |
| Including additional covariates in the statistical model^1^ | | | | | |
| BMI† |  |  |  |  |  |
| Including GA at birth rather than GA at entry | 139/601 (23.1) | 165/617 (26.7) | RR 0.86 (0.71, 1.05) | RR 0.89 (0.73, 1.08) | 0.221 |
| Birthweight Z score | 139/601 (23.1) | 165/617 (26.7) | RR 0.86 (0.71, 1.05) | RR 0.85 (0.71, 1.01) | 0.064 |
| SES† |  |  |  |  |  |
| Excluding the second child in cases of women having more than one pregnancy within the study. | 137/587 (23.3) | 161/604 (26.7) | RR 0.88 (0.72, 1.07) | RR 0.90 (0.74, 1.08) | 0.258 |
| **Chronic respiratory illness (Self-reported diagnosis of asthma, COPD IPAG questionnaire >19.5, admissions for asthma or COPD or prescription of pharmaceuticals for asthma or COPD), n/N (%)** | | | | | |
| Using per protocol analysis | 74/223 (33.2) | 64/191 (33.5) | RR 0.99 (0.75, 1.30) | RR 1.01 (0.76, 1.36) | 0.922 |
| Including additional covariates in the statistical model^1^ | | | | | |
| BMI | 77/229 (33.6) | 66/195 (33.9) | RR 0.99 (0.76, 1.30) | RR 0.96 (0.71, 1.28) | 0.763 |
| Including GA at birth rather than GA at entry | 77/229 (33.6) | 66/195 (33.9) | RR 0.99 (0.76, 1.30) | RR 1.00 (0.77, 1.31) | 0.973 |
| Birthweight Z score | 77/229 (33.6) | 66/195 (33.9) | RR 0.99 (0.76, 1.30) | RR 1.03 (0.78, 1.35) | 0.852 |
| SES | 77/229 (33.6) | 66/195 (33.9) | RR 0.99 (0.76, 1.30) | RR 1.04 (0.78, 1.38) | 0.800 |
| Excluding the second child in cases of women having more than one pregnancy within the study. | 75/223 (33.6) | 64/191 (33.5) | RR 1.00 (0.76, 1.32) | RR 1.02 (0.77, 1.37) | 0.861 |
| **Asthma, n/N (%)** | | | | | |
| Using per protocol analysis | 73/229 (32.7) | 63/191 (33.0) | RR 0.99 (0.75, 1.31) | RR 1.01 (0.75, 1.35) | 0.960 |
| Including additional covariates in the statistical model^1^ | | | | | |
| BMI | 76/229 (33.2) | 65/195 (33.3) | RR 1.00 (0.76, 1.31) | RR 0.96 (0.71, 1.28) | 0.763 |
| Including GA at birth rather than GA at entry | 76/229 (33.2) | 65/195 (33.3) | RR 1.00 (0.76, 1.31) | RR 1.00 (0.76, 1.31) | 0.985 |
| Birthweight Z score | 76/229 (33.2) | 65/195 (33.3) | RR 1.00 (0.76, 1.31) | RR 1.02 (0.77, 1.34) | 0.893 |
| SES | 76/229 (33.2) | 65/195 (33.3) | RR 1.00 (0.76, 1.31) | RR 1.03 (0.77, 1.37) | 0.830 |
| Excluding the second child in cases of women having more than one pregnancy within the study. | 74/223 (33.2) | 63/191 (33.0) | RR 1.01 (0.76, 1.33) | RR 1.02 (0.76, 1.37) | 0.894 |
| **COPD diagnosis, n/N (%)** | | | | | |
| Using per protocol analysis | 4/223 (1.8) | 4/191 (2.1) | RR 0.86 (0.22, 3.39) | RR 1.10 (0.25, 4.75) | 0.897 |
| Including additional covariates in the statistical model^1^ | | | | | |
| BMI | 4/229 (1.8) | 4/195 (2.1) | RR 0.85 (0.21, 3.37) | RR 0.85 (0.17, 4.16) | 0.839 |
| Including GA at birth rather than GA at entry | 4/229 (1.8) | 4/195 (2.1) | RR 0.85 (0.21, 3.37) | RR 0.94 (0.24, 3.73) | 0.934 |
| Birthweight Z score | 4/229 (1.8) | 4/195 (2.1) | RR 0.85 (0.21, 3.37) | RR 1.09 (0.27, 4.37) | 0.908 |
| SES | 4/229 (1.8) | 4/195 (2.1) | RR 0.85 (0.21, 3.37) | RR 1.50 (0.33, 6.70) | 0.598 |
| Excluding the second child in cases of women having more than one pregnancy within the study. | 4/223 (1.8) | 4/191 (2.1) | RR 0.86 (0.22, 3.39) | RR 1.10 (0.25, 4.78) | 0.896 |
| **IPAG COPD questionnaire score >19.5, n/N (%)** | | | | | |
| Using per protocol analysis | 3/175 (1.7) | 2/144 (1.4) | RR 1.23 (0.21, 7.34) | RR 1.23 (0.16, 9.2) | 0.827 |
| Including additional covariates in the statistical model^1^ | | | | | |
| BMI |  |  |  |  |  |
| Including GA at birth rather than GA at entry | 3/179 (1.7) | 2/147 (1.4) | RR 1.23 (0.21, 7.32) | RR 1.15 (0.19, 6.83) | 0.880 |
| Birthweight Z score | 3/179 (1.7) | 2/147 (1.4) | RR 1.23 (0.21, 7.32) | RR 1.20 (0.20, 7.23) | 0.842 |
| SES | 3/179 (1.7) | 2/147 (1.4) | RR 1.23 (0.21, 7.32) | RR 0.77 (0.11, 5.50) | 0.791 |
| Excluding the second child in cases of women having more than one pregnancy within the study. | 3/174 (1.7) | 2/144 (1.4) | RR 1.24 (0.21, 7.38) | RR 1.23 (0.16, 9.25) | 0.824 |
| **Proportion with at least one admission for asthma or chronic obstructive pulmonary disease (COPD), n/N (%)** | | | | | |
| Using per protocol analysis | 18/217 (8.3) | 15/184 (8.2) | RR 1.02 (0.53, 1.97) | RR 1.08 (0.53, 2.22) | 0.822 |
| Including additional covariates in the statistical model^1^ | | | | | |
| BMI | 20/223 (9.0) | 16/188 (8.5) | RR 1.05 (0.56, 1.98) | RR 1.09 (0.54, 2.18) | 0.810 |
| Including GA at birth rather than GA at entry | 20/223 (9.0) | 16/188 (8.5) | RR 1.05 (0.56, 1.98) | RR 1.13 (0.60, 2.13) | 0.704 |
| Birthweight Z score | 20/223 (9.0) | 16/188 (8.5) | RR 1.05 (0.56, 1.98) | RR 1.17 (0.62, 2.22) | 0.630 |
| SES | 20/223 (9.0) | 16/188 (8.5) | RR 1.05 (0.56, 1.98) | RR 1.25 (0.64, 2.44) | 0.513 |
| Excluding the second child in cases of women having more than one pregnancy within the study. | 20/218 (9.2) | 16/184 (8.7) | RR 1.06 (0.56, 1.98) | RR 1.12 (0.56, 2.25) | 0.725 |
| **Prescriptions of pharmaceuticals for asthma or COPD, n/N (%)** | | | | | |
| Using per protocol analysis | 66/213 (31.0) | 46/179 (25.7) | RR 1.21 (0.87, 1.66) | RR 1.28 (0.90, 1.81) | 0.152 |
| Including additional covariates in the statistical model^1^ | | | | | |
| BMI | 69/219 (31.5) | 47/182 (25.8) | RR 1.22 (0.89, 1.67) | RR 1.32 (0.94, 1.87) | 0.113 |
| Including GA at birth rather than GA at entry | 69/219 (31.5) | 47/182 (25.8) | RR 1.22 (0.89, 1.67) | RR 1.25 (0.91, 1.71) | 0.165 |
| Birthweight Z score | 69/219 (31.5) | 47/182 (25.8) | RR 1.22 (0.89, 1.67) | RR 1.29 (0.94, 1.77) | 0.121 |
| SES | 69/219 (31.5) | 47/182 (25.8) | RR 1.22 (0.89, 1.67) | RR 1.28 (0.92, 1.77) | 0.141 |
| Excluding the second child in cases of women having more than one pregnancy within the study. | 67/214 (31.3) | 46/179 (25.7) | RR 1.22 (0.89, 1.68) | RR 1.30 (0.92, 1.85) | 0.123 |
| **Excluding participants for whom there was a known family history of diabetes mellitus noted at 30-year follow-up** |  | | | | |
| Diabetes mellitus, prediabetes or gestational diabetes mellitus, n/N (%) | 40/205 (19.5) | 30/172 (17.4) | RR 1.12 (0.73, 1.72) | RR 1.15 (0.73, 1.83) | 0.518 |
| Diabetes mellitus (any type), n/N (%) | 11/205 (5.4) | 13/172 (7.6) | RR 0.71 (0.33, 1.55) | RR 0.69 (0.3, 1.63) | 0.371 |
| Type 2 diabetes mellitus, n/N (%) | 11/205 (5.4) | 10/172 (5.8) | RR 0.92 (0.4, 2.13) | RR 0.86 (0.35, 2.13) | 0.734 |
| Type 1 diabetes mellitus, n/N (%) | Analysis not performed as only 1 participant had T1DM in primary analysis. | | | | |
| Prediabetes, n/N (%) | 26/204 (12.8) | 16/171 (9.4) | RR 1.36 (0.75, 2.46) | RR 1.41 (0.74, 2.70) | 0.272 |
| Gestational diabetes mellitus, n/N (%) | 5/91 (5.5) | 2/90 (2.2) | RR 2.47 (0.49, 12.55) | RR 2.67 (0.27, 26.33) | 0.299 |

^1^ Additional covariates include current BMI, current socioeconomic status, gestation at birth and birthweight z score.

*Analyses adjusted for sex, gestational age at trial entry and for clustering.

†Sensitivity analysis not performed as BMI and socioeconomic status as outcome events predominantly occurred before available BMI and socioeconomic status data.

BMI: Body mass index. CI: Confidence interval. COPD: Chronic obstructive pulmonary disease. GA: gestational age. HR: Hazard ratio. IPAG: International primary care airways group. MACE: Major adverse cardiovascular event. MD: Mean difference. RR: Relative risk. SES: Socioeconomic status quintiles as assessed by New Zealand Deprivation index 2018 or Socioeconomic Index for Australia. SD: Standard deviation.

Table F: Post hoc subgroup analysis for primary outcomes and components by gestational age at delivery

| **Outcome and sub-group** | **Betamethasone** | **Placebo** | **Unadjusted RR or HR (95% CI)** | **Adjusted* RR or HR (95% CI)** | **P value**  **(adjusted*)** | **Interaction P value** |
| --- | --- | --- | --- | --- | --- | --- |
| **Cardiometabolic risk factor composite, n/N (%)** | | | | | | |
| **Preterm birth** |  | | | | | 0.174 |
| Preterm | 115/166 (69.3) | 97/135 (71.9) | RR 0.96 (0.83, 1.12) | RR 0.96 (0.83, 1.11) | 0.592 |  |
| Term | 41/63 (65.1) | 32/60 (53.3) | RR 1.22 (0.90, 1.65) | RR 1.20 (0.90, 1.60) | 0.213 |  |
| **Gestational age at delivery continuous outcome** |  | | | | | 0.310 |
| **Age at first major adverse cardiovascular event, median (5^th^, 95^th^ centile)** | | | | | | |
| **Preterm birth** |  | | | | | 0.196 |
| Preterm | 41.8 (20.5, 44.6) | 38.9 (24.2, 46.7) | HR 1.12 (0.30, 4.16) | HR 1.14 (0.31, 4.25) | 0.846 |  |
| Term | 42.1 (35.7, 48.5) | 45.1 (31.9, 50.3) | HR 0.29 (0.06, 1.37) | HR 0.30 (0.06, 1.41) | 0.127 |  |
| **Gestational age at delivery continuous outcome** |  | | | | | 0.187 |
| **Diabetes mellitus or prediabetes or gestational diabetes mellitus, n/N (%)** | | | | | | |
| **Preterm birth** |  | | | | | 0.198 |
| Preterm | 35/166 (21.1) | 33/135 (24.4) | RR 0.86 (0.57, 1.31) | RR 0.86 (0.56, 1.31) | 0.480 |  |
| Term | 13/63 (20.6) | 8/60 (13.3) | RR 1.55 (0.69, 3.50) | RR 1.56 (0.70, 3.51) | 0.280 |  |
| **Gestational age at delivery continuous outcome** |  |  |  |  |  | 0.402 |
| **Diabetes Mellitus (any type), n/N (%)** | | | | | | |
| **Preterm birth** |  | | | | | 0.657 |
| Preterm | 11/166 (6.6) | 16/135 (11.9) | RR 0.56 (0.27, 1.17) | RR 0.54 (0.26, 1.14) | 0.105 |  |
| Term | 4/63 (6.4) | 5/60 (8.3) | RR 0.76 (0.21, 2.74) | RR 0.76 (0.21, 2.70) | 0.665 |  |
| **Gestational age at delivery continuous outcome** |  | | | | | 0.757 |
| **Prediabetes, n/N (%)** | | | | | | |
| **Preterm birth** |  | | | | | 0.180 |
| Preterm | 21/166 (12.7) | 15/134 (11.2) | RR 1.13 (0.61, 2.11) | RR 1.11 (0.59, 2.09) | 0.740 |  |
| Term | 9/62 (14.5) | 3/60 (5.0) | RR 2.90 (0.82, 10.3) | RR 2.91 (0.82, 10.26) | 0.097 |  |
| **Gestational age at delivery continuous outcome** |  | | | | | 0.063 |
| **Gestational diabetes mellitus, n/N (%)** | | | | | | |
| **Preterm birth** |  | | | | | 0.973 |
| Preterm | 5/74 (6.8) | 3/74 (4.1) | RR 1.67 (0.41, 6.80) | RR 1.82 (0.45, 7.42) | 0.403 |  |
| Term | 2/31 (6.5) | 0/32 (0.0) | NE | NE | 0.972 |  |
| **Gestational age at delivery continuous outcome** |  | | | | | 0.863 |
| **Hypertension, n/N (%)** | | | | | | |
| **Preterm birth** |  | | | | | 0.183 |
| Preterm | 55/165 (33.3) | 53/135 (39.3) | RR 0.85 (0.63, 1.15) | RR 0.86 (0.63, 1.16) | 0.310 |  |
| Term | 16/62 (25.8) | 10/59 (17.0) | RR 1.52 (0.75, 3.10) | RR 1.42 (0.72, 2.80) | 0.315 |  |
| **Gestational age at delivery continuous outcome** |  | | | | | 0.937 |
| **Hyperlipidemia, n/N (%)** | | | | | |  |
| **Preterm birth** |  | | | | | 0.361 |
| Preterm | 90/164 (54.9) | 80/133 (60.2) | RR 0.91 (0.75, 1.11) | RR 0.93 (0.77, 1.12) | 0.441 |  |
| Term | 35/63 (55.6) | 30/60 (50.0) | RR 1.11 (0.79, 1.56) | RR 1.10 (0.80, 1.52) | 0.547 |  |
| **Gestational age at delivery continuous outcome** |  | | | | | 0.440 |

HR: Hazard ratio. RR: Relative risk.

*Analyses adjusted for sex and gestational age at trial entry.
